# Reversible transformations between the non-porous phases of a flexible coordination network enabled by transient porosity

**DOI:** 10.1038/s41557-022-01128-3

**Published:** 2023-02-13

**Authors:** Varvara I. Nikolayenko, Dominic C. Castell, Debobroto Sensharma, Mohana Shivanna, Leigh Loots, Katherine A. Forrest, Carlos J. Solanilla-Salinas, Ken-ichi Otake, Susumu Kitagawa, Leonard J. Barbour, Brian Space, Michael J. Zaworotko

**Affiliations:** 1 Department of Chemical Sciences, University of Limerick, Limerick, Republic of Ireland; 2 Bernal Institute, University of Limerick, Limerick, Republic of Ireland; 3 Institute for Integrated Cell-Material Sciences (iCeMS), Kyoto University Institute for Advanced Study (KUIAS), Kyoto University, Kyoto, Japan; 4 Department of Chemistry and Polymer Science, Stellenbosch University, Stellenbosch, South Africa; 5 Department of Chemistry, University of South Florida, Tampa, FL USA; 6 Department of Chemistry, North Carolina State University, Raleigh, NC USA

**Keywords:** Metal-organic frameworks, Crystal engineering

## Abstract

Flexible metal–organic materials that exhibit stimulus-responsive switching between closed (non-porous) and open (porous) structures induced by gas molecules are of potential utility in gas storage and separation. Such behaviour is currently limited to a few dozen physisorbents that typically switch through a breathing mechanism requiring structural contortions. Here we show a clathrate (non-porous) coordination network that undergoes gas-induced switching between multiple non-porous phases through transient porosity, which involves the diffusion of guests between discrete voids through intra-network distortions. This material is synthesized as a clathrate phase with solvent-filled cavities; evacuation affords a single-crystal to single-crystal transformation to a phase with smaller cavities. At 298 K, carbon dioxide, acetylene, ethylene and ethane induce reversible switching between guest-free and gas-loaded clathrate phases. For carbon dioxide and acetylene at cryogenic temperatures, phases showing progressively higher loadings were observed and characterized using in situ X-ray diffraction, and the mechanism of diffusion was computationally elucidated.

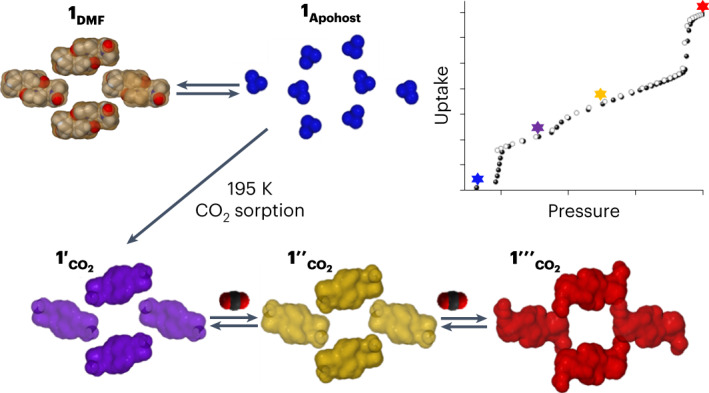

## Abstract (web summary)

Sorbent materials that reversibly transform between closed (non-porous) and open (porous) phases on the uptake and release of guests are relevant to gas storage and separation applications. Now, a coordination network has been prepared that exhibits gas-induced transformations between multiple non-porous phases. This phenomenon is attributed to subtle structural rearrangements that enable transient porosity.

## Main

**Fig. 1:**
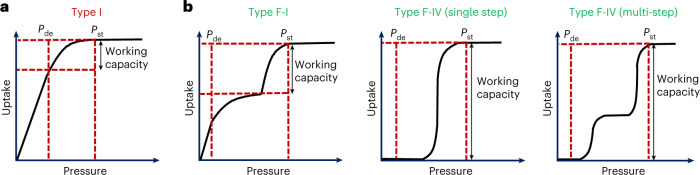
Representative types of gas sorption isotherms for different sorbents. **a**,**b**, Type I (rigid; **a**) and type F (flexible; **b**) materials^12^. The red dashed lines indicate the delivery pressure (*P*_de_) and storage pressure (*P*_st_). Type F-IV isotherms are highly sought after in gas storage applications as the closed-to-open transformation provides the highest working capacity.

Owing to their inherent modularity and resulting amenability to design, metal–organic materials^1,2^ such as metal–organic frameworks^3,4^ and porous coordination networks^5,6^ offer promise as energy-efficient physisorbents for gas separation and storage. Rigid microporous materials, which tend to exhibit fixed pore volumes and type I adsorption isotherms (Fig. 1a), have been classified as second-generation physisorbents^7^. A limitation of type I isotherms is that adsorption and desorption tend to occur at pressures outside the operationally relevant range, thus decreasing the working capacity^8^. Third- (soft, flexible frameworks characterized by stepped or S-shaped adsorption isotherms) and fourth-generation (porous materials with modifiable pore size, pore chemistry and self-switching pores) flexible metal–organic materials (FMOMs)^7,9–11^ offer more desirable type F sorption profiles (Fig. 1b). FMOMs exhibit structural transformations that can occur either between open and more open forms (Fig. 1b; type F-I) or between closed and open forms (Fig. 1b; type F-IV)^12^.

FMOMs are exemplified by stepped isotherms that arise when sudden switching occurs between a closed and an open phase with negligible sorption below the switching threshold pressure. Such materials are of potential utility as they offer higher working capacities for volatile gases that are normally stored in high-pressure cylinders (for example, O_2_, H_2_ and CH_4_) and C_2_H_2_, for which high-pressure storage is infeasible^13–20^. Nevertheless, nearly two decades have elapsed since the first reported switching sorbent^21–23^, and a recent review^24^ confirmed that fewer than 100 materials exhibit substantial flexible breathing behaviour. Among these, only 60 can be defined as switching sorbents (closed-to-open transformations)^24–34^. Rarer still are switching materials (type F-IV isotherms) with multiple steps, indicating additional transformations beyond the initial gate opening^25,28,30,33^. Despite the potential for enhanced working capacity in gas storage applications, FMOMs that exhibit type F-IV adsorption isotherms are relatively rare and remain to be classified by the International Union of Pure and Applied Chemistry^24,35^. Supplementary Table 5 shows the current benchmark switching materials. Despite the impressive uptake in some of these materials, owing to the large structural transformations associated with their switching behaviour, very few were found to exhibit recyclability with retained performance^36^.

**Fig. 2:**
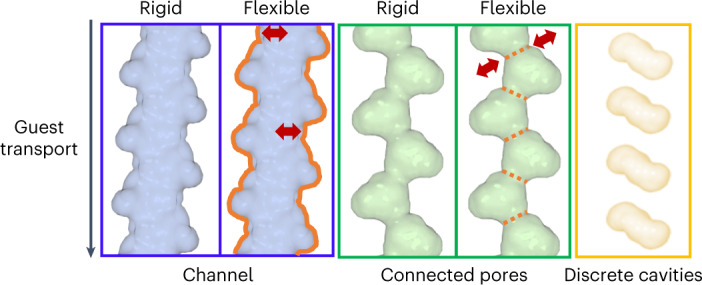
Three sorbate transport modes that are possible in porous materials. Left, continuous channels (shown as a blue surface), which can be rigid or flexible. Middle, connected pores (shown as a green surface), which can also be rigid or flexible. Right, discrete cavities (shown as a yellow surface). The red arrows and orange lines indicate the direction and nature of flexibility, respectively.

Guest transport in porous materials occurs via three pathways: (1) rigid or flexible channels (Fig. 2, left); (2) pores interconnected via narrow rigid or dynamic apertures^7,9–11^ (Fig. 2, middle); and (3) diffusion between discrete cavities (Fig. 2, right). The nature of the dynamic behaviour observed in third- and fourth-generation systems has been ascribed to structural transformation through various mechanisms (for example breathing, swelling, linker rotation or subnetwork displacement)^9,12^. Establishing a mechanism can be a challenge as single crystals comprise many mechanically responsive unit cells and the strain associated with flexible transformations can induce crystal fracturing. Therefore, only a few FMOMs are known to exhibit single-crystal to single-crystal transformations^20,33^.

The concept of transient porosity must be invoked to explain guest transport in clathrates (that is, crystals with discrete voids but without connecting channels; Fig. 2, right). This was considered counter-intuitive until the prototypal example was published by Atwood et al.^37^ in 2002. In this seminal study, the bowl-shaped molecule *p*-*tert*-butylcalix[4]arene was shown to include a guest molecule in discrete cavities despite the absence of channels. Subsequently, other non-porous molecular materials exhibiting dynamic and cooperative guest transport were reported^38–45^.

Cooper et al.^46^ defined such porosity as being either intrinsic or extrinsic, with the former arising from cavities in the molecular building block itself and the latter being a consequence of solid-state packing. Although this definition is suitable for molecular systems, it does not easily translate to extended networks. The Kitagawa and Barbour groups have independently reported sorbate transport in FMOMs with connected pores^47–49^ or discrete voids^50^, the latter arising from a sorbent that has an as-synthesized phase with a channel-type structure.

Herein, we expand on the work of Barbour et al. by introducing an example of a FMOM, [Co(dpt)(1,3-bib)], **1**, for which sorbate transport results from synergistic transient porosity that enables transformations between multiple non-porous phases. Specifically, **1** is a switching sorbent with non-porous, as-synthesized apohost (the guest-free framework) and gas-loaded clathrate phases. Furthermore, in addition to exhibiting a better working capacity compared with the previously reported [Co_2_(ndc)_2_(bpy)]_*n*_^50^, **1** exhibits switching in response to a number of different guests. Although the transformations in **1** are structurally subtle and associated with little to no strain on the framework, the switching is unambiguous and enables a working capacity that is higher than would be expected for a conventionally non-porous material. **1** also offers good recyclability and can serve as a prototypal structure for the development of future materials. In principle, this strategy can be evolved to include materials with much larger cavity sizes, to achieve a combination of high working capacity and sustained recyclability.

## Results and discussion

### Preparation and characterization of 1_DMF_

**Fig. 3:**
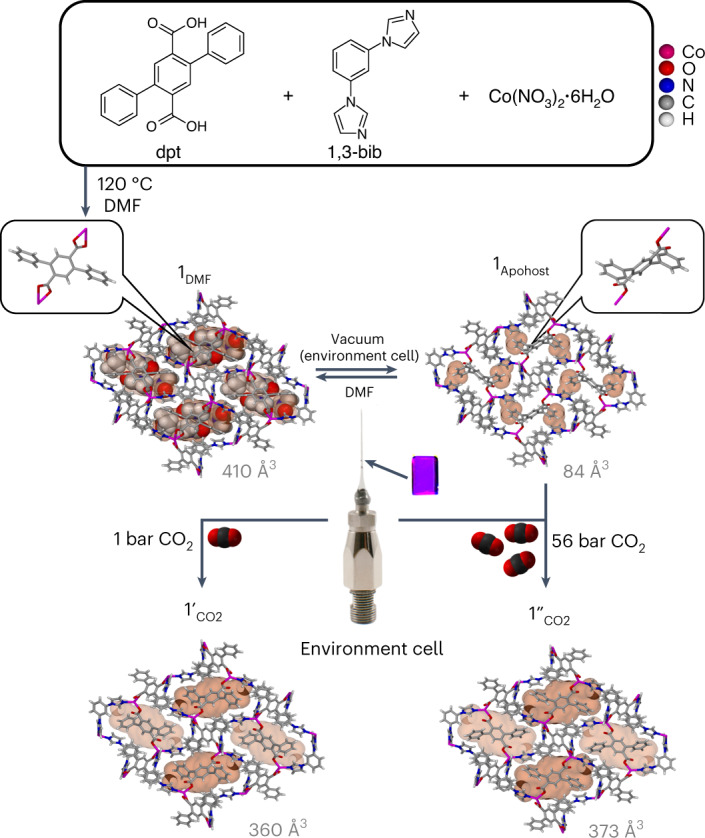
Synthesis and reversible structural transformations observed in 1. Solvothermal preparation of **1**_**DMF**_ from dpt, 1,3-bib and Co(NO_3_)_2_·6H_2_O yields purple crystals with *N*,*N*-DMF (shown in space-filling representation) located in discrete cavities of 410 Å^3^ (the cavities are shown as an orange surface). The inset on the left shows the position of the chelating dpt ligand. In situ activation of a single crystal of **1**_**DMF**_ (using an environment cell; see the corresponding photomicrograph) yields guest-free-phase **1**_**Apohost**_ where, as a result of rotation, the dpt ligand becomes mono-coordinated (see the inset on the right) and the guest-accessible cavities are reduced to 84 Å^3^. Subsequent gas loading of **1**_**Apohost**_ first with 1 bar of CO_2_ (bottom left) and then with 56 bar of CO_2_ (bottom right) leads to **1ʹ**_**CO2**_ (discrete cavities of 360 Å^3^) and **1′′**_**CO2**_ (discrete cavities of 373 Å^3^). Closer inspection of the cavities in **1ʹʹ**_**CO2**_ shows the cavities in very close proximity to one another, hinting at a potential transportation pathway. The CO_2_ gas molecules loaded into **1ʹ**_**CO2**_ and **1ʹʹ**_**CO2**_ could not be crystallographically modelled. The dpt disorder in **1**_**Apohost**_ and **1ʹ**_**CO2**_ has been omitted for clarity. Source data

Solvothermal reaction of Co(NO_3_)_2_·6H_2_O with 2,5‐diphenylbenzene‐1,4‐dicarboxylic acid (dpt) and 1,3-bis(1H-imidazol-1-yl)benzene) (1,3-bib) in dimethylformamide (DMF) yielded diffraction-quality crystals of [Co(dpt)(1,3-bib)٠2DMF] (**1**_**DMF**_; Fig. 3). Single-crystal X-ray diffraction (SCXRD) showed that **1**_**DMF**_ crystallized in the monoclinic space group P2_1_/*n*. The asymmetric unit (ASU) comprises one metal cation, one 1,3-bib ligand (anti-conformation), two independent half dpt ligands and two DMF guest molecules. One dpt ligand chelates the metal cation, whereas the second is mono-coordinate (Supplementary Fig. 1). The phenyl rings of the chelating dpt ligand were found to be disordered over two general positions with 0.74:0.26 site occupancy. The tetrahedral molecular building block is connected by the linker ligands to form a three-dimensional non-interpenetrated **dmp** topology net.

A Connolly map^51,52^ of the guest-accessible volume (using a 1.5 Å probe radius) in **1**_**DMF**_ indicated that at 100 K, it contains discrete solvent-filled voids of approximately 391 or 411 Å^3^ viewed down [100] (depending on the fragment of disorder used) (Supplementary Fig. 2). At 298 K, dpt ligands exhibit negligible disorder, with voids of 410 Å^3^ (Fig. 3). **1**_**DMF**_ is classified as a clathrate since it contains two intrinsic voids per unit cell, each accommodating four DMF molecules.

Thermogravimetric analysis confirmed that **1**_**DMF**_ lost all DMF guest molecules below 423 K and remained stable until framework decomposition at 603 K (Supplementary Fig. 13). Bulk-phase activation of **1**_**DMF**_ in air induced a colour change from purple to blue concomitant with a change in the powder X-ray diffraction (PXRD) pattern (Supplementary Figs. 9–10). The removal of DMF guest molecules from **1**_**DMF**_ resulted in a transformation to **1**_**Hydrate**_. Comparison of the two structures at 100 K revealed that the previously chelating dpt carboxylate moiety became mono-coordinated, the resulting open metal site being filled by an aqua ligand (Supplementary Fig. 4). This transformation was accompanied by a reduction in the *b* and *c* axis lengths (from 14.62(3) to 12.86(2) Å and from 23.67(5) to 22.01(5) Å, respectively; Supplementary Table 1) and a 16% reduction in unit cell volume. Mapping of the guest-accessible volume showed that **1**_**Hydrate**_ contained discrete cavities of 42 Å^3^.

### Preparation and characterization of 1_Apohost_

To avoid the formation of **1**_**Hydrate**_, a crystal of **1**_**DMF**_ was mounted in an environment gas cell, sealed and evacuated at 393 K overnight. A single-crystal to single-crystal transformation from **1**_**DMF**_ to **1**_**Apohost**_ (Fig. 3) was observed using SCXRD data recorded at 298 K. Although the space group was unchanged, activation induced 0.91(4) and 1.26(3) Å reduction in the *b* and *c* axes, respectively, and a 12% unit cell volume contraction (Supplementary Table 1). **1**_**Apohost**_ is similar to **1**_**Hydrate**_ in that the structural transformation arises from a change in the metal coordination environment (cobalt becoming 4-coordinate) and dpt linker rotation. We note that metal node isomerism involving coordination bond breakage is relatively rare but can play a key role in switching FMOMs^53,54^. The phenyl rings of the previously chelating dpt ligand were found to be disordered over two general positions with 0.54:0.46 site occupancies. However, unlike **1**_**Hydrate**_, **1**_**Apohost**_ has no aqua ligands. Comparison of the torsion angle O_1_C_13_C_14_C_15_ in **1**_**DMF**_ and **1**_**Apohost**_ indicated that, upon activation, one of the dpt ligands had rotated by 61.0(1)° from a coplanar to a twisted orientation (Supplementary Figs. 5 and 6). Although the 1,3-bib ligand retains its anti-conformation, there is a reduction in the N–Co–N angle from 105.4(6)° in **1**_**DMF**_ to 101.4(8)° in **1**_**Apohost**_. This combined motion reduces the void volume of **1**_**Apohost**_ to 84 Å^3^.

### Gas sorption of 1_Apohost_ at 298 K and 1 bar

**Fig. 4:**
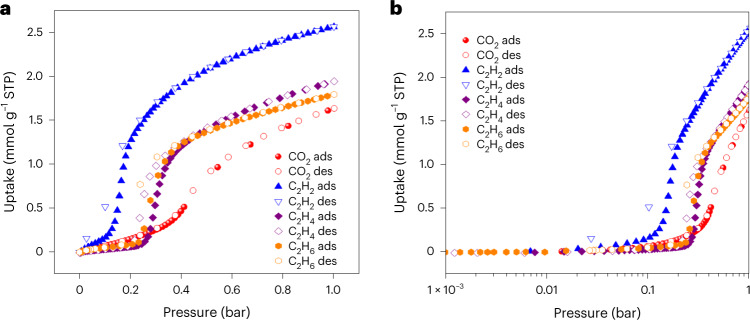
Gas sorption isotherms of 1_Apohost_ at 298 K and 1 bar. **a**, Sorption isotherms plotted using a linear scale. **b**, Sorption isotherms plotted using a logarithmic scale. Different gases are denoted as follows: CO_2_, red spheres; C_2_H_2_, blue triangles; C_2_H_4_, purple diamonds; C_2_H_6_, orange hexagons. **1**_**Apohost**_ undergoes a closed-to-open phase transition upon exposure to all four gases, with an inflection trend of C_2_H_2_ > C_2_H_6_ > C_2_H_4_ > CO_2_. ads, adsorption; des, desorption; STP, standard temperature and pressure. Source data

To investigate the gas-induced dynamic behaviour of **1**, a series of sorption studies were conducted. A bulk sample of **1**_**DMF**_ was activated to afford **1**_**Apohost**_; all sorption experiments used **1**_**Apohost**_ as the sorbent (Supplementary Fig. 11). Isotherms recorded at 298 K for CO_2_, C_2_H_2_, C_2_H_4_ and C_2_H_6_ showed that uptake to form a **1ʹ**_**gas**_ phase (where gas denotes the aforementioned gases) was induced by closed-to-open switching. The isotherms for C_2_H_2_, C_2_H_4_ and C_2_H_6_ exhibited steps at threshold pressures of 0.15, 0.20 and 0.24 bar, respectively (that is, type F-IV isotherms) (Fig. 4a,b). The CO_2_ isotherm was comparable in terms of its threshold pressure (0.3 bar), but the inflection was observed to be more gradual, corresponding to type F-III behaviour. This difference is emphasized in the first derivative of the adsorption curve (Supplementary Fig. 22), which can be contrasted with the sharper C_2_ gas profiles. We note that hysteresis between the adsorption and desorption branches of each isotherm was variable (Fig. 4a).

The CO_2_ isotherm displayed no discernible hysteresis; moderate hysteresis was observed for C_2_H_4_ and C_2_H_6_; and large hysteresis was seen for C_2_H_2_. The unusual isotherms exhibited by **1** do not conform to standard continuous isotherm models and were not conducive to accurate fitting by virial analysis (*R*_2_ < 70%) over the whole or partial loading ranges. Due to the low quality of parametrization, it was not feasible to determine physically meaningful isosteric heats of adsorption, as are conventionally reported for rigid adsorbents. Thermodynamic information on the nature of the transition was obtained from calculation of the enthalpy of gate opening (Δ*H*_GO_) from pure component data following the method of Li et al.^55^ (ESI, Section 7.4). Δ*H*_GO_ was determined to be 25.2 kJ mol^−1^ for CO_2_, 33.7 kJ mol^−1^ for C_2_H_2_, 29.8 kJ mol^−1^ for C_2_H_4_ and 31.7 kJ mol^−1^ for C_2_H_6_. The similar values of Δ*H*_GO_ exhibited by the C_2_ hydrocarbons and the lower value for CO_2_ help to explain the respective isotherm shapes and derivative profiles (Supplementary Fig. 28). The large enthalpy of gate opening in the presence of C_2_H_2_ is correlated with a lower threshold pressure, sharper derivative profile and greater degree of hysteresis. C_2_H_4_ and C_2_H_6_ show similar derivative profiles and intermediate threshold pressures, while CO_2_ shows the broadest derivative profile and highest threshold pressure, in agreement with their respective enthalpies of gate opening. The working capacities between 0.1 and 1 bar for CO_2_ and C_2_H_2_ at 298 K are 35.1 and 54.4 cm^3^ g^−1^, respectively. Sustained recyclability of **1**_**Apohost**_ was confirmed over 50 cycles using CO_2_ at 298 K in the range 0–30 bar (Supplementary Fig. 26).

### Structural insight into 1ʹ_CO2_

To understand the flexible behaviour of **1**_**Apohost**_ induced by CO_2_, a gas-loading in situ SCXRD experiment was performed. After backfilling the environment gas cell with CO_2_ to the maximum pressure recorded for the corresponding isotherm (1 bar, at 298 K), the system was left to equilibrate for 3 h before being sealed (see Supplementary Information for details). SCXRD data were then recorded. Conversion of **1**_**Apohost**_ to **1ʹ**_**CO2**_ occurred with an increase in the *b* and *c* axes (0.65(3) and 0.44(1) Å, respectively) and a 7% increase in the unit cell volume (Supplementary Table 1), resulting in discrete voids of 360 Å^3^. This transformation can once again be attributed to rotation and chelation of the dpt ligand (Supplementary Figs. 7 and 8). The 1,3-bib N–Co–N angle increased to 104.6(9)°. Although the CO_2_ guest could not be modelled crystallographically, the residual electron density analysis, as implemented by the SQUEEZE^56^ routine of PLATON^57^, indicated the presence of half a CO_2_ molecule per ASU.

### Cryogenic gas sorption isotherms of 1_Apohost_

**Fig. 5:**
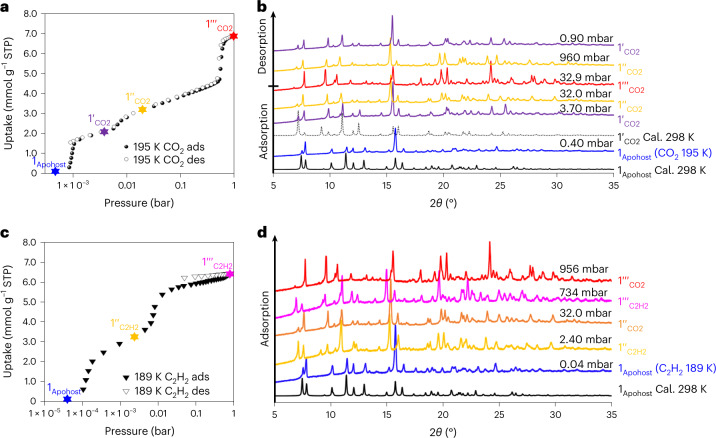
Cryogenic gas sorption isotherms and corresponding in situ PXRD profiles. **a**, 195 K CO_2_ sorption isotherm on **1**_**Apohost**_ plotted using a logarithmic scale. The coloured stars represent the four different phases present (blue is **1**_**Apohost**_, purple is **1ʹ**_**CO2**_, yellow is **1ʹʹ**_**CO2**_, red is **1ʹʹʹ**_**CO2**_). **b**, An overlay of in situ variable pressure PXRD patterns (*λ* = 1.54178 Å) of **1**_**Apohost**_ at different CO_2_ adsorption/desorption loadings recorded at 195 K. The patterns are colour coded to denote phases (Cal. = calculated from experimental X-ray crystal structure). **c**, 189 K C_2_H_2_ sorption isotherm on **1**_**Apohost**_ plotted using a logarithmic scale (blue is **1**_**Apohost**_, yellow is **1ʹʹ**_**C2H2**_ and pink is **1ʹʹʹ**_**C2H2**_). **d**, An overlay of in situ variable pressure PXRD patterns (*λ* = 1.54178 Å) of **1**_**Apohost**_ at different C_2_H_2_ adsorption/desorption loadings recorded at 189 K. The **1ʹʹʹ**_**C2H2**_ (pink star) phase is not the same as the **1ʹʹʹ**_**CO2**_ (red star) phase. Source data

Low-temperature isotherms (77, 195 and 189 K) were recorded using N_2_, CO_2_ and C_2_H_2_ to probe the phase behaviour of **1**_**Apohost**_ over a broad *P*/*P*_0_ (partial pressure) range. The **1**_**Apohost**_ to **1ʹ**_**gas**_ transition for CO_2_ occurred at a very low *P*/*P*_0_ (a similar transformation was also observed for N_2_ at 77 K; Supplementary Figs. 15 and 40) and both the CO_2_ and C_2_H_2_ isotherms exhibited additional steps (Supplementary Figs. 16 and 17). Examination of the isotherms using log plots established that the CO_2_ isotherm had three distinct inflections (Fig. 5a), whereas the C_2_H_2_ isotherm had two (Fig. 5c). In the CO_2_ isotherm, adsorption occurred after a step that we attribute to transformation of **1**_**Apohost**_ into **1ʹ**_**CO2**_ with a threshold pressure of ~0.8 mbar (that is, equivalent to that observed at 298 K). A minor step (**1ʹ**_**CO2**_ to **1ʹʹ**_**CO2**_; ~6.7 mbar) and a major step (**1ʹʹ**_**CO2**_ to **1ʹʹʹ**_**CO2**_; ~525 mbar) were also observed. In the case of C_2_H_2_ adsorption, transformation of **1**_**Apohost**_ to **1ʹʹ**_**C2H2**_ occurred at ~0.1 mbar. An additional inflection was observed at ~2.4 mbar, corresponding to a transformation from **1ʹʹ**_**C2H2**_ to **1ʹʹʹ**_**C2H2**_. These multi-step isotherms can be classified as type F-IV^m^^24^.

### In situ PXRD characterization

Concurrent in situ PXRD data (selected patterns are presented in Fig. 5b,d; for additional patterns, see Supplementary Figs. 29–34 and 41) were recorded during the 195 K CO_2_ and 189 K C_2_H_2_ sorption experiments and verified that the bulk-phase pattern of **1**_**Apohost**_ at each temperature was consistent with the calculated PXRD pattern of **1**_**Apohost**_ obtained from the SCXRD data. Upon exposure to 0.8 mbar of CO_2_, **1**_**Apohost**_ underwent a transformation to **1ʹ**_**CO2**_ with a plateau at 3.7 mbar. Compared with **1**_**Apohost**_, the PXRD pattern showed that most peaks had shifted towards a lower 2*θ* (consistent with unit cell expansion), some had disappeared (for example, 2*θ* = 24.7°) and new peaks had appeared (2*θ* = 24.3 and 25.5°). Furthermore, the experimentally obtained pattern at 3.7 mbar matched well with that generated from the SCXRD data (1 bar CO_2_ at 298 K), validating the formation of **1ʹ**_**CO2**_. Progressive CO_2_ gas loading resulted in PXRD peaks shifting to lower 2*θ* positions. At a loading of 32 mbar, additional small peaks were observed at 13.4 and 29.8°. The lack of statistically significant additional peaks suggests that a minor structural change occurred during gas sorption to form **1ʹʹ**_**CO2**_.

Unit cell parameters from the Le Bail profile fitting demonstrated a progressive expansion of all three cell parameters and the unit cell volume (Supplementary Figs. 35–39 and Supplementary Table 4). A comparison of the pattern obtained for **1ʹʹ**_**CO2**_ with that of **1**_**DMF**_ (Supplementary Fig. 32) indicated that the cavities of **1ʹʹ**_**CO2**_ resemble those of **1**_**DMF**_.

Further loading resulted in a large step in the 195 K CO_2_ isotherm at 530 mbar (**1ʹʹʹ**_**CO2**_). The PXRD pattern recorded at 960 mbar revealed the disappearance of two peaks (2*θ* = 15.3 and 16.0°) and the appearance of new peaks at 15.6, 18.0 and 19.3°. Although this phase was unlike the previous phases (Supplementary Fig. 31 and 33), this transformation was reversible, as evidenced by conversion to **1ʹʹ**_**CO2**_ and **1ʹ**_**CO2**_ upon desorption.

In the case of C_2_H_2_, transformation of **1**_**Apohost**_ to **1ʹʹ**_**C2H2**_ (all PXRD peaks shifted to lower 2*θ*) occurred with a plateau at 2.4 mbar (Fig. 5d, yellow). This phase matched the PXRD of **1ʹʹ**_**CO2**_. Additional C_2_H_2_ loading induced peak shifting to lower 2*θ* values, consistent with unit cell expansion and transformation of **1ʹʹ**_**C2H2**_ to **1ʹʹʹ**_**C2H2**_ with saturation at 734 mbar. **1ʹʹʹ**_**CO2**_ and **1ʹʹʹ**_**C2H2**_ are not isostructural. Owing to the increased experimental single-point pore volume and expanded cell parameters (Supplementary Fig. 31 and Supplementary Table 3) for **1ʹʹʹ**_**CO2**_, we consider that the initially discrete cavities merge to form larger voids that accommodate more CO_2_ molecules.

### Structural insight into 1ʹʹ_CO2_

Although the high-pressure CO_2_ 294 K gas sorption isotherm (Supplementary Fig. 25) for **1** only shows the first transition from **1**_**Apohost**_ to **1ʹ**_**CO2**_, we attempted to obtain the **1ʹʹ**_**CO2**_ phase crystallographically by exposing a crystal of **1**_**Apohost**_ to the maximum cylinder pressure attainable for CO_2_ at 298 K (56 bar). After allowing the crystal to equilibrate for 3 h, the environment cell was sealed and SCXRD data were then recorded. As evidenced by the 195 K CO_2_ isotherm and corresponding in situ PXRD, the conversion of **1ʹ**_**CO2**_ to **1ʹʹ**_**CO2**_ occurs with a minor increase in the cell dimensions. The most notable parameter change is the unit cell volume, which increases by 157 Å^3^, resulting in discrete voids of 373 Å^3^ that appear to be expanding towards one another, hinting at the potential nature of guest transport (Fig. 3). As with **1ʹ**_**CO2**_, the CO_2_ guest could not be reliably modelled crystallographically in **1ʹʹ**_**CO2**_. Residual electron density analysis indicated the presence of 1.4 molecules of CO_2_ per ASU. The calculated PXRD pattern generated from the structure of **1ʹʹ**_**CO2**_ is in good agreement with the bulk-phase pattern of **1ʹʹ**_**CO2**_ at 195 K CO_2_ (Supplementary Fig. 34).

### Computational insight into the mechanism of guest transport

**Fig. 6:**
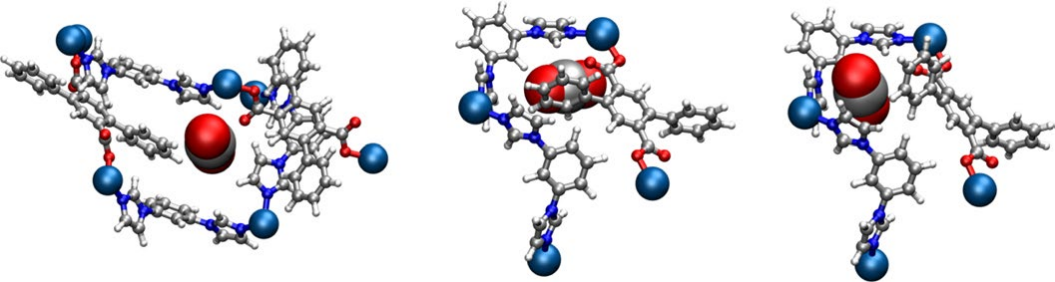
Computationally simulated guest transport in 1. Left, computational model of a single CO_2_ gas molecule at the narrow aperture (lowest-energy pathway), between diagonally adjacent closed cavities, corresponding to the channel traversed in Supplementary Videos 1 and 2. Middle, CO_2_ gas molecule at the dpt linker barrier between stacked discrete cavities, corresponding to the channel traversed in Supplementary Videos 3 and 4. Right, CO_2_ traversing the barrier between stacked cavities, corresponding to a midpoint snapshot of Supplementary Videos 3 and 4, illustrating the diffusive pathway. Atom colours: Co = cobalt, C = grey, O = red, N = blue, H = white. Source data

To gain further insight into the experimentally observed transient diffusion mechanism(s), the spatial relationship between isolated cavities in the two crystal structures, **1ʹ**_**CO2**_ and **1ʹʹ**_**CO2**_, was examined computationally. These experimentally obtained crystal forms have the same structural motifs, suggesting that the mechanism is isomorphic. It was found that each cavity–cavity geometry occurs with two neighbouring orientations; cavities are either diagonally adjacent, as shown in Fig. 3 for **1**_**DMF**_, **1ʹ**_**CO2**_ and **1ʹʹ**_**CO2**_, or are vertically stacked in a single direction (see the far right frame of Fig. 2). This strongly suggests that diffusion within these materials occurs via these pathways. Diagonally adjacent cavities are connected by narrow apertures that are sufficiently constricted to block transport. Guest transport appears unfeasible without the accommodating structural transformations highlighted in Fig. 6. Stacked voids form a corridor divided by dpt linkers (Fig. 6), which section them into discrete cavities.

To probe the viability of transport through these barriers, a rigid **1ʹ**_**CO2**_ structure was loaded with a single CO_2_ molecule near the initially blocked channel. These mono-loaded structures were subjected to relaxation using density functional theory, implemented by the CP2K simulation package, to obtain an optimized initial position^58–78^. The position of the sorbate was then adjusted towards the barrier and a new relaxation was performed, wherein a single sorbate atom had the coordinate in the direction of the barrier held constant, relaxing the crystal to accommodate it. This adjustment was performed until the CO_2_ molecule had traversed the barrier into the adjacent cavity. The trajectory maximum barrier heights were found to represent only a small multiple of ambient thermal energy comparable to the CO_2_ sorption energy. A more detailed description of this methodology is included in the section ‘Computational Modelling’ of the Supplementary Information, where the collective diffusion coordinate is demonstrated explicitly via animations.

In the case of diagonally adjacent cavities, transport was characterized by small-scale shifting and ring rotation of proximal functional groups, which reverted to their initial configurations once they were no longer in the vicinity of the sorbate. The largest change in energy for the inter-cavity transport measured was 4.8 kJ mol^−1^, a barrier substantially lower than the enthalpy of gate opening for CO_2_ (Δ*H*_GO_ = 25.2 kJ mol^−1^) and less than twice the ambient thermal energy at 298 K. For stacked cavities, twisting of the dpt ligand occurs to facilitate transport, involving a rotation of the Co–dpt–Co axis, as well as a benzene pendant ring. Animation of the transport trajectory is included in the section ‘Computational Modelling’ of the Supplementary Information. Notably, the same linker rotates outward during the **1**_**Apohost**_ to **1ʹ**_**CO2**_ transition, causing pairs of small cavities observed in a single unit cell of **1**_**Apohost**_ to merge into the two larger guest-filled cavities observed in a unit cell of **1ʹ**_**CO2**_ (Fig. 3). Consequent to the gross mobility required, a higher energy barrier of 21.9 kJ mol^−1^ (less than nine times the ambient thermal energy) was observed. This barrier is of a higher energy than the diagonally adjacent cavity transport that is likely to be the most favourable diffusion mechanism. To probe the dynamic motion between cavities, a single cavity in an otherwise vacant **1ʹ**_**CO2**_ crystal structure was loaded with 12 CO_2_ molecules via Grand Canonical Monte Carlo simulation and subjected to density functional molecular dynamics (DFTMD) simulations in the canonical ensemble at 473 and 623 K. Less than 1 ps at both temperatures produced thermal diffusion between diagonally adjacent cavities in a manner consistent with the more energetically favourable pathway described above. Animations of these molecular trajectories are provided as Supplementary Videos 1–6.

## Conclusion

**1**_**Apohost**_ exhibits **dmp** topology with zero-dimensional spherical cavities and is unlike previous switching FMOMs, most of which feature gas-induced transformations requiring extreme network contortion^9^ or displacement^9,12^. Specifically, the type F-IV^m^ sorption behaviour exhibited by **1**_**Apohost**_ arises from transient porosity that enables gas-induced intra-network distortion and transformations to phases with higher gas uptakes. We have shown an example of type F-IV adsorption behaviour in a seemingly non-porous coordination network that is a clathrate in each of four structurally characterized phases. These gas-induced transformations combine recyclability, optimal working capacity and high uptake without the need for extreme structural transformations. Sorbate diffusion between isolated cavities can take place along two discrete potential vectors, with travel through diagonally adjacent cells as the likely dominant mechanism. Small-scale collective molecular motions and ring rotation create a path through an otherwise inaccessible cavity. The second transport vector involves rotation of the dpt linker, with the more well-defined mechanism lending itself well to potential tuning of gate opening in future analogues. Furthermore, as the motion observed in this material arises from cooperative movement ascribed to both ligands, crystal engineering design principles could be used to iteratively expand the cavity and, in turn, the loading capacity while preserving the phenomenon of transient porosity.

## Methods

### Materials

Commercially available starting materials and solvents were purchased from Sigma–Aldrich, Merck and Fluorochem. All reactions were monitored using aluminium-backed silica gel Merck 60 F_254_ TLC plates and visualized using ultraviolet irradiation. All chemicals were used as received without additional purification.

### Synthesis of 1,3-bib

The synthesis of 1,3-bib was carried out in a single step, according to a previously reported procedure with minor modifications^79^. 1,3-dibromobenzene (10.0 g; 42.8 mol; 1.0 equiv.), imidazole (14.5 g; 213.0 mmol; 5.0 equiv.), CuI (1.63 g; 8.6 mmol; 20 mol%) and K_2_CO_3_ (29.4 g; 213.0 mmol; 5.0 equiv.) were all added to anhydrous DMF (150 ml) under an inert N_2_ atmosphere. The resulting reaction mixture was heated to reflux under an inert atmosphere for 72 h. After cooling to room temperature, the mixture was filtered. The filtered residue was washed with dichloromethane (2 × 150 ml) and the filtrate was transferred to a large separating funnel. The organic layer was washed with water (3 × 250 ml), separated and dried over MgSO_4_. The organic layer was concentrated under reduced pressure and the resulting solid material was finally purified by trituration from a dichloromethane/hexane mixture. The resulting 1,3-bib was isolated as a white solid (8.41 g; 94%).

### Synthesis of dpt

The synthesis of dpt was carried out in two steps, according to a previously reported procedure^80^. In step 1, 20 ml deionized water was degassed for 30 min using N_2_. 2,5-dibromo-*p*-xylene (2.00 g; 7.58 mmol; 1.0 equiv.), phenyl boronic acid (2.03 g; 16.7 mmol; 2.2 equiv.), Pd(OAc)_2_ (3.40 mg; 0.2 mol%), K_2_CO_3_ (5.24 g; 37.9 mmol; 5.0 equiv.) and *n*-Bu_4_NBr (4.87 g; 15.2 mmol; 2.0 equiv.) were all added. The resulting suspension was heated to 343 K for 4 h and stirred vigorously under an inert atmosphere. After cooling to room temperature, the reaction mixture was diluted with water (150 ml) and extracted with hexane. The organic phase was dried over MgSO_4_ and concentrated under reduced pressure to yield 2′,5′-dimethyl-*p*-terphenyl as a white solid (1.88 g; 96%). In step 2, 2′,5′-dimethyl-*p*-terphenyl (700 mg; 2.71 mmol; 1.0 equiv.) was added to 20 ml pyridine. Then, 2.2 g KMnO_4_ in 2.0 ml H_2_O was added and the reaction mixture was heated to reflux for 2 h. After reaching reflux, every 30 min, an additional 1.0 g KMnO_4_ in 2.0 ml H_2_O was added (a total of four times). After 6 h at reflux, a final 10 ml water was added to the reaction mixture, which was allowed to reflux overnight. The MnO_2_ precipitate was hot filtered from the reaction mixture and washed with near-boiling water (100 ml). The filtrate was carefully acidified using concentrated HCl, precipitating the dpt product as a white solid, which was collected by filtration, washed with 0.2 M HCl and finally dried in a 378 K oven overnight (732 mg; 85%).

### Crystallization

Crystals of **1**_**DMF**_ were grown solvothermally by combining 0.3 mmol (63.0 mg) dpt, 0.3 mmol (95.0 mg) 1,3-bib and 0.3 mmol (87.0 mg) cobalt nitrate hexahydrate in 10 ml DMF and heating at 393 K. Purple block crystals were obtained after 2 d.

### SCXRD

SCXRD data were recorded on a Bruker SMART APEX II^81^ and a Bruker QUEST APEX III equipped with an Mo or Cu sealed tube source. Both diffractometers employ an Oxford Cryosystems 700 Plus cryostat to control the temperature of the sample. Data reduction was carried out by means of standard procedures using the Bruker software package SAINT^82^. Absorption corrections and correction of other systematic errors were carried out using SADABS^83^. All structures were solved by direct methods using SHELXS-16 and refined using SHELXL-16 (ref. ^84^). X-Seed^85^ was used as the graphical interface for the SHELX program suite. Solvent-accessible voids can be visualized by calculating Connolly surfaces using MS-ROLL^51,52^, another program incorporated into X-Seed. Hydrogen atoms were placed in calculated positions using riding models.

### Activation procedure

A suitable crystal of the as-synthesized material was selected and glued onto a glass fibre with cyanoacrylate glue. The glass fibre was then inserted into an environmental gas cell (EGC), which consisted of a 0.3 mm Lindemann capillary secured to a steel nut with epoxy that was screwed into a valve body. The EGC allows for evacuation/pressurization of the immediate crystal environment and transportation to a diffractometer. The EGC was then connected to a Pfeiffer HiCube vacuum pump (pressure = ~3 × 10^−3^ mbar) and immersed in oil, which was heated to 393 K overnight. The valve was then closed and the EGC was removed from the activation apparatus. The evacuated crystal in an EGC was mounted onto a conventional goniometer and SCXRD data were recorded at 298 K.

### Gas-loading experiments

The activated crystal in the EGC was attached to a CO_2_ cylinder via a gas manifold (regulator). The system was pressurized to 1 bar and left to equilibrate under static pressure for 3 h (multiple equilibration times were tested and this was found to be the best), after which the EGC was closed and loaded onto the diffractometer.

### PXRD

PXRD experiments were conducted using microcrystalline samples on a PANalytical Empyrean diffractometer (40 kV; 40 mA; CuKα_1,2_ *λ* = 1.5418 Å) in Bragg–Brentano geometry. A scan speed of 0.044509° s^−1^ (2.6° min^−1^), with a step size of 0.0262° in 2*θ*, was used at room temperature with a range of 5° < 2*θ* < 40°. Powder samples were evenly distributed on a zero-background holder after being ground with a mortar and pestle to minimize the effects of preferred orientation. Data analysis was carried out using X’Pert HighScore Plus^86^ (version 2.2e). Powder patterns were simulated from SCXRD structures using Mercury^87^.

### Thermogravimetric analysis

Thermogravimetric analysis was performed under N_2_ using a TA Instruments Q50 system. A sample was loaded into an aluminium sample pan and heated at 283 K min^−1^ from room temperature to 773 K.

### Differential scanning calorimetry

Differential scanning calorimetry was carried out using a TA Instruments Q2000 differential scanning calorimeter. Samples were prepared by crimping the sample pan and lid (a pin hole was placed in the lid to prevent pressure build-up). A reference pan was prepared in the same manner for each analysis. Analyses were generally carried out in the temperature range 253–523 K and a general experimental procedure consisted of two heating/cooling cycles while the heat flow into or out of the sample, relative to the reference, was measured as a function of time and temperature under a controlled atmosphere. N_2_ gas flowing at a rate of 50 ml min^−1^ was used to purge the furnace. The resulting thermograms were analysed using TA Instruments’ Universal Analysis program and Supplementary Fig. 14 was prepared with Microsoft Excel.

### Gas sorption isotherm measurements

Before performing the gas sorption experiments, a freshly prepared sample of **1**_**DMF**_ was placed in a quartz tube and degassed under high vacuum using a Smart VacPrep instrument at 343 K for 24 h to remove any remaining solvent molecules and yield **1**_**Apohost**._ Isotherms were measured using a Micromeritics 3Flex sorption analyser. Gases were used as obtained from BOC Gases (Ireland), with the following certified purities: research-grade He (99.999%), CO_2_ (99.995%), C_2_H_2_ (98.5%), C_2_H_4_ (99.92%), C_2_H_6_ (99.0%) and N_2_ (99.998%). Bath temperatures of 77 and 195 K were maintained using liquid nitrogen and a dry ice–acetone slurry, respectively. A Julabo ME version 2 temperature controller was used to maintain bath temperatures in the 273 and 298 K experiments. Samples were activated between successive experiments overnight or for a minimum of 5 h at 343 K under high vacuum. High-pressure CO_2_ sorption experiments were performed using a Hiden Isochema XEMIS microbalance. Activated samples of **1**_**Apohost**_ were further outgassed under secondary vacuum for 3 h in situ before isotherms were run. Excess adsorption and desorption profiles were obtained after applying buoyancy correction using the crystallographically determined density of **1**_**Apohost**_. Temperatures were maintained at 273 and 294 K using a Grant LT Ecocool 150 temperature controller. Equilibration was determined by a cut-off criterion of agreement within 0.01% of a pressure reading, with rolling averages of the previous ten pressure readings each collected after allowing the equilibration interval (10 s for the 195 K CO_2_ experiments and 77 K N_2_ experiments and 30 s for the 273 and 298 K experiments) to elapse. Equilibration data were recorded for the full range of pressure dosing during the 298 K CO_2_ and C_2_H_2_ experiments, with a sampling interval of around two pressure readings per second.

### Single-point pore volumes

Indicative single-point pore volumes were determined experimentally using the MicroActive software suite under the assumption of approximate validity of the Gurvich rule^88,89^, wherein the structural transformations follow a state approaching saturation of the preceding phase during 195 K CO_2_ adsorption experiments, as described in equation (1), where *ν*_pore_. is the pore volume, $n_{{\rm{CO}}_{2}}^{{\rm{Ads}}}$ is the quantity of gaseous CO_2_ adsorbed, as determined at standard temperature and pressure, and $\rho _{{\rm{CO}}_{2}}^{{\rm{Liq}}}$ Is the density of liquid CO_2_.

1 $$\nu _{{{{\mathrm{pore}}}}} = \frac{{n_{{\rm{CO}}_{2}}^{{\rm{Ads}}}}}{{\rho _{{\rm{CO}}_{2}}^{{\rm{Liq}}}}}$$

The determined volumes are presented with 5% error bars (Supplementary Table 3) and show reasonable agreement with the assignment of phases based on in situ PXRD, as well as SCXRD-determined solvent-accessible volumes.

### Molar enthalpy of gate opening

The molar enthalpy of gate opening (Δ*H*_GO_) was calculated from the gate-opening pressures (*P*_GO_) determined from pure component isotherms at 273 and 298 K, using the Clausius–Clapeyron equation (R = the gas constant, T = absolute temperature) as described in equation (2). Enthalpies were found to be correlated with molar enthalpies of vaporization, in agreement with Li et al.^55^. Error bars represent 5% margins in Supplementary Fig. 27.

2 $${\Delta} H_{{\rm{GO}}} = {\rm{RT}}^2\left( {\frac{{\partial \ln P_{{\rm{GO}}}}}{{\partial T}}} \right)$$

### In situ PXRD

In situ coincident PXRD measurements were conducted on a Rigaku SmartLab with CuKα radiation, which was synchronized to a BELSORP-18PLUS volumetric adsorption instrument (MicrotracBEL). A helium-based cryosystem was connected to the sorption equipment to control the temperature range. The as-synthesized sample was soaked in MeOH for 3 d and then activated at 343 K under vacuum overnight using a copper plate holder. The activated sample of ~70 mg was transferred to the sorption instrument and treated again under vacuum at 353 K for 2 h. The second activation was performed to remove any adsorbed moisture during transfer. This is an essential step as the sample adsorbs water from the atmosphere. A CO_2_ sorption experiment was carried out up to 100 kPa at 195 K. In situ PXRD patterns were measured simultaneously at each equilibrium point of the adsorption and desorption isotherm. In addition to CO_2_, in situ PXRD patterns were measured at each equilibrium point of the adsorption isotherm for N_2_ at 77 K and C_2_H_2_ at 189 K. Powder patterns obtained from the in situ variable pressure PXRD experiment of **1**_**Apohost**_ at different CO_2_ adsorption loadings were indexed using the program EXPO2014 (refs. ^90,91^).

### Computational modelling

Computational modelling of the **1** crystal phases using electronic structure methods was undertaken via density function theory (DFT). Crystal structures were treated to full periodic relaxation using MOLOPT basis sets at the triple zeta level of theory and Perdew–Burke–Ernzerhof pseudopotentials. Dispersion was treated using a pair potential with the DFT-D3 correction of Grimme et al. These were implemented using the CP2K simulation package^58–69,77,78^. DFTMD simulations were implemented similarly using the canonical ensemble with a fixed particle number, volume and temperature (NVT) with a 0.5 fs timestep. The chosen thermostat was Nosé–Hoover, using a time constant of 100 fs. Classical modelling of CO_2_ sorption in **1** was undertaken to examine the sorptive behaviour and uptakes in these structures. These simulations involved Monte Carlo simulation of sorbates within a rigid crystal scaffold, as employed by the massively parallel Monte Carlo code^92^. Intermolecular forces were parametrized using the Universal Force Field^93^, the extended charge equilibration method^94^ and Thole–Applequist-type point polarizabilities^95,96^ taken from the work of van Duijnen and Swart^97^ to model the van der Waals, electrostatic and induced dipole effects, respectively. CO_2_-PHAST*^98^ was used for the sorbate parameters. X-ray crystal structures of **1**_**Apohost**_, **1ʹ**_**CO2**_ and **1ʹʹ**_**CO2**_ were relaxed using DFT, as described above. For all three, there was only minor alteration in atomic positions after optimization, providing stable optimized conformations even for the open **1ʹ**_**CO2**_ and **1ʹʹ**_**CO2**_ structures. Classical simulation in the relaxed structures confirmed that they retained porosity, loaded sorbate into discrete pockets and supported CO_2_ uptakes equal to, or in excess of, the loadings reached in each structure to induce the next open phase as reported.

The potential for transient transport between neighbouring isolated cavities was examined along two potential pathways in the **1ʹ**_**CO2**_ structure. An initial position was obtained from canonical Monte Carlo runs of CO_2_ sorption. A single sorbate position settled near the posited transport window was selected from each of the trajectories and settled into the local minima via simulated annealing. A single unit cell was then taken to probe transport between diagonally adjacent voids as each **1ʹ**_**CO2**_ unit cell contains two cavities with that relative orientation. For the stacked cavities, a unit cell was replicated along the *c* axis to create a two-unit cell system with a single sorbate molecule (two cells were used as the stacked relative orientation is not observed within a single unit cell). These systems were then fully relaxed using CP2K. Using these relaxed initial positions, a series of sequential relaxations were performed wherein the optimized structure was modified, shifting the sorbate in the direction of the postulated transport channel, and re-relaxed subject to the constraint that a single sorbate oxygen atom was constrained in one direction (the *b* axis for the diagonally adjacent cavity and the *a* axis for the stacked cavity). This forced proximity to the channel while allowing the atom to shift in the remaining two dimensions to find the optimal route through. No further constraints were put on the other sorbate atoms or the crystal, permitting sorbate orientation and geometry to relax along with the structure. The constrained optimized structures were then taken and the CO_2_ was shifted further along the channel and re-relaxed. These iterations were repeated until a trajectory traversing each channel was obtained. Having determined the pathways, the energies of each sequential configuration were then compared with the initial position energy to determine the energy barrier as reported in the ‘Computational insight into the mechanism of guest transport’ section of the main text.

Dynamic molecular motion between cavities in the **1ʹ**_**CO2**_ structure was modelled using a single unit cell with one cavity loaded with 12 CO_2_ molecules and the second cavity left empty. Initial positions of sorbate occupants were taken from grand canonical Monte Carlo simulation. The single-cavity-loaded structure was subjected to DFTMD simulations allowing all atomic positions to fluctuate at 473 and 623 K. The sorbate loading differential between cavities was found to provide sufficient motive impetus for sorbate molecules to diffuse through the barrier between diagonally adjacent cavities. Simulation across both temperatures reproduced the more energetically favourable mechanism of diffusion via rotation of the dpt linker.

## Online content

Any methods, additional references, Nature Portfolio reporting summaries, source data, extended data, supplementary information, acknowledgements, peer review information; details of author contributions and competing interests; and statements of data and code availability are available at 10.1038/s41557-022-01128-3.

## Supplementary information

Supplementary Information Supplementary Figs. 1–42, Tables 1–5, methods and data.

Supplementary Data 1 Crystallographic data for **1**_**DMF**_ at 100 K (CCDC reference 2111569).

Supplementary Data 2 Crystallographic data for **1**_**DMF**_ at 298 K (CCDC reference 2111570).

Supplementary Data 3 Crystallographic data for **1**_**Hydrate**_ (CCDC reference 2111571).

Supplementary Data 4 Crystallographic data for **1**_**Apohost**_ (CCDC reference 2111572).

Supplementary Data 5 Crystallographic data for **1′**_**CO2**_, (CCDC reference 2111573).

Supplementary Data 6 Crystallographic data for **1′′**_**CO2**_ (CCDC reference 2166387).

Supplementary Data 7 Data used to generate the plot for Supplementary Fig. 39.

Supplementary Video 1 Animation 1. CO_2_ trajectory moving between diagonally adjacent cavities in a supercell. Atom colours: Co = cobalt, C = grey, O = red, N = blue, H = white.

Supplementary Video 2 Animation 2. CO_2_ trajectory moving between diagonally adjacent cavities showing only proximal functionality. Atom colours: Co = cobalt, C = grey, O = red, N = blue, H = white.

Supplementary Video 3 Animation 3. CO_2_ trajectory moving between stacked cavities in a supercell. Atom colours: Co = cobalt, C = grey, O = red, N = blue, H = white.

Supplementary Video 4 Animation 4. CO_2_ trajectory moving between stacked cavities showing only proximal functionality. Atom colours: Co = cobalt, C = grey, O = red, N = blue, H = white.

Supplementary Video 5 Animation 5. Molecular dynamics trajectory of a CO_2_ molecule in the **1ʹ**_**CO2**_ structure with a single cavity loaded with 12 sorbate molecules. Dynamic motions include migration from the loaded cavity to the unoccupied diagonally adjacent cavity at 473 K. Atom colours: Co = ice blue, C = grey, O = red, N = blue, H = white.

Supplementary Video 6 Animation 6. Molecular dynamics trajectory of a CO_2_ molecule in the **1ʹ**_**CO2**_ structure with a single pore loaded with 12 sorbate molecules. Dynamic motions include migration from the loaded cavity to the unoccupied diagonally adjacent cavity at 623 K. Atom colours: Co = ice blue, C = grey, O = red, N = blue, H = white.

## Data Availability

All of the relevant data that support the findings of this research are available within the Article and its Supplementary Information files. Crystallographic data for the structures reported in this Article have been deposited at the Cambridge Crystallographic Data Centre under deposition numbers CCDC 2111569 (**1**_**DMF**_; 100 K), 2111570 (**1**_**DMF**_; 298 K), 2111571 (**1**_**Hydrate**_), 2111572 (**1**_**Apohost**_), 2111573 (**1′′**_**CO2**_) and 2166387 (**1′**_**CO2**_). Copies of the data can be obtained free of charge via https://www.ccdc.cam.ac.uk/structures/. In addition, all of the experimental datasets are available from the online Zenodo repository (10.5281/zenodo.7118299)^99^. Source data are provided with this paper.

## Source data

Source Data Fig. 3 CIF files for the single-crystal data used to create the graphics for Fig. 3.
Source Data Fig. 4 Sorption data collected at 298 K for CO_2_, C_2_H_2_C, C_2_H_4_ and C_2_H_6_ and used to create the plots.
Source Data Fig. 5 In situ PXRD and sorption data collected for CO_2_ and C_2_H_2_ and used to create plots.
Source Data Fig. 6 CIF file for the computation model used to create the graphics in Fig. 6.

## References

[1] Perry JJ, Perman JA, Zaworotko MJ (2009). Design and synthesis of metal–organic frameworks using metal–organic polyhedra as supermolecular building blocks. Chem. Soc. Rev..

[2] Chen Z (2021). The state of the field: from inception to commercialization of metal–organic frameworks. Faraday Discuss..

[3] Furukawa H, Cordova KE, O’Keeffe M, Yaghi OM (2013). The chemistry and applications of metal–organic frameworks. Science.

[4] Farrusseng, D. *Metal*–*Organic Frameworks: Applications from Catalysis to Gas Storage* (Wiley, 2011).

[5] Kitagawa S, Kitaura R, Noro S-I (2004). Functional porous coordination polymers. Angew. Chem. Int. Ed..

[6] Batten, S. R., Neville, S. M. & Turner, D. R. *Coordination Polymers: Design, Analysis and Application* (Royal Society of Chemistry, 2009).

[7] Horike S, Shimomura S, Kitagawa S (2009). Soft, porous crystals. Nat. Chem..

[8] Simon CM (2015). The materials genome in action: identifying the performance limits for methane storage. Energy Environ. Sci..

[9] Schneemann A (2014). Flexible metal–organic frameworks. Chem. Soc. Rev..

[10] Férey G (2008). Hybrid porous solids: past, present, future. Chem. Soc. Rev..

[11] Murdock CR, Hughes BC, Lu Z, Jenkins DM (2014). Approaches for synthesizing breathing MOFs by exploiting dimensional rigidity.. Coord. Chem. Rev..

[12] Krause S, Hosono N, Kitagawa S (2020). Chemistry of soft porous crystals: structural dynamics and gas adsorption properties. Angew. Chem. Int. Ed..

[13] Sakaida S (2016). Crystalline coordination framework endowed with dynamic gate-opening behaviour by being downsized to a thin film. Nat. Chem..

[14] Jin J, Zhao X, Feng P, Bu X (2018). A cooperative pillar–template strategy as a generalized synthetic method for flexible homochiral porous frameworks. Angew. Chem..

[15] Engel ER, Jouaiti A, Bezuidenhout CX, Hosseini MW, Barbour LJ (2017). Activation-dependent breathing in a flexible metal–organic framework and the effects of repeated sorption/desorption cycling. Angew. Chem. Int. Ed..

[16] Hazra A (2019). CO_2_-induced single-crystal to single-crystal transformations of an interpenetrated flexible MOF explained by in situ crystallographic analysis and molecular modeling. Chem. Sci..

[17] Wharmby MT (2015). Extreme flexibility in a zeolitic imidazolate framework: porous to dense phase transition in desolvated ZIF-4. Angew. Chem. Int. Ed..

[18] McGuirk CM (2018). Influence of metal substitution on the pressure-induced phase change in flexible zeolitic imidazolate frameworks. J. Am. Chem. Soc..

[19] Kundu T, Wahiduzzaman M, Shah BB, Maurin G, Zhao D (2019). Solvent induced control over breathing behavior in flexible metal–organic frameworks for natural-gas delivery. Angew. Chem. Int. Ed..

[20] Yang H (2018). Visualizing structural transformation and guest binding in a flexible metal–organic framework under high pressure and room temperature. ACS Cent. Sci..

[21] Li D, Kaneko K (2001). Hydrogen bond-regulated microporous nature of copper complex-assembled microcrystals. Chem. Phys. Lett..

[22] Seki K (2002). Dynamic channels of a porous coordination polymer responding to external stimuli. Phys. Chem. Chem. Phys..

[23] Kitaura R, Fujimoto K, Noro S-I, Kondo M, Kitagawa S (2002). A pillared-layer coordination polymer network displaying hysteretic sorption: [Cu_2_(pzdc)_2_(dpyg)]_*n*_ (pzdc = pyrazine-2,3-dicarboxylate; dpyg = 1,2-di(4-pyridyl)-glycol). Angew. Chem. Int. Ed..

[24] Wang S-Q, Mukherjee S, Zaworotko MJ (2021). Spiers Memorial Lecture: coordination networks that switch between nonporous and porous structures: an emerging class of soft porous crystals. Faraday Discuss..

[25] Maji TK, Mostafa G, Matsuda R, Kitagawa S (2005). Guest-induced asymmetry in a metal–organic porous solid with reversible single-crystal-to-single-crystal structural transformation. J. Am. Chem. Soc..

[26] Nijem N (2012). Tuning the gate opening pressure of metal–organic frameworks (MOFs) for the selective separation of hydrocarbons. J. Am. Chem. Soc..

[27] Chen Q (2013). A controllable gate effect in cobalt(ii) organic frameworks by reversible structure transformations. Angew. Chem. Int. Ed..

[28] Ichikawa M (2016). Double-step gate phenomenon in CO_2_ sorption of an elastic layer structured MOF. Langmuir.

[29] Yang Q-Y (2018). Reversible switching between highly porous and nonporous phases of an interpenetrated diamondoid coordination network that exhibits gate-opening at methane storage pressures. Angew. Chem. Int. Ed..

[30] Wang S-Q (2018). Recyclable switching between nonporous and porous phases of a square lattice (sql) topology coordination network. Chem. Commun..

[31] Wang S-Q (2019). Highly selective, high-capacity separation of *o*-xylene from C_8_ aromatics by a switching adsorbent layered material. Angew. Chem. Int. Ed..

[32] Zhu A-X (2019). Tuning the gate-opening pressure in a switching pcu coordination network, X-pcu-5-Zn, by pillar-ligand substitution. Angew. Chem. Int. Ed..

[33] Song B-Q (2020). Reversible switching between nonporous and porous phases of a new SIFSIX coordination network induced by a flexible linker ligand. J. Am. Chem. Soc..

[34] Wang S-Q (2021). High working capacity acetylene storage at ambient temperature enabled by a switching adsorbent layered material. ACS Appl. Mater. Interfaces.

[35] Sing KSW (1985). Reporting physisorption data for gas/solid systems with special reference to the determination of surface area and porosity. Pure Appl. Chem..

[36] Engel EE, Jouaiti A, Bezuidenhout CX, Hosseini MW, Barbour LJ (2017). Activation-dependent breathing in a flexible metal–organic framework and the effects of repeated sorption/desorption cycling. Angew. Chem. Int. Ed..

[37] Atwood JL, Barbour LJ, Jerga A, Schottel BL (2002). Guest transport in a nonporous organic solid via dynamic van der Waals cooperativity. Science.

[38] Barbour LJ (2006). Crystal porosity and the burden of proof.. Chem. Commun..

[39] Atwood JL, Barbour LJ, Jerga A (2004). A new type of material for the recovery of hydrogen from gas mixtures. Angew. Chem. Int. Ed..

[40] Riddle JA, Bollinger JC, Lee D (2005). Escape from a nonporous solid: mechanically coupled biconcave molecules. Angew. Chem. Int. Ed..

[41] Thallapally PK, Wirsig TB, Barbour LJ, Atwood JL (2005). Crystal engineering of nonporous organic solids for methane sorption. Chem. Commun..

[42] Thallapally PK (2008). Gas-induced transformation and expansion of a non-porous organic solid. Nat. Mater..

[43] Jones JT (2011). On–off porosity switching in a molecular organic solid. Angew. Chem. Int. Ed..

[44] Holden D (2016). Understanding static, dynamic and cooperative porosity in molecular materials. Chem. Sci..

[45] Brekalo I (2020). Microporosity of a guanidinium organodisulfonate hydrogen‐bonded framework. Angew. Chem. Int. Ed..

[46] Bojdys MJ (2011). Supramolecular engineering of intrinsic and extrinsic porosity in covalent organic cages. J. Am. Chem. Soc..

[47] Tanaka D (2008). Kinetic gate-opening process in a flexible porous coordination polymer. Angew. Chem. Int. Ed..

[48] Sato H (2014). Self-accelerating CO sorption in a soft nanoporous crystal. Science.

[49] Gu C (2019). Design and control of gas diffusion process in a nanoporous soft crystal. Science.

[50] Van Heerden DP, Smith VJ, Aggarwal H, Barbour LJ (2021). High pressure in situ single-crystal X-ray diffraction reveals turnstile linker rotation upon room-temperature stepped uptake of alkanes. Angew. Chem. Int. Ed..

[51] Connolly ML (1983). Solvent-accessible surfaces of proteins and nucleic acids. Science.

[52] Connolly ML (1993). The molecular surface package. J. Mol. Graphics.

[53] Seo J, Bonneau C, Matsuda R, Takata M, Kitagawa S (2011). Soft secondary building unit: dynamic bond rearrangement on multinuclear core of porous coordination polymers in gas media. J. Am. Chem. Soc..

[54] Sen S (2017). Cooperative bond scission in a soft porous crystal enables discriminatory gate opening for ethylene over ethane. J. Am. Chem. Soc..

[55] Li L (2016). Exploiting the gate opening effect in a flexible MOF for selective adsorption of propyne from C_1_/C_2_/C_3_ hydrocarbons. J. Mater. Chem. A.

[56] Spek AL (2008). Structure validation in chemical crystallography. Acta Crystallogr. Sect. D.

[57] Spek AL (2015). PLATON SQUEEZE: a tool for the calculation of the disordered solvent contribution to the calculated structure factors. Acta Crystallogr. Sect. C.

[58] Kuehne TD (2020). CP2K: an electronic structure and molecular dynamics software package—Quickstep: efficient and accurate electronic structure calculations. Chem. Phys..

[59] Goerigk L (2017). A look at the density functional theory zoo with the advanced GMTKN55 database for general main group thermochemistry, kinetics and noncovalent interactions. Chem. Phys..

[60] Schuett, O., Messmer, P., Hutter, J. & VandeVondele, J. in *Electronic Structure Calculations on Graphics Processing Units: From Quantum Chemistry to Condensed Matter Physics* 173–190 (John Wiley & Sons, 2016).

[61] Borstnik U, VandeVondele J, Weber V, Hutter J (2014). Sparse matrix multiplication: the distributed block-compressed sparse row library. Parallel Comput..

[62] Hutter J, Iannuzzi M, Schiffmann F, VandeVondele J (2014). CP2K: atomistic simulations of condensed matter systems. Wiley Interdiscip. Rev. Comput. Mol. Sci..

[63] Marek A (2014). The ELPA library: scalable parallel eigenvalue solutions for electronic structure theory and computational science. J. Phys. Condens. Matter.

[64] Grimme S, Ehrlich S, Goerigk L (2011). Effect of the damping function in dispersion corrected density functional theory. J. Comput. Chem..

[65] Grimme S, Antony J, Ehrlich S, Krieg H (2010). A consistent and accurate ab initio parametrization of density functional dispersion correction (DFT-D) for the 94 elements H–Pu. Chem. Phys..

[66] VandeVondele J, Hutter J (2007). Gaussian basis sets for accurate calculations on molecular systems in gas and condensed phases. Chem. Phys..

[67] Kuhne TD, Krack M, Mohamed FR, Parrinello M (2007). Efficient and accurate Car–Parrinello-like approach to Born–Oppenheimer molecular dynamics. Phys. Rev. Lett..

[68] Krack M (2005). Pseudopotentials for H to Kr optimized for gradient-corrected exchange-correlation functionals. Theor. Chem. Acc..

[69] VandeVondele J (2005). QUICKSTEP: fast and accurate density functional calculations using a mixed Gaussian and plane waves approach. Comput. Phys. Commun..

[70] Frigo M, Johnson SG (2005). The design and implementation of FFTW3. Proc. IEEE.

[71] Kolafa J (2004). Time-reversible always stable predictor–corrector method for molecular dynamics of polarizable molecules. J. Comput. Chem..

[72] VandeVondele J, Hutter J (2003). An efficient orbital transformation method for electronic structure calculations. Chem. Phys..

[73] Hartwigsen C, Goedecker S (1998). Hutter, relativistic separable dual-space Gaussian pseudopotentials from H to Rn. Phys. Rev. B.

[74] Lippert G, Hutter J, Parrinello M (1997). A hybrid Gaussian and plane wave density functional scheme. Mol. Phys..

[75] Perdew JP, Burke K, Ernzerhof M (1996). Generalized gradient approximation made simple. Phys. Rev. Lett..

[76] Goedecker S, Teter M, Hutter J (1996). Separable dual-space Gaussian pseudopotentials. Phys. Rev. B.

[77] Nose S (1984). A unified formulation of the constant temperature molecular-dynamics methods. J. Chem. Phys..

[78] Nose S (1984). A molecular dynamics method for simulations in the canonical ensemble. Mol. Phys..

[79] Collado A (2016). Synthesis of Au^I^‐ and Au^III^‐bis(NHC) complexes: ligand influence on oxidative addition to Au^I^ species. Eur. J. Inorg. Chem..

[80] Merlet S, Birau M, Wang ZY (2002). Synthesis and characterization of highly fluorescent indenofluorenes. Org. Lett..

[81] SMART Data Collection Software v.5.629 (Bruker AXS, 2003).

[82] SAINT Data Reduction Software v.6.45 (Bruker AXS, 2003).

[83] SADABS v2.03 (Bruker AXS, 2014).

[84] Sheldrick GM (2015). Crystal structure refinement with SHELXL. Acta Crystallogr. Sect. C.

[85] Barbour LJ (2001). X-Seed—a software tool for supramolecular crystallography. J. Supramol. Chem..

[86] Degen T, Sadki M, Bron E, König U, Nénert G (2014). The HighScore suite.. Powder Diffr..

[87] Macrae CF (2008). Mercury CSD 2.0—new features for the visualization and investigation of crystal structures. J. Appl. Crystallogr..

[88] Gurvich L (1915). J. Phys. Chem. Soc. Russ..

[89] Islamoglu T (2022). Are you using the right probe molecules for assessing the textural properties of metal–organic frameworks?. J. Mater. Chem. A.

[90] Altomare A (2013). EXPO2013: a kit of tools for phasing crystal structures from powder data. J. Appl. Cryst..

[91] Altomare A, Camalli M, Cuocci C, Giacovazzo C, Moliterni A, Rizzi R (2009). EXPO2009: structure solution by powder data in direct and reciprocal space. J. Appl. Cryst..

[92] Belof, J. L. & Space, B. Massively Parallel Monte Carlo (MPMC). *GitHub* https://github.com/mpmccode/mpmc (2023).

[93] Rappe AK, Casewit CJ, Colwell KS, Goddard WA, Skiff WM (1992). UFF, a full periodic table force field for molecular mechanics and molecular dynamics simulations. J. Am. Chem. Soc..

[94] Wilmer CE, Kim KC, Snurr RQ (2012). An extended charge equilibration method. J. Phys. Chem..

[95] Thole BT (1981). Molecular polarizabilities calculated with a modified dipole interaction. Chem. Phys..

[96] Applequist J, James RC, Fung K-K (1972). Atom dipole interaction model for molecular polarizability. Application to polyatomic molecules and determination of atom polarizabilities. J. Am. Chem. Soc..

[97] Van Duijnen PT, Swart M (1998). Molecular and atomic polarizabilities: Thole’s model revisited. Am. J. Phys. Chem..

[98] Mullen AL (2013). A polarizable and transferable PHAST CO_2_ potential for materials simulation. J. Chem. Theory Comput..

[99] Nikolayenko, V. I. et al. Reversible transformations between the non-porous phases of a flexible coordination network enabled by transient porosity. *Zenodo*10.5281/zenodo.7118299 (2023). 10.1038/s41557-022-01128-3 PMC10070188 36781909

