# Supplementary material for: Reversible transformations between the non-porous phases of a flexible coordination network enabled by transient porosity
Source: Nat Chem. 2023 Feb 13;15(4):542–9. doi: 10.1038/s41557-022-01128-3 (PMC10070188; doi:10.1038/s41557-022-01128-3)
Supplement: Supplementary file 1 — Supplementary Figs. 1–42, Tables 1–5, methods and data. [file 41557_2022_1128_MOESM1_ESM.pdf]

nature chemistry

Article

https://doi.org/10.1038/s41557-022-01128-3

Reversible transformations between the non-porous phases of a flexible coordination network enabled by transient porosity

In the format provided by the authors and unedited

Electronic Supporting Information

Reversible transformations between the non-porous phases of a flexible coordination network enabled by transient porosity.

Varvara I. Nikolayenko[a], Dominic C. Castell[a], Debobroto Sensharma[a], Mohana Shivanna[b], Leigh Loots[c], Katherine A. Forrest[d], Carlos J. Solanilla-Salinas[e], Ken-ichi Otake[b], Susumu Kitagawa[b], Leonard J. Barbour[c], Brian. Space[d,e] and Michael J. Zaworotko[a*]

[a] Department of Chemical Sciences and Bernal Institute, University of Limerick, Limerick V94 T9PX, Republic of Ireland.

[b] Institute for Integrated Cell-Material Sciences (iCeMS), Kyoto University Institute for Advanced Study (KUIAS), Kyoto University, Sakyo-ku, Kyoto 606-8501, Japan.

[c] Department of Chemistry and Polymer Science, Stellenbosch University, Matieland 7600, South Africa.

[d] Department of Chemistry, University of South Florida, Tampa, Florida 33620-5250, United States;

[e] Department of Chemistry, North Carolina State University, Raleigh, North Carolina 27607, United States;

CCDC numbers 2111569-2111573, 2166387.

* xtal@ul.ie

List of Contents

1. Ligand Synthesis S3
2. Crystallisation S4
3. Single-Crystal X-ray Diffraction S4
4. Powder X-ray Diffraction S11
5. Thermogravimetric Analysis S14
6. Differential Scanning Calorimetry S14
7. Gas Sorption Measurements S15
8. *In Situ* Powder X-ray Diffraction S25
9. Switching materials reference table S32
10. Computational Modelling S33
11. References S36

1. Ligand Synthesis

General information

Commercially available starting materials and solvents were purchased from Sigma Aldrich, Merck and Fluorochem. All reactions were monitored using aluminium backed silica gel Merck 60 F_{254} TLC plates and visualised using UV irradiation. Column chromatography was carried out with Merck silica gel 230-400 mesh silica gel.

Synthesis of 1,3-bib was carried out in a single step, according to a previously reported procedure with minor modifications.[1]

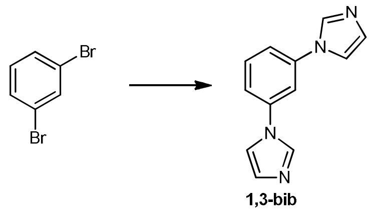

1,3-dibromobenzene (10.0 g, 42.8 mol, 1.0 equiv), imidazole (14.5 g, 213.0 mmol, 5.0 equiv), CuI (1.63 g, 8.6 mmol, 20 mol%) and K_2CO_3 (29.4 g, 213.0 mmol, 5.0 equiv) were all added to anhydrous DMF (150 ml) under an inert N_2 atmosphere. The resulting reaction mixture was heated to reflux under an inert atmosphere for 72 h. After cooling to room temperature, the reaction mixture was filtered. The filtered residue was washed with DCM (2 × 150 ml) and the filtrate was transferred to a large separating funnel. The organic layer was washed with water (3 × 250 ml), separated and dried over $MgSO_4$. The organic layer was concentrated under reduced pressure and the resulting solid material was finally purified by trituration from a DCM/hexane mixture. The resulting 1,3-bib was isolated as a white solid (8.41 g, 94%).

Synthesis of dpt was carried out in two steps, according to a previously reported procedure.[2]

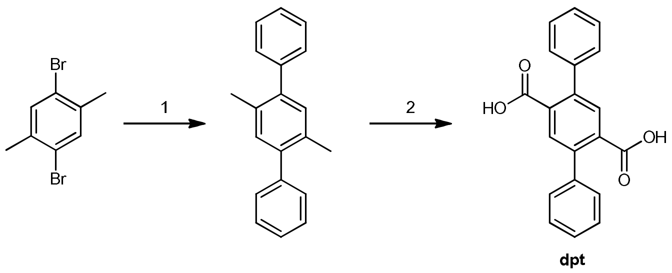

Scheme 1: Two-step synthesis of dpt ligand.

Step 1: 20 ml of deionized water was degassed for 30 min using N_2. 2,5-dibromo-*p*-xylene (2.00 g, 7.58 mmol, 1.0 equiv), phenyl boronic acid (2.03 g, 16.7 mmol, 2.2 equiv), $Pd(OAc)_2$ (3.40 mg, 0.2 mol%), K_2CO_3 (5.24 g, 37.9 mmol, 5.0 equiv) and *n*-Bu_4NBr (4.87 g, 15.2 mmol, 2.0 equiv) were all added. The resulting suspension was heated to 343 K for 4 hours and stirred vigorously under an inert atmosphere. After cooling to room temperature, the reaction mixture was diluted further with water (~ 150 ml) and extracted with hexane. The organic phase was dried over $MgSO_4$ and concentrated under reduced pressure to yield 2',5'-dimethyl-*p*-terphenyl as a white solid (1.88 g, 96%).

Step 2: 2',5'-dimethyl-*p*-terphenyl (700 mg, 2.71 mmol, 1.0 equiv) was added to 20 ml of pyridine. 2.2 g of $KMnO_4$ in 2.0 ml of H_2O was then added and the reaction mixture was heated to reflux for 2 hours. After reaching reflux, every 30 min, an additional 1.0 g of $KMnO_4$ in 2.0 ml was added (a total of 4 times). After 6 hours at reflux, a final 10 ml of water was added to the reaction mixture, which was allowed to reflux overnight. The MnO_2 precipitate was hot filtered from the reaction mixture and washed with near boiling water (100 ml). The filtrate was acidified (pH 3-4) using conc. HCl, precipitating the dpt product as a white solid, which was collected by filtration, washed with 0.2 M HCl and finally dried in a 378 K oven overnight (732 mg, 85%).

2. Crystallisation

Crystals of $\mathbf{1}_{\mathbf{DMF}}$ were grown solvothermally by combining 0.3 mmol (63.0 mg) dpt, 0.3 mmol (95.0 mg) 1,3-bib and 0.3 mmol (87.0 mg) cobalt nitrate hexahydrate in 10 ml DMF and heating at 393 K. Purple block crystals were obtained after two days.

3. Single-Crystal X-ray Diffraction

X-ray intensity data were recorded on a Bruker SMART APEX II[3] and a Bruker Quest APEX III equipped with a Mo or Cu sealed tube source. Both diffractometers employ an Oxford Cryosystems 700 Plus cryostat to control the temperature of the sample. Data reduction was carried out by means of standard procedures using the Bruker software package SAINT.[4] Absorption corrections and correction of other systematic errors were carried out using SADABS.[5] All structures were solved by direct methods using SHELXS-16 and refined using SHELXL-16.[6] X-Seed[7] was used as the graphical interface for the SHELX program suite.

Solvent-accessible voids can be visualised by calculating Connolly surfaces using MS-ROLL,[8] another program incorporated into X-Seed. Hydrogen atoms were placed in calculated positions using riding models.

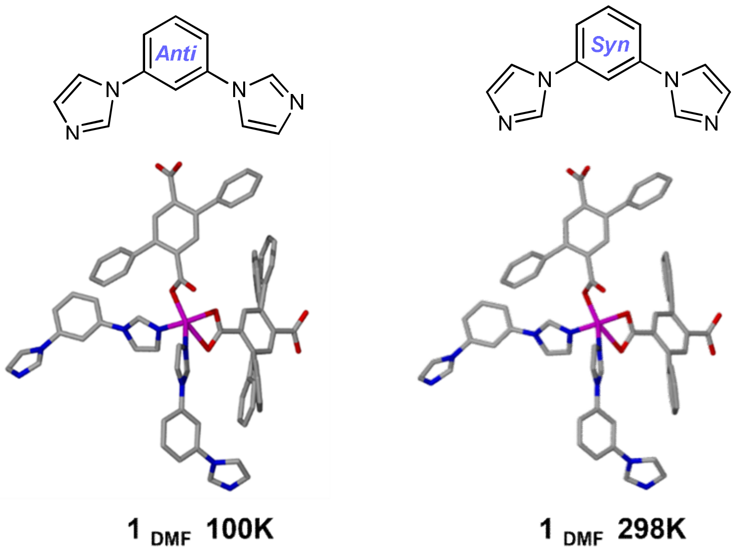

Fig. 1. Coordination modes of $\mathbf{1}_{\mathbf{DMF}}$ at 100 K (left) and 298 K (right). All 1,3-bib ligands adopt a *anti* conformation. Hydrogen atoms have been omitted for clarity.

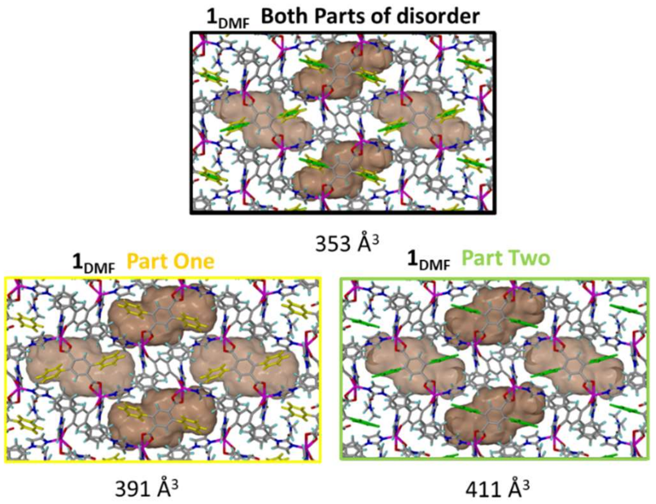

Fig. 2. Packing diagrams viewed down [100] of $\mathbf{1}_{\mathbf{DMF}}$ recorded at 100 K showing the effect of disorder in the dpt ligand (part one = yellow, part two = green) on the guest accessible space (shown as a light brown surface using a 1.5 Å probe radius).

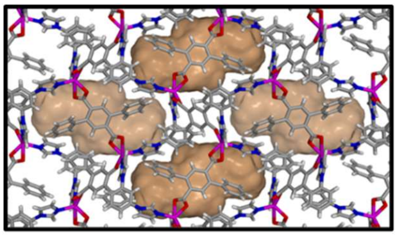

Fig. 3. Packing diagram of $\mathbf{1}_{\mathbf{DMF}}$ recorded at 298 K showing the guest accessible space (shown as a light brown surface using a 1.5 Å probe radius).

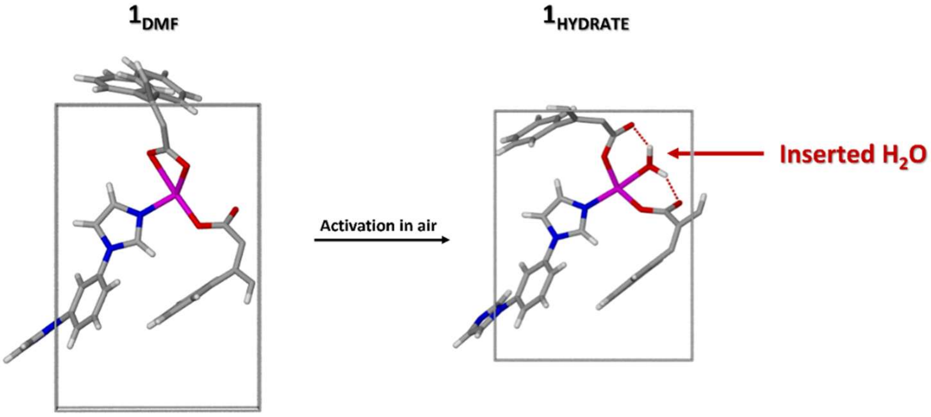

Fig. 4. Capped stick diagrams of $\mathbf{1}_{\mathbf{DMF}}$ and $\mathbf{1}_{\mathbf{Hydrate}}$ recorded at 100 K showing the effect of activation (i.e. heating to 423 K at 25 K/min and exposing the sample to air for a few minutes) on the SBU and the unit cell dimensions.

<u>Activation Procedure</u>

A suitable crystal of the as-synthesised material was selected and glued onto a glass fibre with cyanoacrylate glue. The glass fibre was then inserted into an environmental gas cell (EGC), which consists of a 0.3 mm Lindemann capillary secured to a steel nut with epoxy that is screwed into a valve body. The EGC allows for evacuation/pressurisation of the immediate crystal environment and transportation to a diffractometer. The EGC was then connected to a Pfeiffer Hi-Cube vacuum pump (pressure: ~ 3 x 10^{-3} mbar) and immersed in oil, which was heated to 393 K overnight. The valve was then closed and the EGC removed from the activation apparatus.

Apohost structure determination

The evacuated crystal in a EGC was mounted onto a conventional goniometer and SCXRD data was recorded at 298 K.

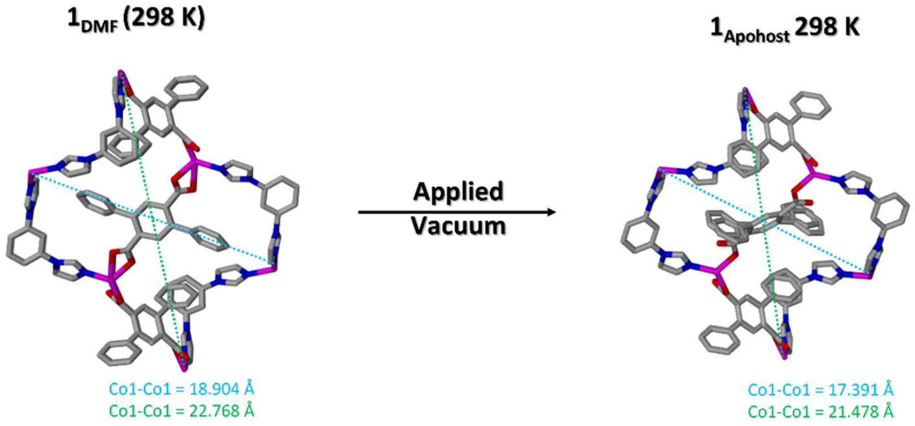

Fig. 5. Capped stick diagrams of $\mathbf{1_{DMF}}$ and $\mathbf{1_{Apohost}}$ recorded at 298 K viewed down [100] showing the effect of evacuation on the cavity dimensions. Hydrogen atoms have been omitted for clarity.

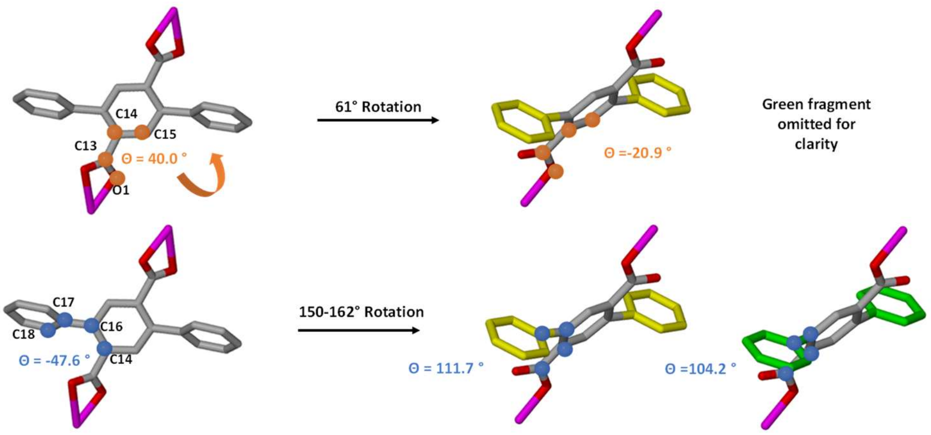

Fig. 6. Capped stick diagrams of $\mathbf{1_{DMF}}$ (left) and $\mathbf{1_{Apohost}}$ (right) recorded at 298 K viewed down [100] showing the rotation of two sets of torsion angles in one of the dpt ligands. Hydrogen atoms have been omitted for clarity.

Gas loading

The activated crystal in the EGC was attached to a CO_2 cylinder via a gas manifold (regulator). The system was pressurised to 1 bar and left to equilibrate under static pressure

for 3 h (multiple equilibration times were tested and this was found to be the best) after which the EGC was closed and loaded onto the diffractometer.

1 bar CO_2 loaded structure determination

In the case of the gas loaded structure (**1'**$_{CO2}$), the crystal was non-merohedrally twinned (split) and was therefore treated as a twinned crystal. Two crystal domains could be separated manually in the reciprocal lattice viewer and were processed as a non-merohedral twin during integration and scaling (TWINABS). Refinement was carried out using only the HKLF4 file, the HKLF5 file did not improve the refinement. An additional TWIN card was used in the refinement (BASF 0.39) to account for minor twinning still present. Thermal parameters were restrained using RIGU and SIMU cards.

The Checkcif contains the following A and B alerts:

Alert level A:

PLAT601_ALERT_2_A Unit Cell Contains Solvent Accessible VOIDS of 278 Ang**3

Response: Owing to the structural change imparted by the guest on the host, the structure undergoes a gate-opening phenomenon wherein the previously negligible cavities expand to accommodate CO_2 guest molecules.

Alert level B

PLAT082_ALERT_2_B High R1 Value .... 0.16 Report

PLAT341_ALERT_3_B Low Bond Precision on C-C Bonds .... 0.01613 Ang.

Response: The crystal of **1'**$_{CO2}$ was non-merohedrally twinned. We believe this to be as a result of the structural transformations that occur as **1**$_{DMF}$ converts to **1**$_{Apohost}$ and then to **1'**$_{CO2}$. This coupled with a reduction in peak intensity resulted in a high R1 value.

56 bar CO_2 loaded structure determination

The Checkcif contains the following B alerts:

RINTA01_ALERT_3_B The value of Rint is greater than 0.18 .... Rint given 0.200

PLAT020_ALERT_3_B The Value of Rint is Greater Than 0.12 .... 0.200 Report

PLAT341_ALERT_3_B Low Bond Precision on C-C Bonds .... 0.01577 Ang.

Response: As the $\mathbf{1_{apohost}}$ crystal undergoes progressive gas loading, it first converts to $\mathbf{1'_{CO2}}$ and then to $\mathbf{1''_{CO2}}$. These phase transformations induce striation within the crystal resulting in reduced peak intensity, an elevated Rint and reduced bond precision. Our goal for this experiment was to attain the $\mathbf{1'''_{CO2}}$ phase however at 56 bar CO_2 and 298 K, the crystal of $\mathbf{1_{apohost}}$ has converted to $\mathbf{1''_{CO2}}$.

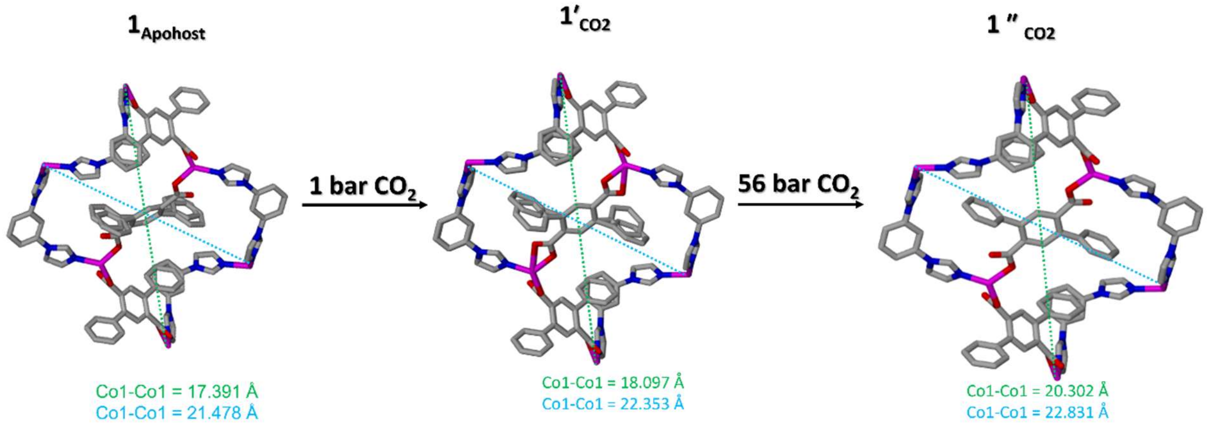

Fig. 7. Capped stick diagrams of $\mathbf{1_{Apohost}}$, $\mathbf{1'_{CO2}}$ and $\mathbf{1''_{CO2}}$ recorded at 298 K viewed down [100] showing the effect of gas loading on the cavity dimensions. Guest molecules and hydrogen atoms have been omitted for clarity.

Table 1. Selected crystallographic data for structures $\mathbf{1_{DMF}}$-$\mathbf{1''_{CO2}}$.

CCDC numbers 2111569-2111573, 2166387.

	$\mathbf{1_{DMF}}$	$\mathbf{1_{DMF}}$	$\mathbf{1_{Hydrate}}$	$\mathbf{1_{Apohost}}$	$\mathbf{1'_{CO2}}$	$\mathbf{1''_{CO2}}$
Empirical Formula	$C_{36.14}H_{31.66}CoN_{5.38}O_{5.38}$	$C_{35.81}H_{29.89}CoN_{5.27}O_{5.27}$	$C_{32}H_{23}CoN_4O_{5.56}$	$C_{32}H_{21.45}CoN_4O_4$	$C_{32}H_{22}CoN_4O_4 \cdot nCO_2$	$C_{32}H_{22}CoN_4O_4 \cdot nCO_2$
Formula weight	686.34	677.29	611.43	584.91	585.47	585.47
Guest	DMF	DMF	H_2O	-	CO_2	CO_2
Temperature (K)	100(2)	298(2)	98(2)	298(2)	298(2)	298(2)
Wavelength (Å)	0.71073	0.71073	0.71073	0.71073	0.71073	0.71073
Crystal system	Monoclinic	Monoclinic	Monoclinic	Monoclinic	Monoclinic	Monoclinic
Space group	$P2_1/n$	$P2_1/n$	$P2_1/n$	$P2_1/n$	$P2_1/n$	$P2_1/n$
a/Å	10.031(2)	10.094(1)	10.202(1)	9.843(7)	9.903(3)	10.007(3)
b/ Å	14.620(3)	14.803(6)	12.867(2)	13.890(1)	14.543(4)	14.869(3)
c/ Å	23.674(5)	23.871(5)	22.014(5)	22.608(3)	23.049(6)	23.493(4)
α°	90	90	90	90	90	90
β°	99.877(3)	100.436(1)	92.368(2)	96.289(1)	99.113(9)	100.746(6)
γ°	90	90	90	90	90	90
Volume (Å^3)	3420.5(1)	3508.1(3)	2887.4(4)	3072.6(3)	3277.7(2)	3434.4(1)
Z	4	4	4	4	4	4
Rint	6.52%	5.01%	7.46%	7.78%	16.0%	19.0%
*R*1	0.0872	0.0716	0.0685	0.0609	0.1594	0.1300
*wR*2	0.2489	0.2276	0.1877	0.1255	0.3550	0.3543
S	1.113	1.075	1.023	1.097	1.240	1.032

Table 1. Continued. Ortep diagrams for structures $\mathbf{1_{DMF}}$-$\mathbf{1''_{CO2}}$

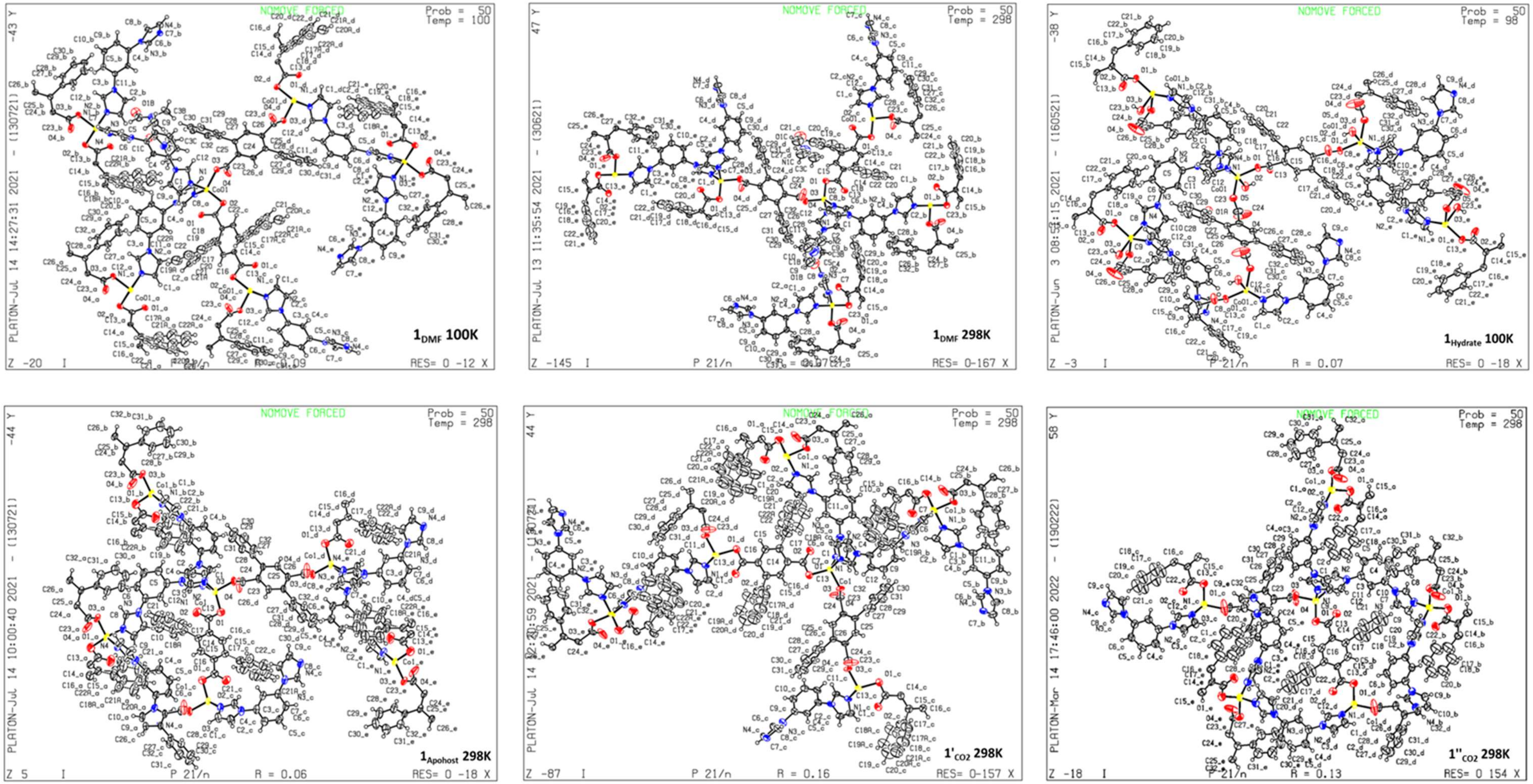

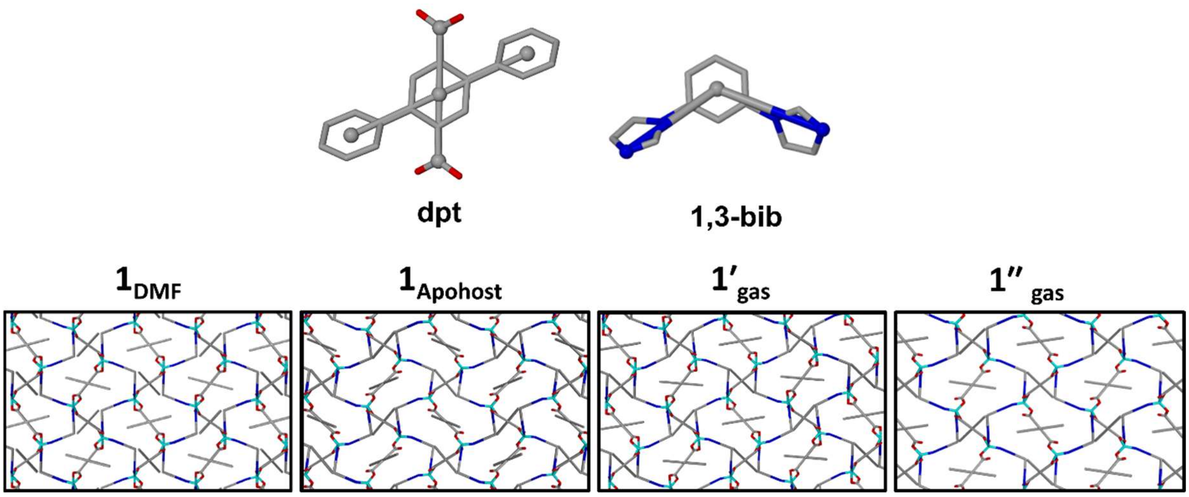

Fig. 8. Node and linker plot comparison of $\mathbf{1_{DMF}}$, $\mathbf{1_{Apohost}}$ $\mathbf{1'_{CO2}}$ and $\mathbf{1''_{CO2}}$. Guest molecules have been omitted for clarity.

4. Powder X-ray Diffraction

Powder X-ray diffraction experiments were conducted using microcrystalline samples on a PANalytical Empyrean diffractometer (40 kV, 40 mA, Cu $K_{\alpha1,2,}$ λ = 1.5418 Å) in Bragg-Brentano geometry. A scan speed of 0.044509 °/s (2.6 °/min), with a step size of 0.0262 ° in 2θ was used at room temperature with a range of 5 °< 2θ < 40 °. Powder samples were evenly distributed on a zero-background holder after being ground with a mortar and pestle to minimise the effects of preferred orientation. Data analysis was carried out using X'Pert HighScore Plus[9] (Version 2.2e). Powder patterns were simulated from SCXRD structures using Mercury.[10]

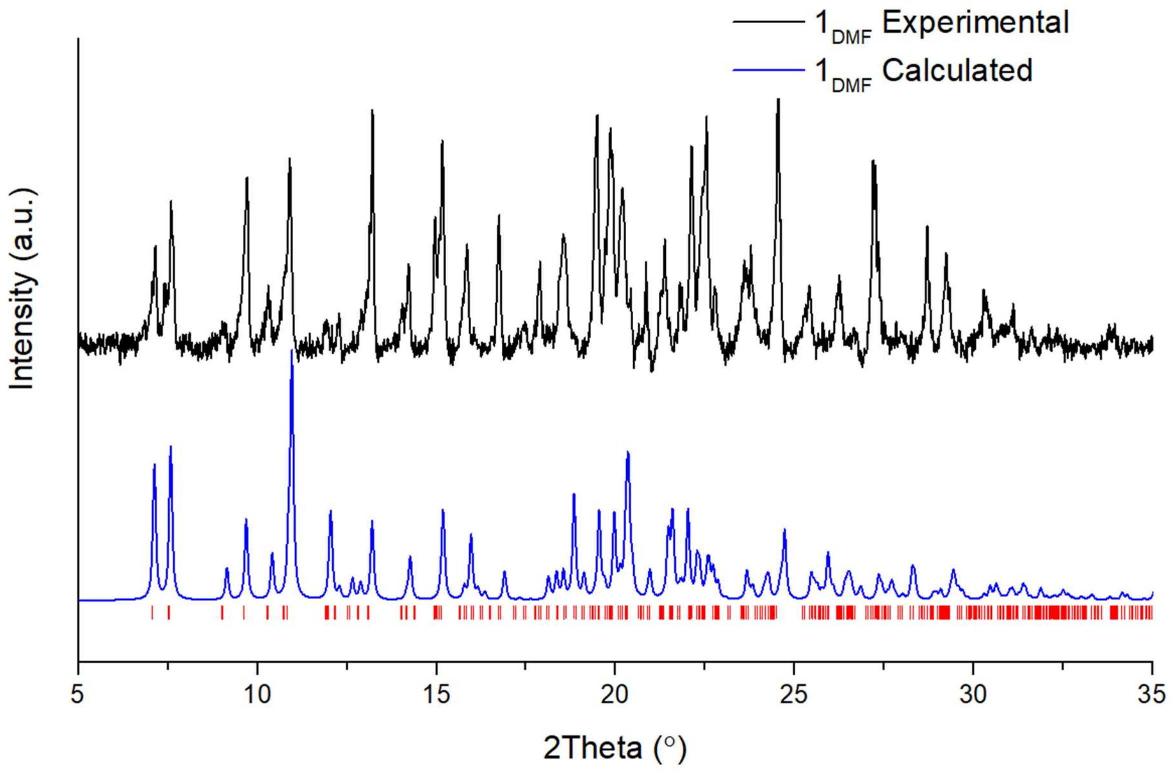

Fig. 9. PXRD patterns of $\mathbf{1_{DMF}}$ calculated from the crystal structure (blue), and the experimental pattern (black) of $\mathbf{1_{DMF}}$, showing that the SCXRD structure is representative of the bulk material.

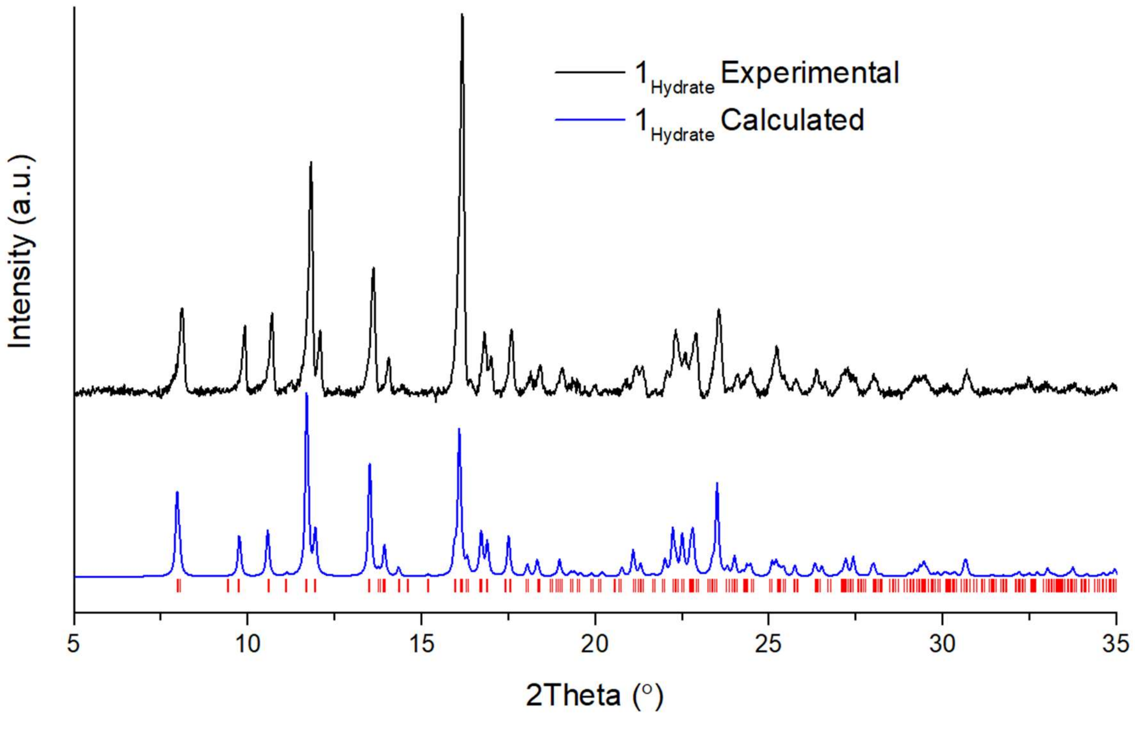

Fig. 10. PXRD patterns of $\mathbf{1_{Hydrate}}$ calculated from the crystal structure (blue), and the experimental pattern (black) of $\mathbf{1_{Hydrate}}$, showing that the SCXRD structure is representative of the bulk material.

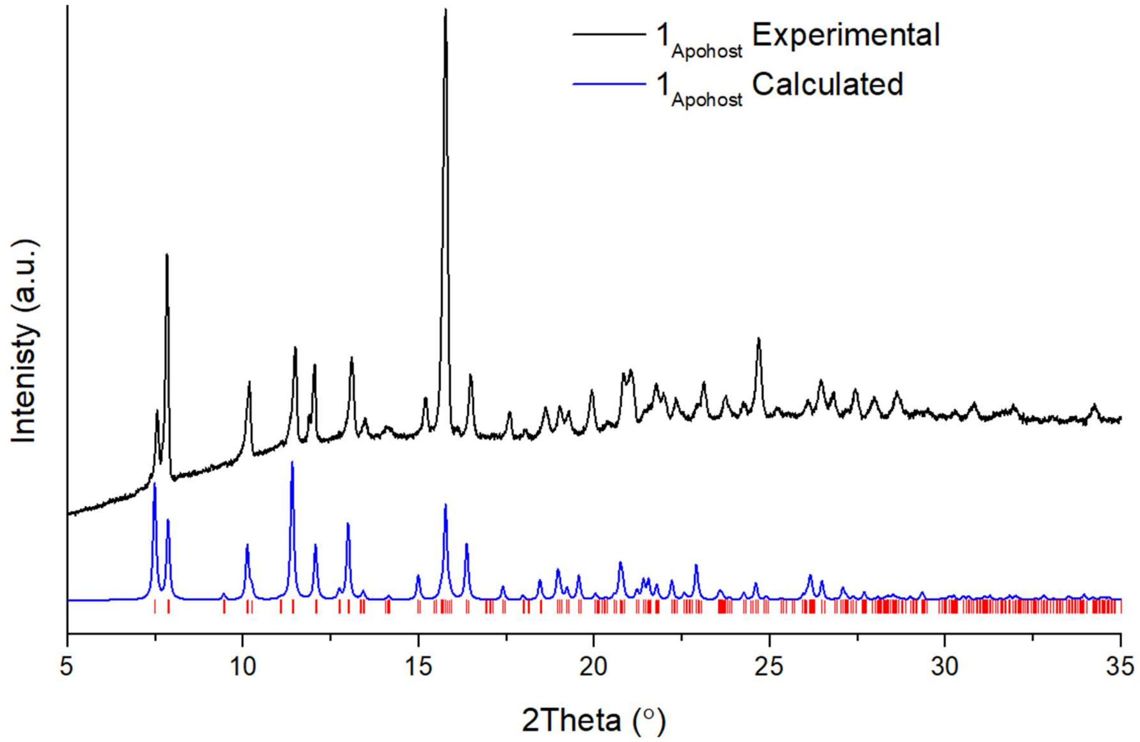

Fig. 11. PXRD patterns of $\mathbf{1}_{\mathbf{Apohost}}$ calculated from the crystal structure (blue), and the experimental pattern (black) of $\mathbf{1}_{\mathbf{Apohost}}$, showing that the SCXRD structure is representative of the bulk material.

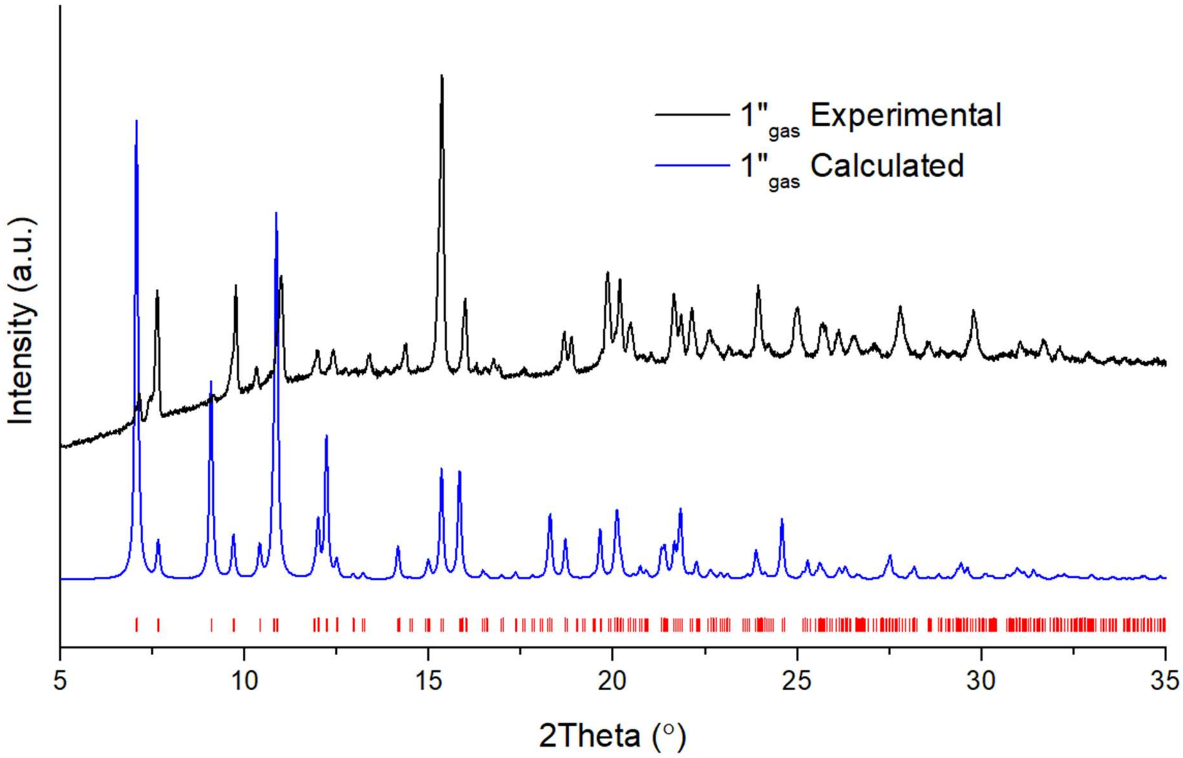

Fig. 12. PXRD patterns of $\mathbf{1}''_{\mathbf{gas}}$ calculated from the crystal structure (blue), and the experimental pattern (black) of $\mathbf{1}''_{\mathbf{gas}}$, showing that the SCXRD structure is representative of the bulk material.

5. **Thermogravimetric Analysis (TGA)**

Thermogravimetric analyses (TGA) were performed under N_2 using a TA Instruments Q50 system. A sample was loaded into an aluminium sample pan and heated at 283 K min^{-1} from room temperature to 773 K.

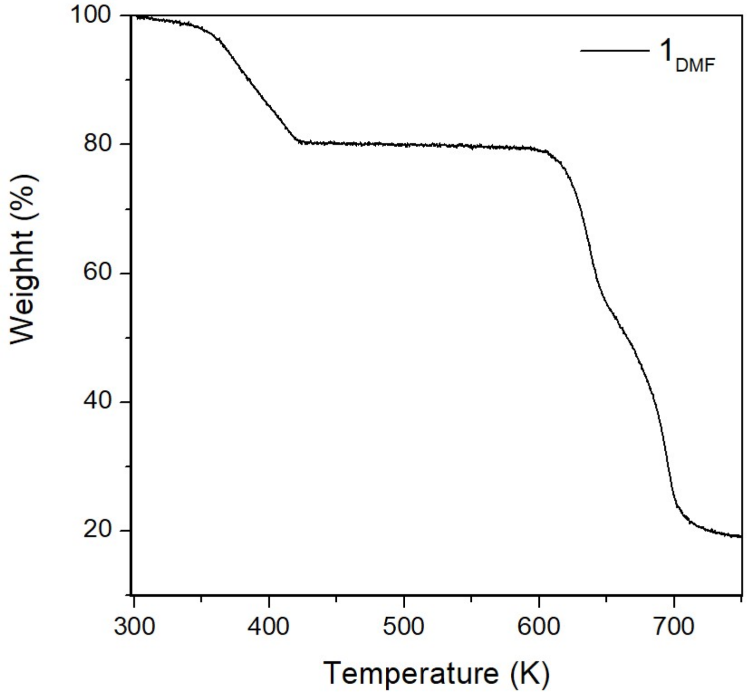

Fig. 13. Thermogravimetric trace for $\mathbf{1_{DMF}}$ showing a 19.64% mass loss between 273-423 K (corresponding to two DMF guest molecules per ASU). The material is then stable until decomposition begins at 603 K.

6. **Differential Scanning Calorimetry (DSC)**

Differential scanning calorimetry was carried out using a TA Instruments Q2000 differential scanning calorimeter. Samples were prepared by crimping the sample pan and lid (a pin hole was placed in the lid to prevent pressure build-up). A reference pan was prepared in the same manner for each analysis. Analyses were generally carried out in the temperature range 253 K - 523 K and a general experimental procedure consisted of two heating/cooling cycles while the heat flow into or out of the sample, relative to the reference, was measured as a function of time and temperature in a controlled atmosphere. N_2 gas, flowing at a rate of 50 ml min^{-1} was used to purge the furnace. The resulting thermograms were analysed

using TA Instruments Universal Analysis program and Figs. were prepared with Microsoft Excel.

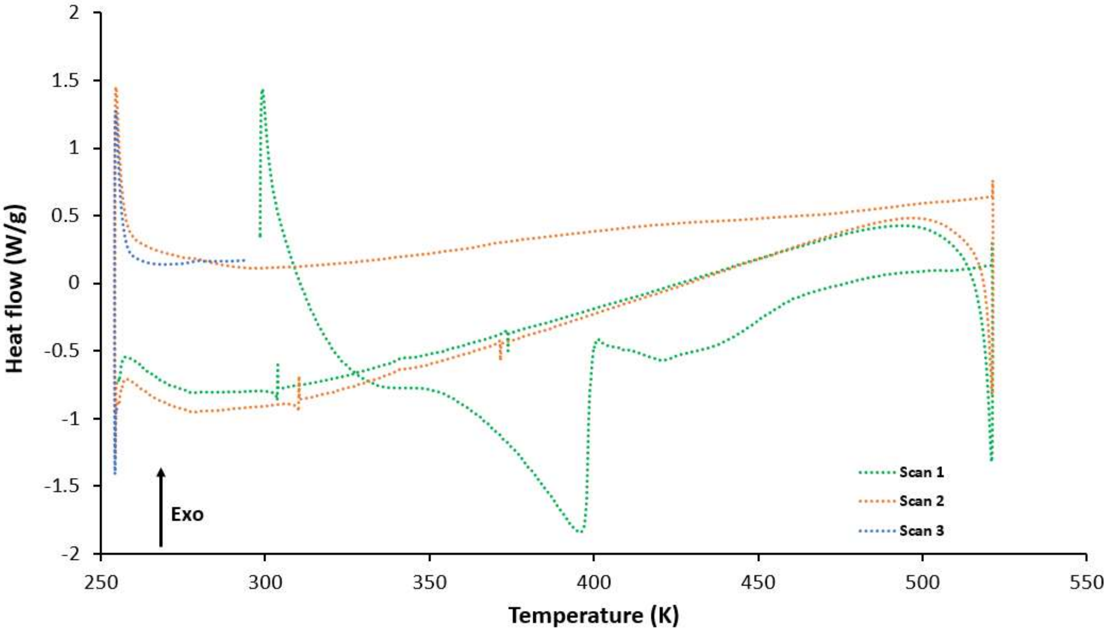

Fig. 14. DSC thermogram of **1_{DMF}** showing two consecutive runs. The sample was initially heated from 298 K to 523 K, cooled to 253 K (green line) and then the run was repeated (orange line). An endothermic peak is observed between 333 K - 403 K corresponding to solvent loss.

7. Gas Sorption Measurements

7.1 Isotherm Measurements

Prior to performing gas sorption experiments, a freshly prepared sample of **1_{DMF}** was placed in a quartz tube and degassed under high vacuum using a Smart VacPrep instrument at 343 K for 24 hours to remove any remaining solvent molecules and yield **$1_{Apohost.}$** Isotherms were measured using a Micromeritics 3 Flex sorption analyser. Gases were used as obtained from BOC Gases Ltd. (Ireland), with the following certified purities: research-grade He (99.999%), CO_2 (99.995%), C_2H_2 (98.5%), C_2H_4 (99.92%), C_2H_6 (99.0%) and N_2 (99.998%). Bath temperatures of 77 K and 195 K were maintained using liquid nitrogen and a dry ice-acetone slurry respectively. A Julabo ME v.2 temperature controller was used to maintain bath temperatures in 273 K and 298 K experiments. Samples were activated between successive experiments overnight or for a minimum of 5 hours at 343 K under high vacuum. High pressure CO_2 sorption experiments were performed using a Hiden Isochema XEMIS microbalance. Activated samples of **$1_{Apohost}$** were further outgassed under secondary vacuum for 3 hours *in situ* before isotherms were run. Excess adsorption and desorption profiles

were obtained after applying a buoyancy correction using the crystallographically determined density of **1**$_{\mathbf{Apohost}}$.

Temperatures were maintained at 273 K and 294 K using a Grant LT Ecocool 150 temperature controller. Equilibration is determined by a cut-off criterion of agreement within 0.01% of a pressure reading with the rolling average of the previous ten pressure readings each collected after allowing the equilibration interval (10 s for 195 K CO_2 experiments, and 77 K N_2 experiments; 30 s for 273 K and 298 K experiments) to elapse. Equilibration data were recorded for the full range of pressure dosing during the 298 K CO_2 and C_2H_2 with a sampling interval of *ca.*2 pressure readings per second.

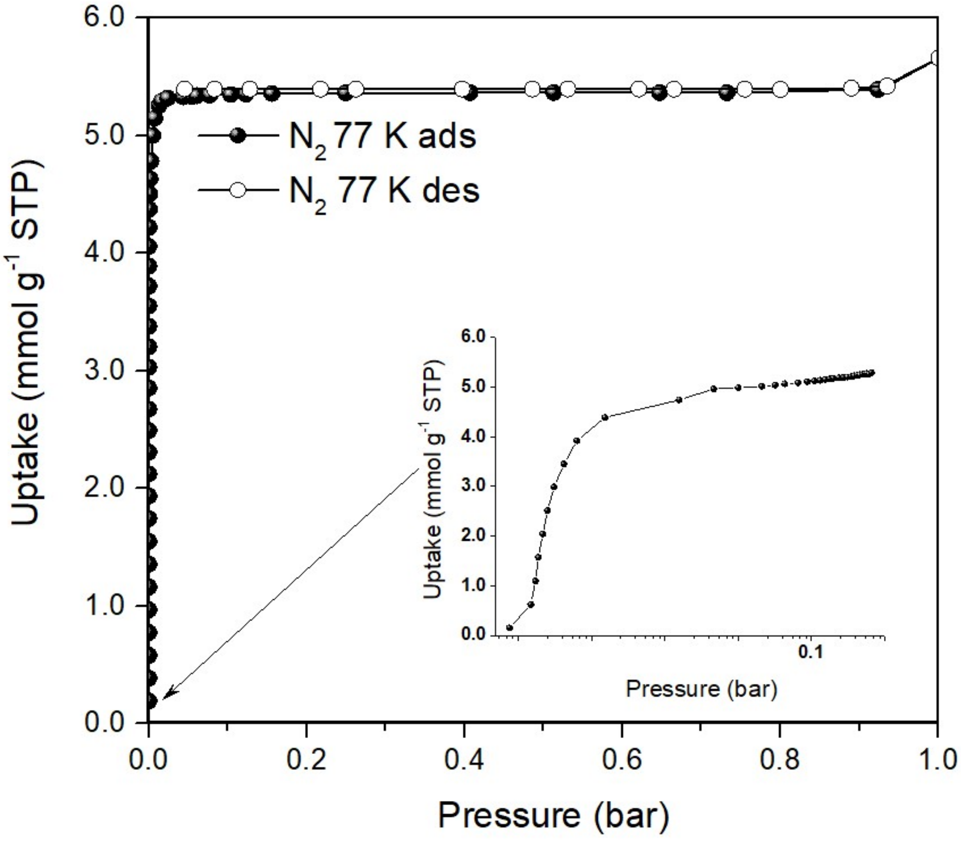

Fig. 15. N_2 gas sorption isotherms of **1**$_{\mathbf{Apohost}}$ at 77 K with a log plot inset of the low pressure region.

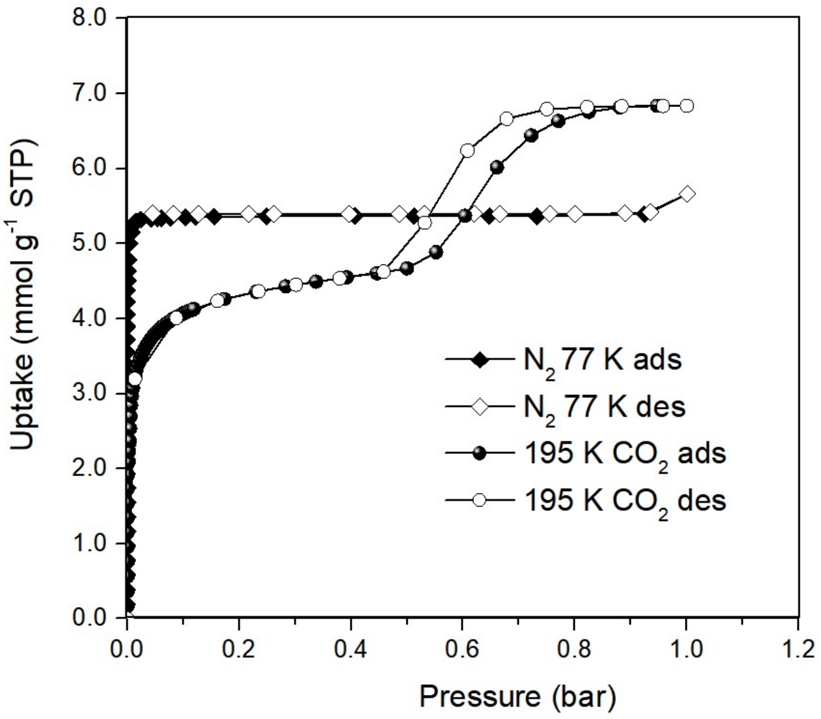

Fig. 16. Overlaid CO_2 (spheres) and N_2 (diamonds) gas sorption isotherms of $\mathbf{1}_{\mathbf{Apohost}}$ at 195 K and 77 K respectively

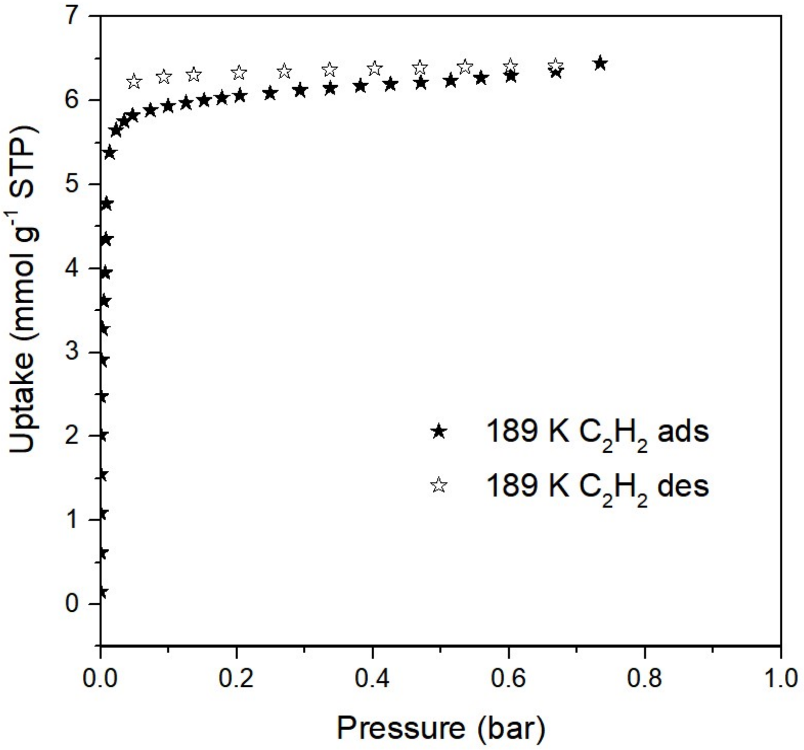

Fig. 17. C_2H_2 gas sorption isotherms of $\mathbf{1}_{\mathbf{Apohost}}$ at 189 K.

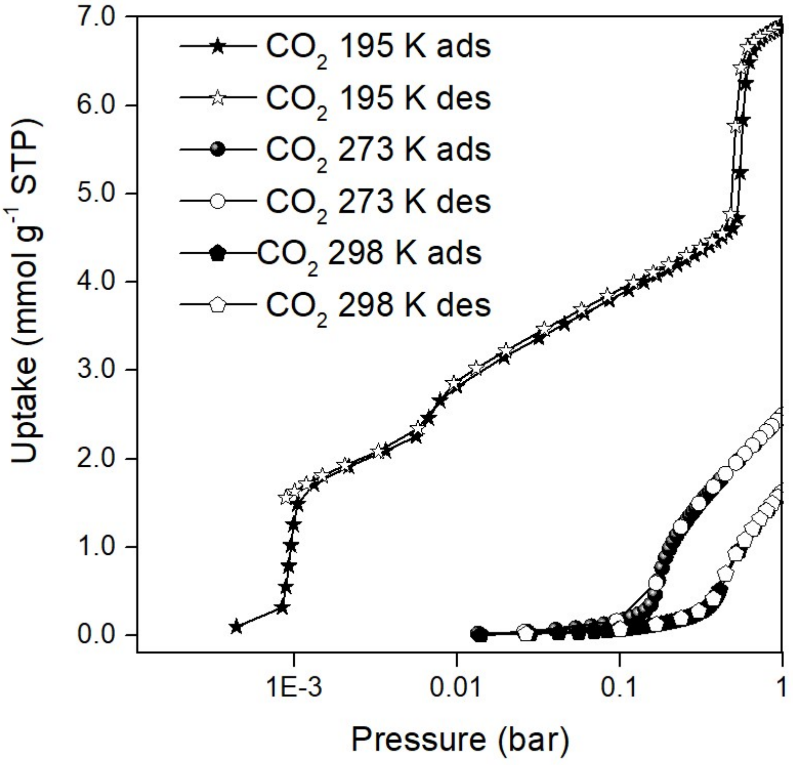

Fig. 18. Overlaid CO_2 gas sorption isotherms of $\mathbf{1}_{\mathbf{Apohost}}$ at 195 K (stars), 273 K (spheres) and 298 K (pentagons) plotted using a logarithmic scale. The material appears closed until a sudden sharp step occurs at 1E-3, 0.2 and 0.3 bar at 195 K, 273 K and 298 K respectively.

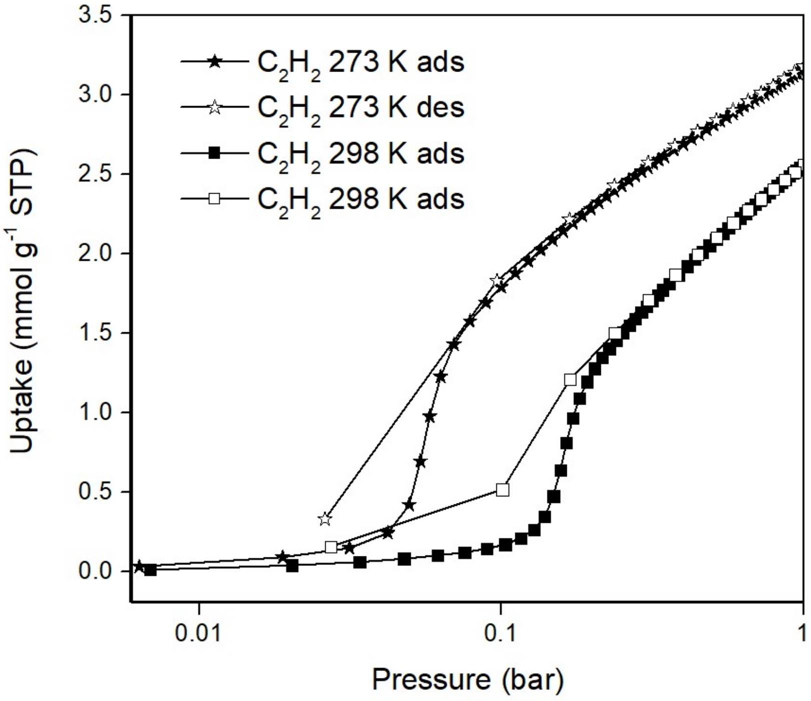

Fig. 19. Overlaid C_2H_2 gas sorption isotherms of $\mathbf{1}_{\mathbf{Apohost}}$ at 273 K (stars) and 298 K (squares) plotted using a logarithmic scale. The material appears closed until a sudden sharp step occurs at 0.03 and 0.08 bar at the respective temperatures.

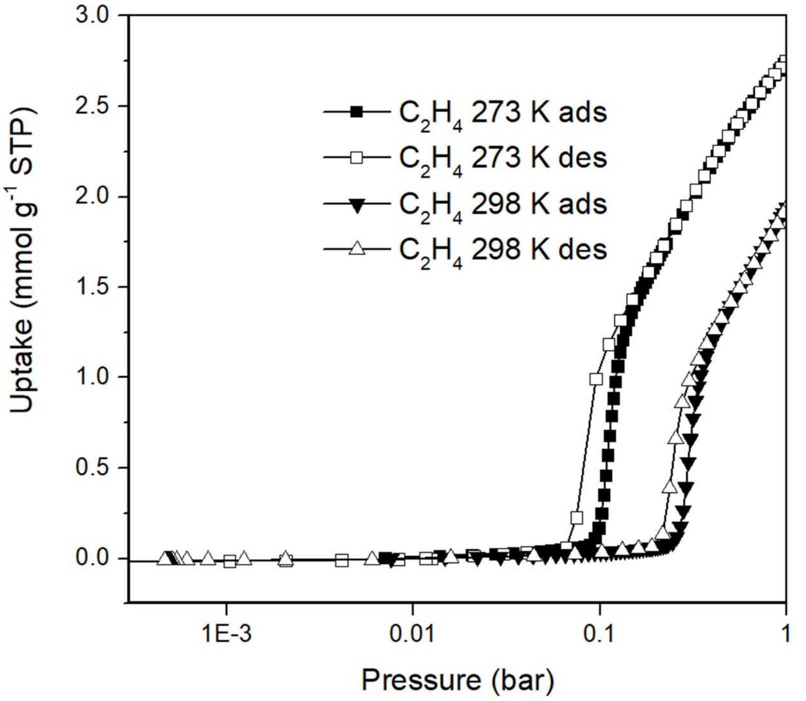

Fig. 20. Overlaid C_2H_4 gas sorption isotherms of **1**$_{\mathbf{Apohost}}$ at 273 K (squares) and 298 K (triangles) plotted using a logarithmic scale. The material appears closed until a sudden sharp step occurs at 0.1 and 0.3 bar at the respective temperatures.

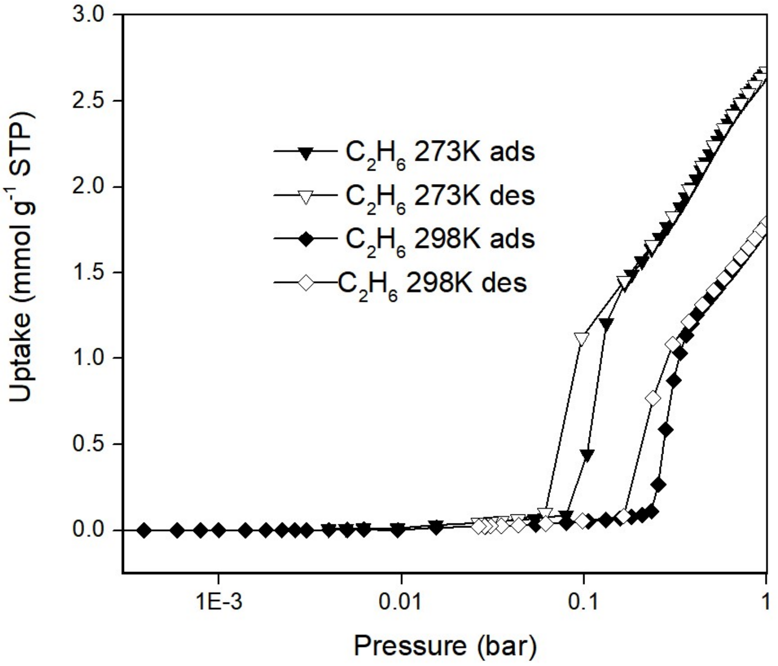

Fig. 21. Overlaid C_2H_6 gas sorption isotherms of **1**$_{\mathbf{Apohost}}$ at 273 K (triangles) and 298 K (diamonds) plotted using a logarithmic scale. The material appears closed until a sudden sharp step occurs at 0.08 and 0.3 bar at the respective temperatures.

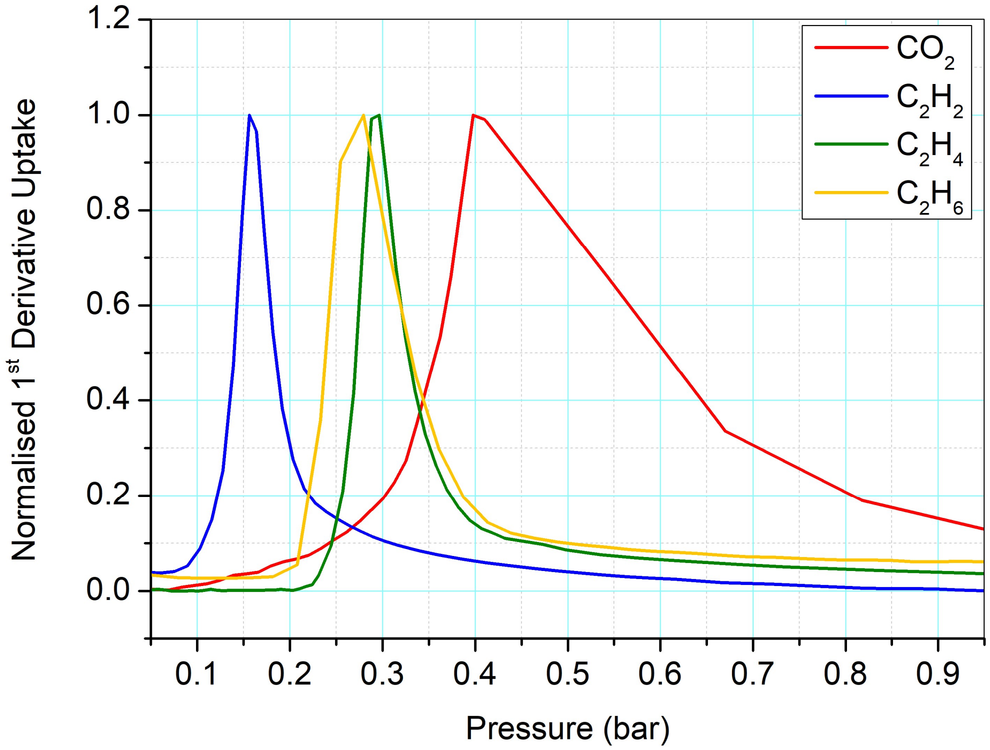

Fig. 22. Normalised first derivatives of the adsorption isotherms of **1$_{\mathbf{Apohost}}$** plotted against pressure at 298 K.

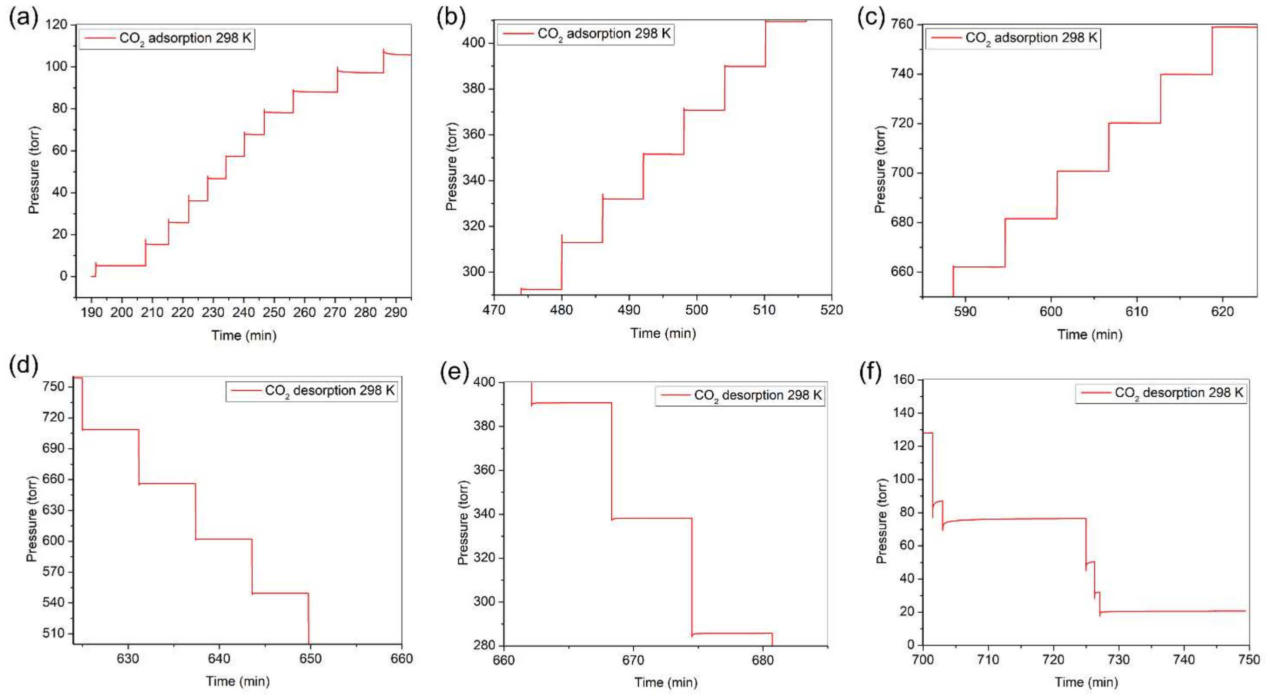

Fig. 23. Real-time equilibration data collected during the CO_2 isotherm of **1$_{\mathbf{Apohost}}$** at 298 K; (a) at low pressures during adsorption, (b) at intermediate pressures during adsorption, (c) at high pressures during adsorption, (d) at high pressures during desorption, (e) at intermediate pressures during desorption, and (f) at low pressures during adsorption.

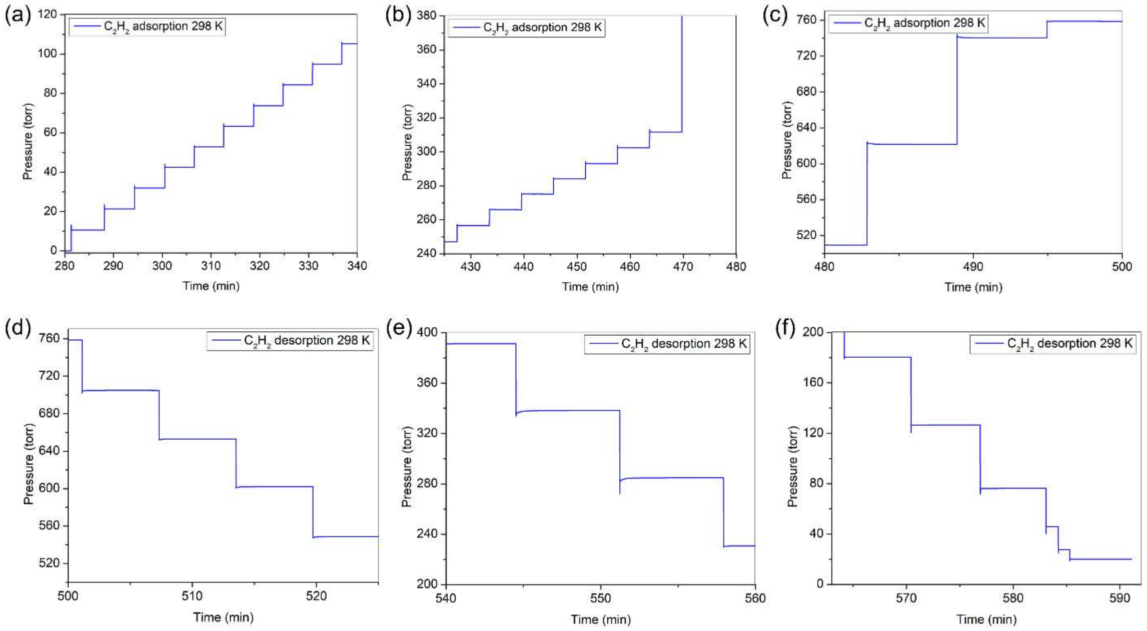

Fig. 24. Real-time equilibration data collected during the C_2H_2 isotherm of $\mathbf{1}_{\mathbf{Apohost}}$ at 298 K; (a) at low pressures during adsorption, (b) at intermediate pressures during adsorption, (c) at high pressures during adsorption, (d) at high pressures during desorption, (e) at intermediate pressures during desorption, and (f) at low pressures during adsorption.

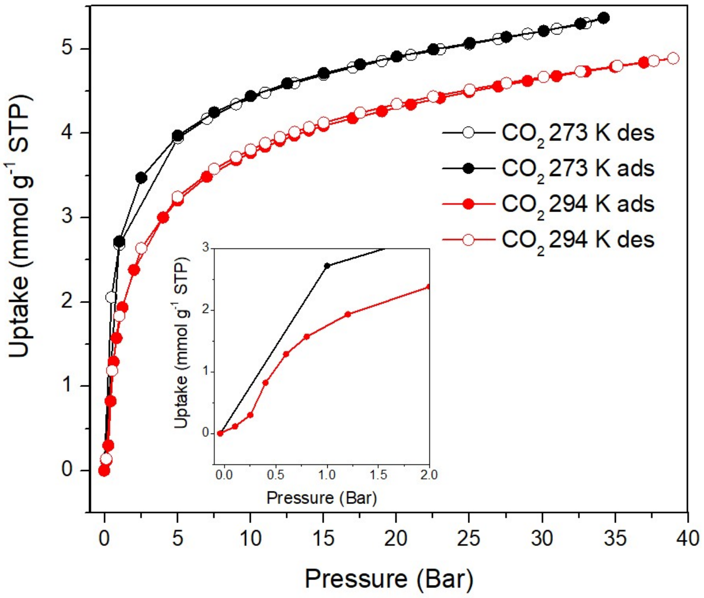

Fig. 25. High pressure CO_2 sorption isotherms of $\mathbf{1}_{\mathbf{Apohost}}$ at 273 K (black spheres) and 294 K (red spheres). Low pressure insert shows the transition of $\mathbf{1}_{\mathbf{Apohost}}$ to $\mathbf{1'}_{\mathbf{CO2}}$ at 294 K. Threshold uptake for final phase transition ($\mathbf{1'''}_{\mathbf{CO2}}$) is not accessible at 273 K, even at $P/P_0 = 1$ (P = 34200 mbar).

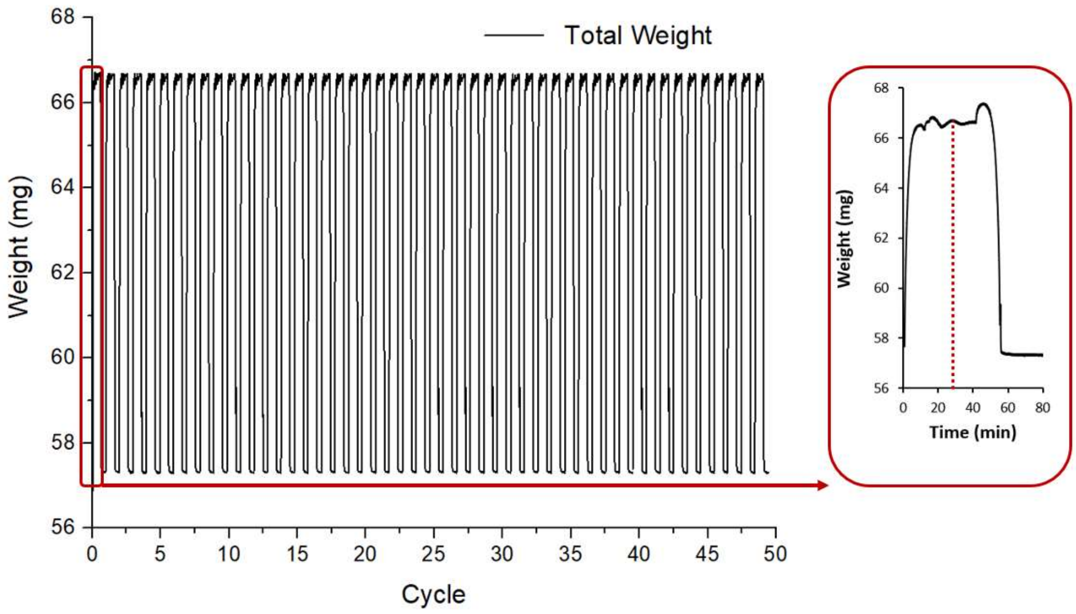

Fig. S26. High pressure CO_2 sorption cycling experiment for **1$_{Apohost}$** at 298 K under the conditions 0-30 bar for 50 cycles. Inset shows the time allocated for one full adsorption-desorption cycle.

7.2 BET Surface Area

BET surface area corresponding to 516.6 m²/g was determined from the 77 K N_2 sorption isotherm (Fig. S15).[11]

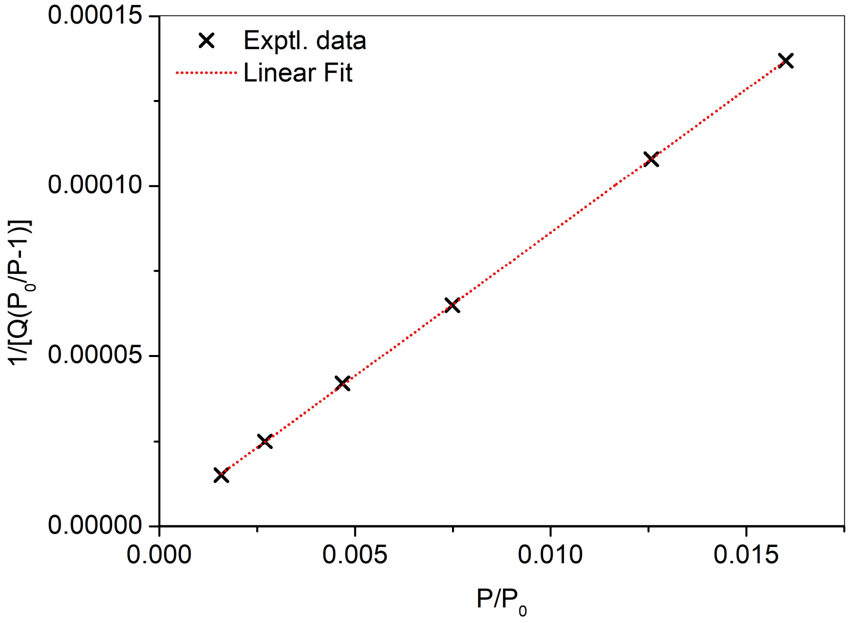

Fig. 27. Multi-point BET plot and linear fit from the linear region of the 77 K N_2 isotherm on **1$_{Apohost}$**.

Table 2. BET fit summary of the 77 K N_2 isotherm on $\mathbf{1_{Apohost}}$.

**BET surface area**	516.61 ± 1.140 m²/g
**Slope**	0.00842 ± 0.00002 g/cm³ STP
**Y-intercept**	0.000002 ± 0.000000 g/cm³ STP
**C**	3,651.572
**Q_m**	118.69 cm³/g STP
**Correlation coefficient**	>0.9999

7.3 Single Point Pore Volumes

Indicative single point pore volumes were determined experimentally using the Microactive software suite under the assumption of approximate validity of the Gurvich rule[12-13] wherein the structural transformations follow a state approaching saturation of the preceding phase during 195 K CO_2 adsorption experiments as described in Eq. 1, where v_{pore}. is the pore volume, n_{CO}^{Ads} is the quantity of gaseous CO_2 adsorbed as determined at STP, and $\rho_{CO2}^{Liq.}$ Is the density of liquid CO_2.

$$v_{\text{pore}} = \frac{n_{CO2}^{Ads}}{\rho_{CO2}^{Liq.}} \qquad \text{Eq.1}$$

The determined volumes are presented with 5% error bars (Table S3) and show reasonable agreement with the assignment of phases based on *in situ* PXRD, and SCXRD determined solvent-accessible volumes.

Table 3. Experimentally determined single-point pore volumes during adsorption of CO_2 at 195 K on **1**.

	P/P_0	Pore Volume (cm^3 g^{-1})
$\mathbf{1_{apohost}}$	0.00499	0.0059±0.0003
$\mathbf{1'_{gas}}$	0.00296	0.0771±0.0039
$\mathbf{1''_{gas}}$	0.3761	0.1574±0.0079
$\mathbf{1'''_{gas}}$	0.9597	0.2365±0.0118

7.4 Molar Enthalpy of Gate Opening

The molar enthalpy of gate opening (ΔH_{GO}) was calculated from the gate opening pressures (P_{GO}) determined from pure component isotherms at 273 K and 298 K, using the Clausius-Clapeyron equation as described in Eq. 2. Enthalpies are found to be correlated with molar enthalpies of vaporisation in agreement with Li *et al.*[14]

$$\Delta H_{GO} = RT^2\left(\frac{\partial \ln P_{GO}}{\partial T}\right) \quad \text{Eq. 2}$$

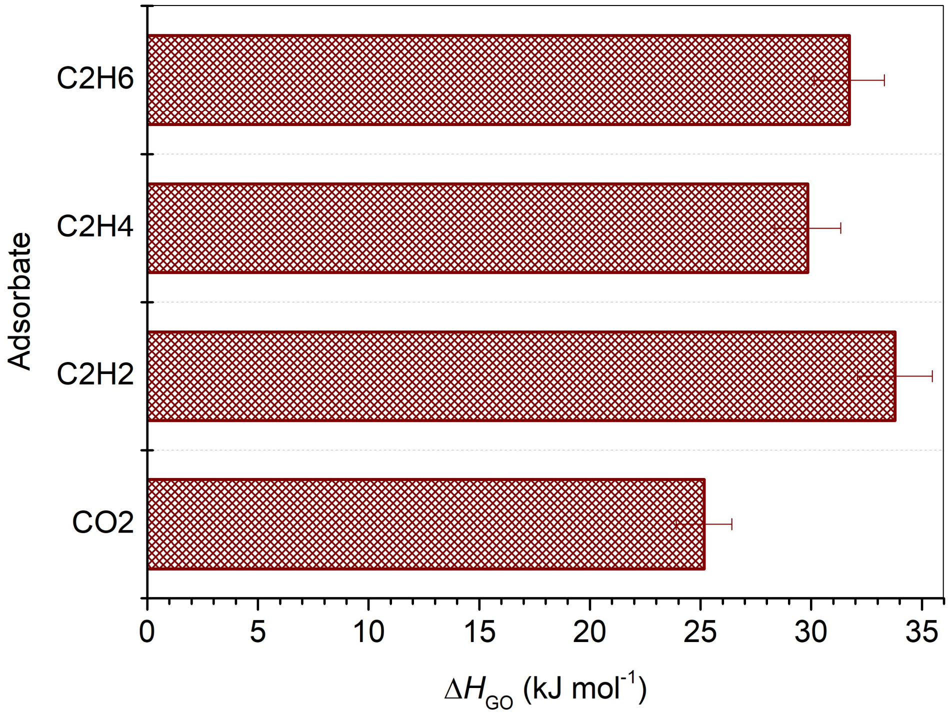

Fig. 28. ΔH_{GO} values determined for various adsorbates on $\mathbf{1_{Apohost}}$. Error bars represent 5% margins.

8. *In situ* Powder Xray diffraction

In-situ coincident PXRD measurements were conducted on a Rigaku Smartlab with CuKα radiation (Rigaku, Japan) which is synchronised to a BELSORP-18PLUS volumetric adsorption instrument (MicrotracBEL Japan, Corp.). A Helium based cryosystem was connected to the sorption equipment to control the temperature range. The as-synthesised sample was soaked in MeOH for three days and then activated at 343 K under vacuum overnight using a copper plate holder. The activated sample of ~70 mg was transferred to the sorption instrument and treated again under vacuum at 353 K for 2 h. The second activation was performed to remove any adsorbed moisture during transfer.

This is an essential step as the sample adsorbs water from the atmosphere. A CO_2 sorption experiment was carried out up to 100 kPa at 195 K. *In-situ* PXRD patterns were measured simultaneously at each equilibrium point of the adsorption and desorption isotherm. In addition to CO_2, *in situ* PXRD patterns were measured at each equilibrium point of the adsorption isotherm for N_2 at 77 K and C_2H_2 at 189 K.

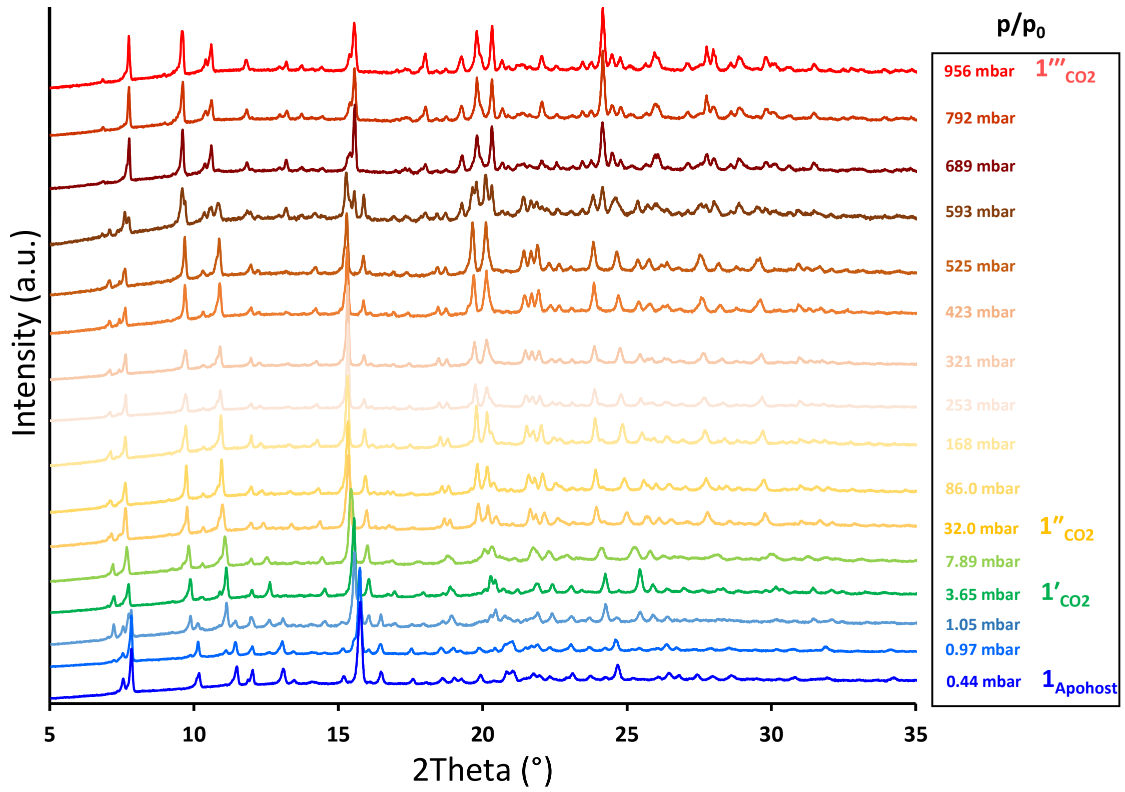

Fig. 29. Selected *in situ* variable pressure PXRD patterns of $\mathbf{1}_{\mathbf{Apohost}}$ at different CO_2 adsorption loadings at 195 K. $\mathbf{1}_{\mathbf{Apohost}}$ undergoes multiple structural transformations from closed to three progressively more gas loaded forms $\mathbf{1'}_{\mathbf{CO2}}$, $\mathbf{1''}_{\mathbf{CO2}}$ and $\mathbf{1'''}_{\mathbf{CO2}}$.

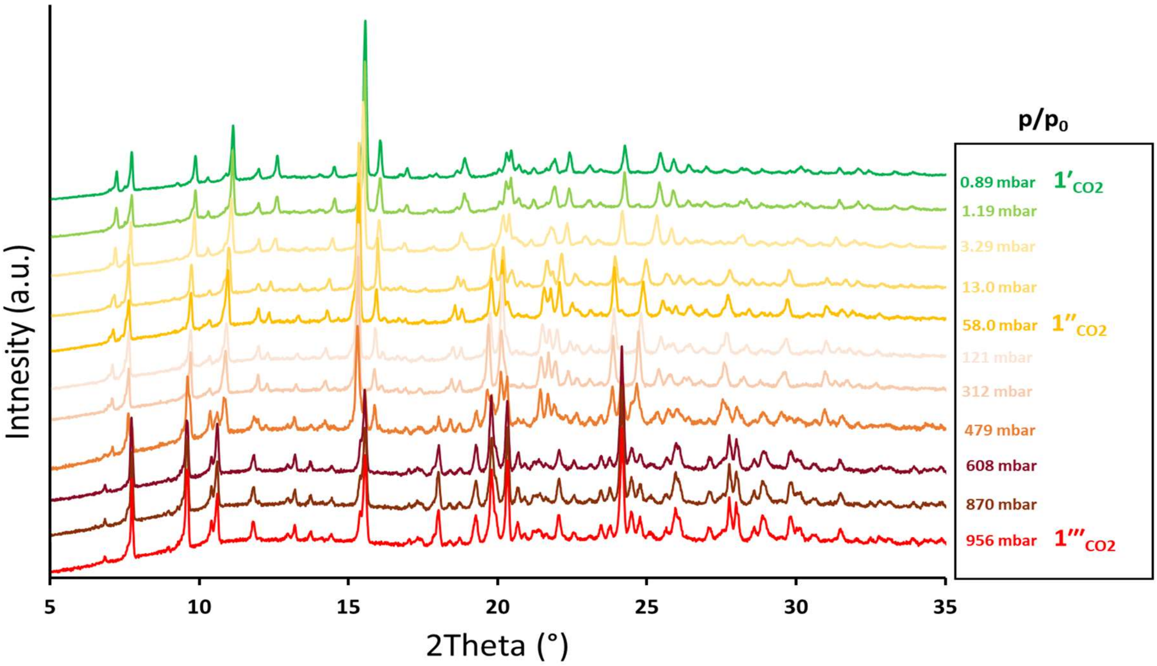

Fig. 30. Selected *in situ* variable pressure PXRD patterns of $\mathbf{1_{Apohost}}$ at different CO_2 desorption loadings at 195 K. $\mathbf{1_{Apohost}}$ undergoes multiple structural transformations from the $\mathbf{1'''_{CO2}}$ to two progressively less gas loaded forms $\mathbf{1''_{CO2}}$ and $\mathbf{1'_{CO2}}$.

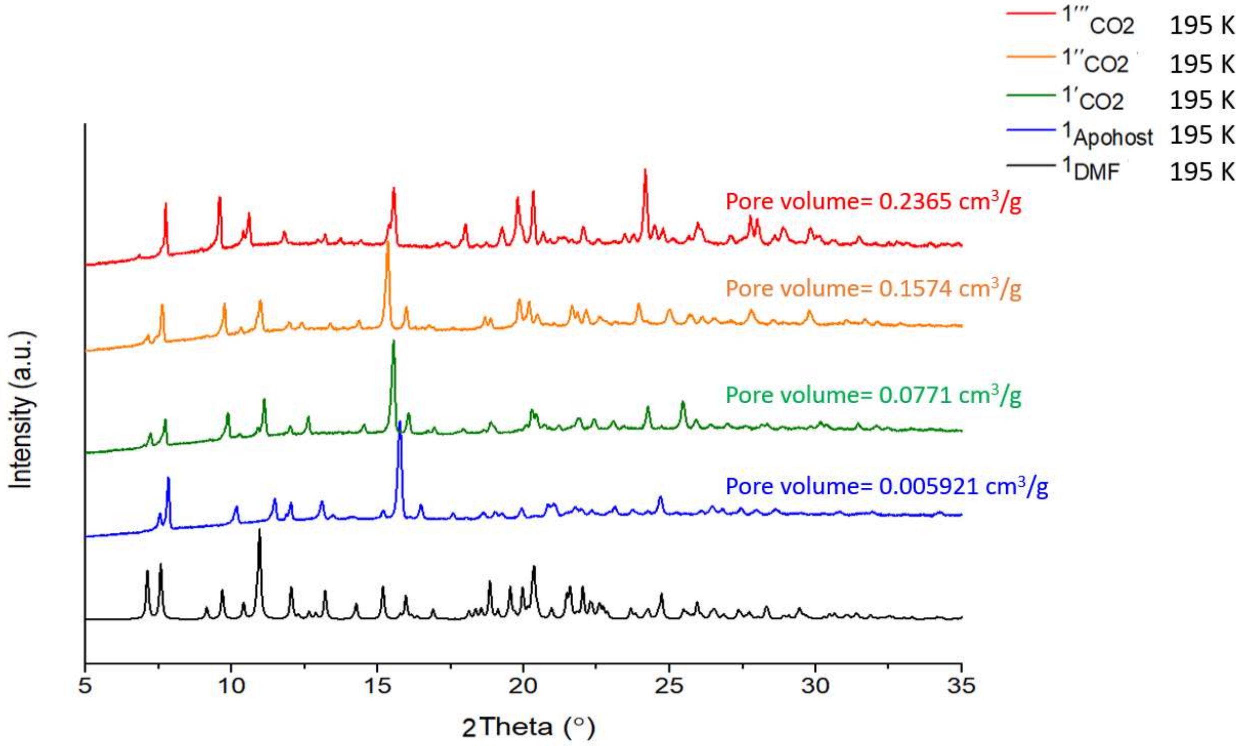

Fig. 31. Overlay of selected PXRD patterns with corresponding pore volumes obtained from the 195 K CO_2 sorption isotherm.

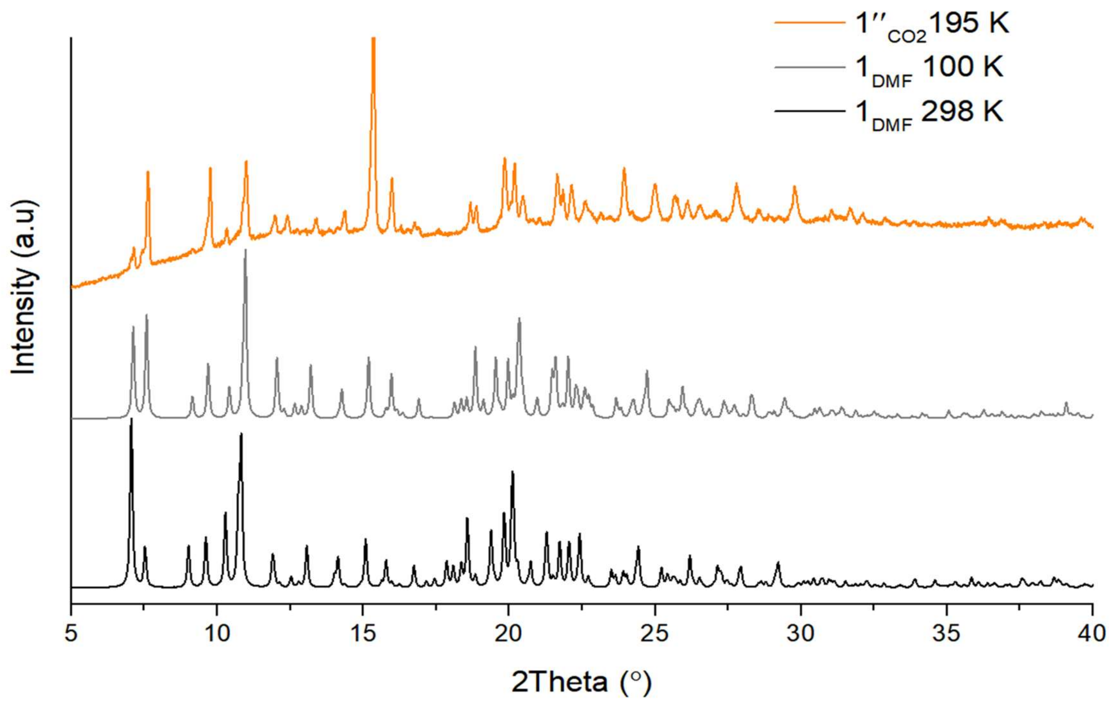

Fig. 32. Overlay of the experimental PXRD pattern for **1″** $_{CO2}$ with the generated PXRD patterns for **1** $_{DMF}$ at 100 K and 298 K. **1″** $_{CO2}$ and **1** $_{DMF}$ appear to be similar phases.

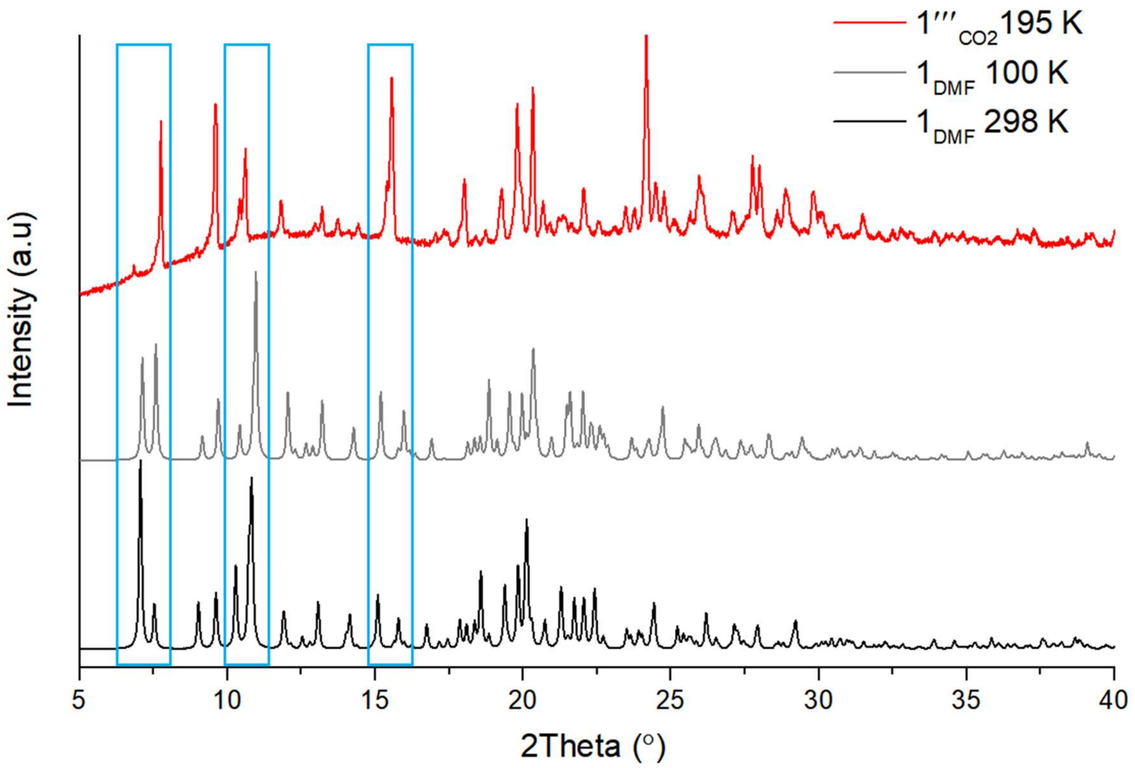

Fig. 33. Overlay of the experimental PXRD pattern for **1‴** $_{CO2}$ with the generated PXRD patterns for **1** $_{DMF}$ at 100 and 298 K. **1‴** $_{CO2}$ and **1** $_{DMF}$ are not the same phase.

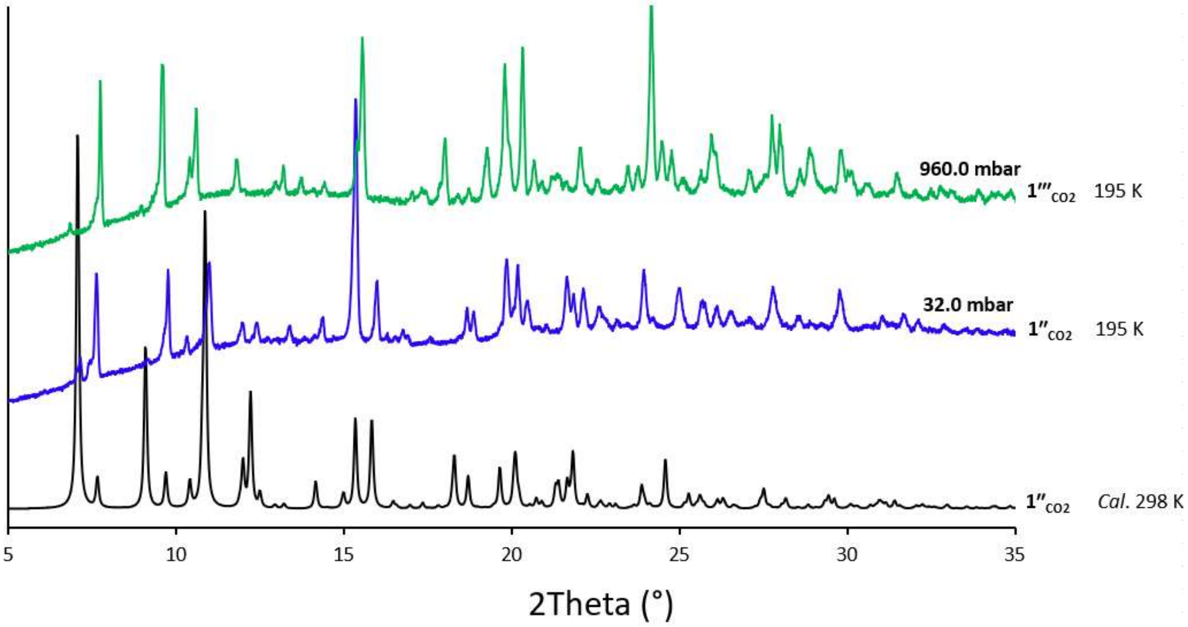

Fig. 34. Overlay of the experimental PXRD pattern for **1‴** $_{\mathbf{CO2}}$ with the experimental PXRD pattern for **1″** $_{\mathbf{CO2}}$ (both collected at 195 K) as well as the generated pxrd pattern for **1″** $_{\mathbf{CO2}}$ at 298 K. The calculated and experimental patterns of **1″** $_{\mathbf{CO2}}$ are in good agreement despite the temperature difference.

Selected powder patterns obtained from the *in situ* variable pressure PXRD experiment of **1**$_{\mathbf{Apohost}}$ at different CO_2 adsorption loadings were indexed using the program EXPO2014.[15]

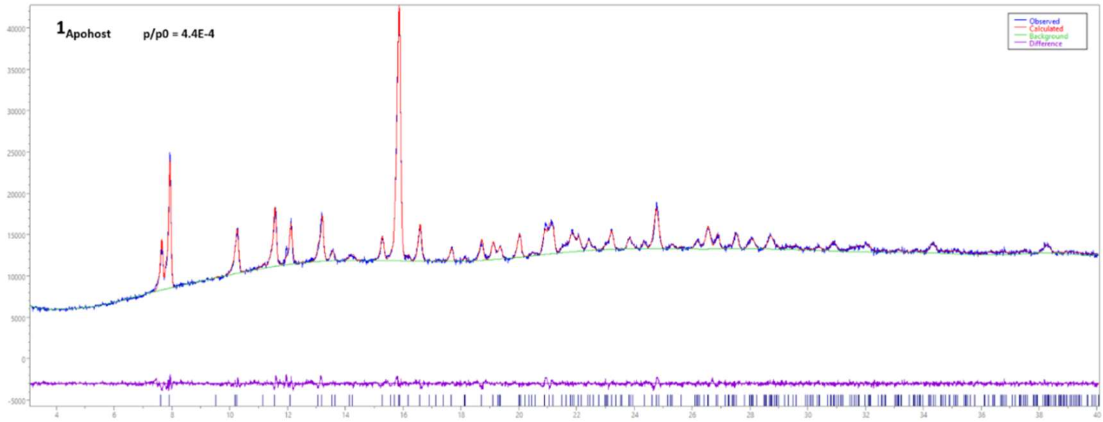

Fig. 35. Indexed PXRD pattern of **1**$_{\mathbf{Apohost}}$ obtained from the *in situ* variable pressure experiment performed for CO_2 at 195 K, P/P_0 is 4.4E-4.

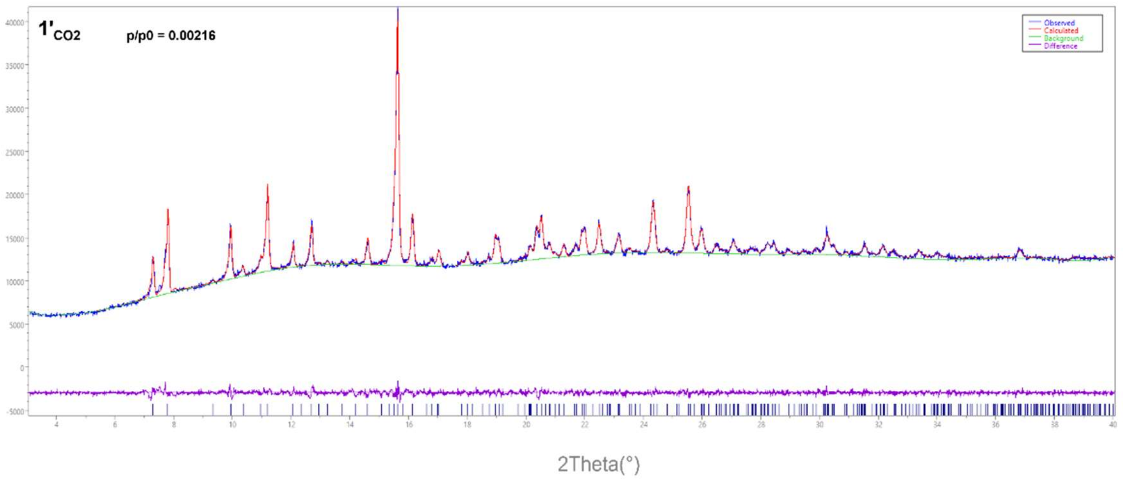

Fig. 36. Indexed PXRD pattern of **1'$_{CO2}$** obtained from the *in situ* variable pressure experiment performed for CO_2 at 195 K, P/P_0 is 2.16E-3.

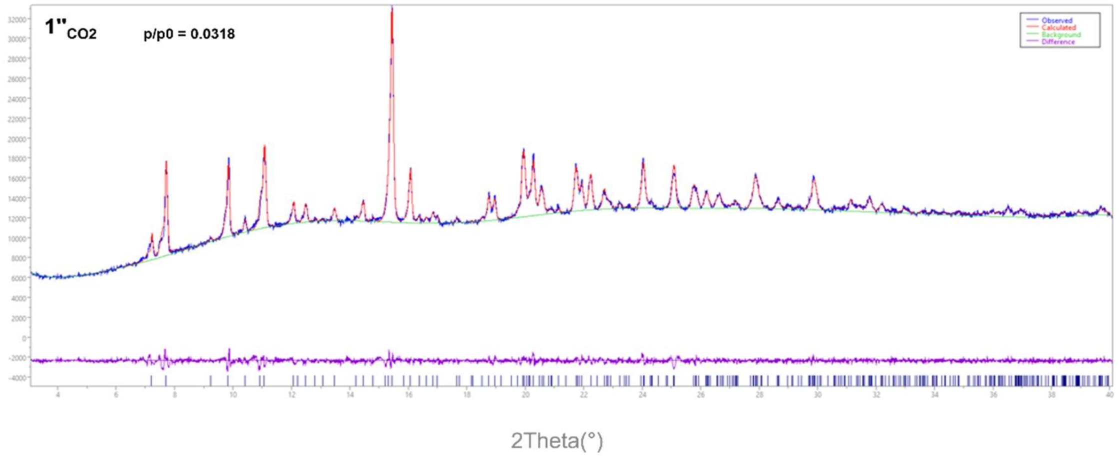

Fig. 37. Indexed PXRD pattern of **1''$_{CO2}$** obtained from the *in situ* variable pressure experiment performed for CO_2 at 195 K, P/P_0 is 3.18E-2.

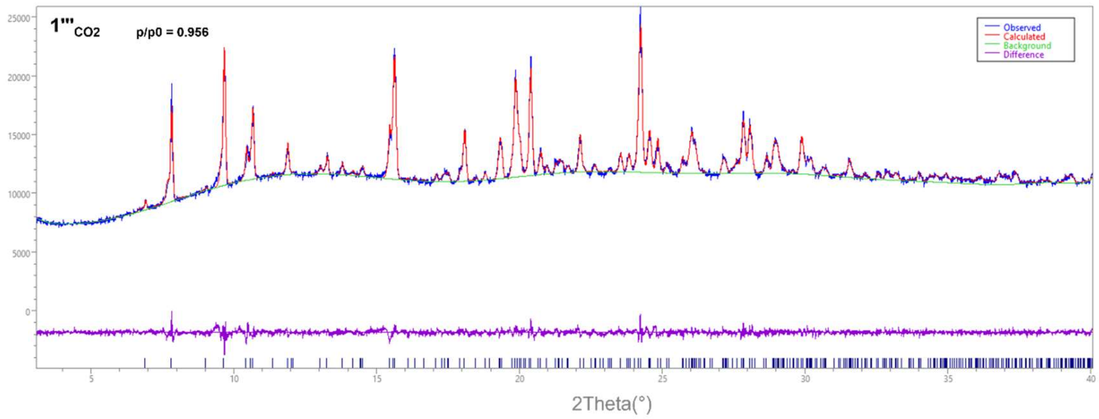

Fig. 38. Indexed PXRD pattern of **1'''$_{CO2}$** obtained from the *in situ* variable pressure experiment performed for CO_2 at 195 K, P/P_0 is 0.956.

Table 4. Crystallographic parameters obtained by indexing selected *in situ* PXRD patterns (adsorption of CO_2 at 195 K) for $\mathbf{1}_{\mathbf{Apohost}}$-$\mathbf{1'''}_{\mathbf{CO2}}$.

	$\mathbf{1}_{\mathbf{Apohost}}$	$\mathbf{1'}_{\mathbf{CO2}}$	$\mathbf{1''}_{\mathbf{CO2}}$	$\mathbf{1'''}_{\mathbf{CO2}}$
Guest	-	CO_2	CO_2	CO_2
Temperature (K)	195 K	195 K	195 K	195 K
Wavelength (Å)	0.71073	0.71073	0.71073	0.71073
Crystal system	Monoclinic	Monoclinic	Monoclinic	Monoclinic
Space group	$P2_1/n$	$P2_1/n$	$P2_1/n$	$P2_1/n$
a/Å	9.824(4)	9.854(a)	9.900(3)	10.095(9)
b/ Å	13.561(9)	14.333(1)	14.492(7)	15.570(9)
c/ Å	22.455(6)	22.897(7)	23.262(4)	23.111(2)
α°	90	90	90	90
β°	95.245	98.20(4)	99.49(7)	101.40(4)
γ°	90	90	90	90
Volume (Å^3)	2979.2(9)	3200.9(7)	3292.0(1)	3561.4(4)
Rp	0.935	0.936	0.955	1.029
Rwp	1.307	1.297	1.370	1.432

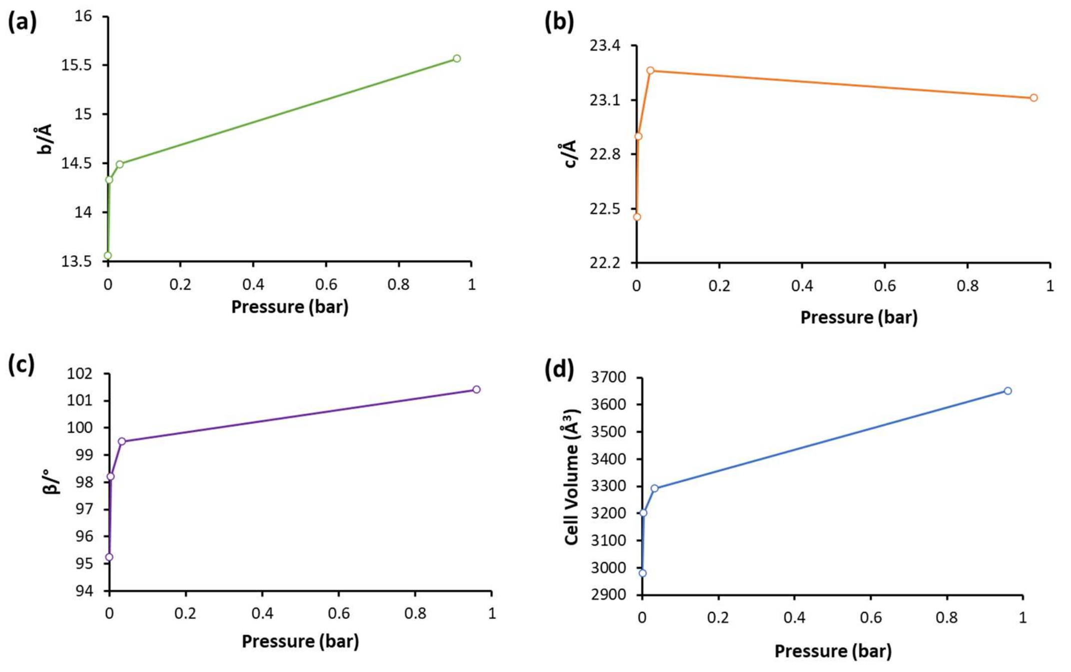

Fig. 39. Changes in indexed cell parameters of $\mathbf{1}_{\mathbf{Apohost}}$ as a function of CO_2 gas pressure at 195 K

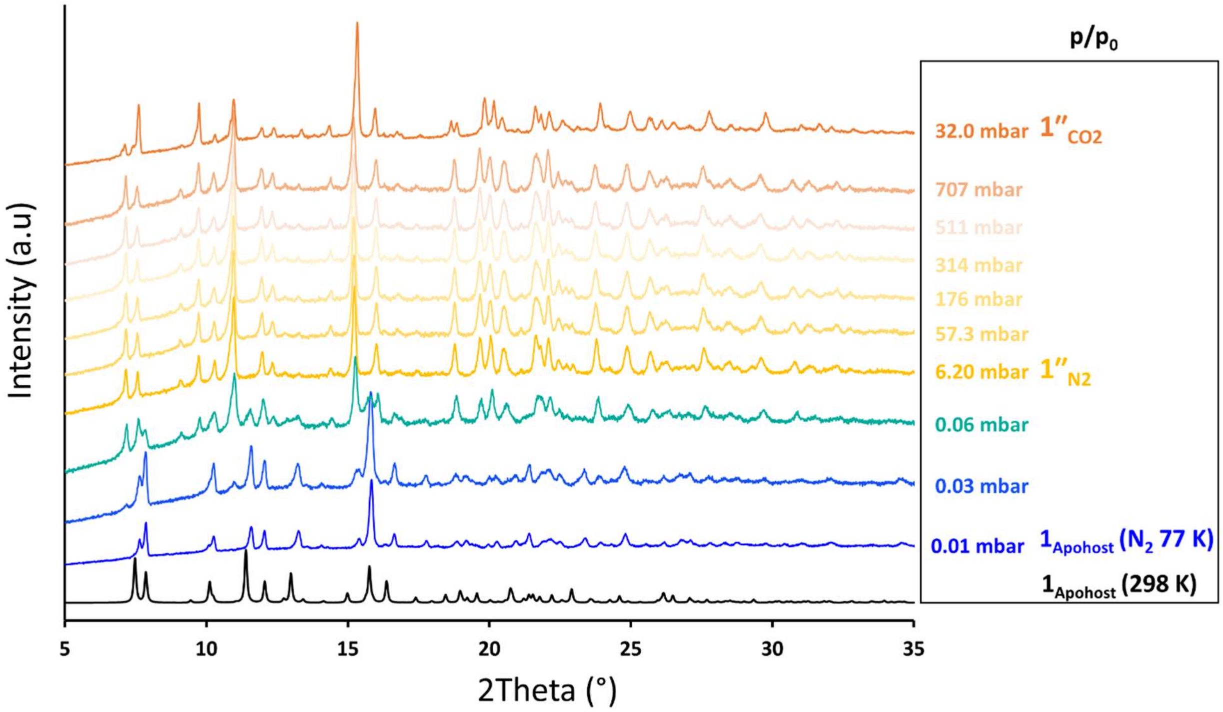

Fig. 40. Selected *in situ* variable pressure PXRD patterns of **1_{Apohost}** at different N_2 adsorption loadings at 77 K. $\mathbf{1_{Apohost}}$ undergoes a structural transformation to the gas loaded $\mathbf{1''_{N2}}$ phase. The PXRD pattern of $\mathbf{1''_{N2}}$ is in good agreement with that of $\mathbf{1''_{CO2}}$.

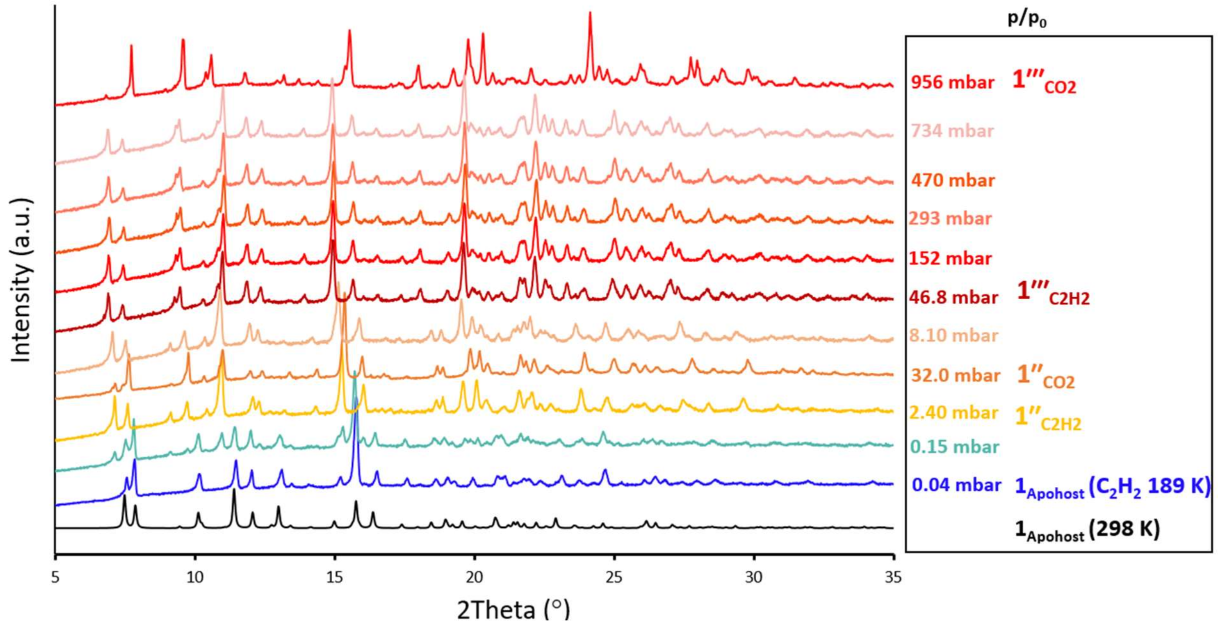

Fig. 41. Selected *in situ* variable pressure PXRD patterns of $\mathbf{1_{Apohost}}$ at different C_2H_2 adsorption loadings at 189 K. $\mathbf{1_{Apohost}}$ undergoes multiple structural transformations from closed to two progressively more gas loaded forms $\mathbf{1''_{C2H2}}$ and $\mathbf{1'''_{C2H2}}$. Comparison of the PXRD pattern of $\mathbf{1'''_{C2H2}}$ with that of $\mathbf{1'''_{CO2}}$ shows that the two phases are not the same.

9. Switching materials reference table

Table 5. N_2 (77 K, 1 bar) and CO_2 (195 K, 1 bar) sorption parameters for representative switching coordination networks.

Coordination Networks	N_2 77 K		CO_2 195 K		Pore Nature	Ref.
	P_{ga} (kPa)	Uptake (cm^3/g)	P_{ga} (kPa)	Uptake (cm^3/g)		
DUT-8(Ni)	13	670	35	590	channel	16
Co(bdp)	3	668	n/a	n/a	channel	17
Fe(bdp)	0.8	638	n/a	n/a		
Co(F-bdp)	4	582	n/a	n/a	channel	18
Co(o-F_2-bdp)	4	560	n/a	n/a		
Co(p-F_2-bdp)	1	504	n/a	n/a		
Cu(HIsa-az-dmpz)	3	360	8	310	channel	19
JLU-Liu3	41	325	26 (F-II)	337	channel	20
JLU-Liu4	26	306	16 (F-II)	333		
JLU-Liu33	6	319	4 (F-II)	205		
X-dia-1-Ni	Remains closed		Type II	325	channel	21
X-pcu-5-Zn	45	135	30	254	channel	22-23
X-pcu-6-Zn	20	290	17	245		
X-pcu-7-Zn	68	217	33	267		
X-pcu-8-Zn	Remains closed		39	243		
ELM-11	10	250	30	245	channel	24-25
ELM-13	20	237	50	180	channel	26
CPM-325	226	216	2.6	200	channel	27
[Cu_2(bdc)$_2$(bpy)]	0.07	170	7	156	channel	28-29
SIFSIX-23-Cu	19	160	3.7	216	channel	30
Cd(bpndc)(bpy)	55	150	n/a		channel	31
Cd(bpndc)(bpy)	n/a		n/a		Discrete cavity	32
[Zn_2(tdc)$_2$(pvq)]	15	140	n/a		channel	33
MIL-53(Sc)	Remains closed		75	285	channel	34
Zn_2(tp)$_2$(dfbpb)	Remains closed		40	248	channel	35
Cu(OTf)$_2$(bpp)$_2$	Remains closed		60	153	channel	36
sql-1-Co-NCS	Remains closed		10	136	channel	37
Zn(GA)$_2$	Remains closed		5	132	channel	38
Cd(mida)$_2$	n/a		35	130	channel	37
SNU-M11	Remains closed		18	124	channel	39
f-MOF-1b	Remains closed		45	107	channel	40
Cu(pyrdc)(bpp)	Remains closed		23	100	channel	41
MIL-53(Fe)	n/a		5	95	channel	42-44
$1_{apohost}$	0.003	123	0.08	157	Discrete cavities	
[a] N.A. = not available; Pga = gate adsorption pressure						

10. Computational Modelling

Computational modelling of the **1** crystal phases using electronic structure methods was undertaken *via* density function theory (DFT). Crystal structures were treated to full periodic relaxation using MOLOPT basis sets at the tripple zeta level of theory & PBE pseudopotentials. Dispersion was treated using a pair potential with the DFT-D3 correction of Grimme *et. al*. These were implemented by the CP2K simulation package.[45-58] DFTMD simulations were implemented similarly using the canonical ensemble (NVT) with a 0.5 fs timestep. The chosen thermostat was Nose Hoover using a timeconstant of 100 fs. Classical modelling of CO_2 sorption in **1** was undertaken to examine the sorptive behaviour and uptakes in these structures. These simulations involved Monte Carlo simulation of sorbates within a rigid crystal scaffold as employed by the Massively Parallel Monte Carlo Code (MPMC).[59] Intermolecular forces were parametrized using the Universal Force Field (UFF),[60] the extended charge equilibration method,[61] and Thole-Applequist type point polarizabilities[62-63] taken from the work of van Duijnen and Swart[64] to model the van der Waals, electrostatic, and induced dipole effects, respectively. The CO2-PHAST*[65] was used for the sorbate parameters. X-ray crystal structures of $\mathbf{1}_{\mathbf{Apohost}}$, $\mathbf{1'}_{\mathbf{CO2}}$, and $\mathbf{1''}_{\mathbf{CO2}}$ were relaxed using DFT as described above. For all three, there was only minor alteration in atomic positions after optimization, providing stable optimized conformations even for the open $\mathbf{1'}_{\mathbf{CO2}}$, and $\mathbf{1''}_{\mathbf{CO2}}$ structures. Classical simulation in the relaxed structures confirmed that they retained porosity, loaded sorbate into discrete pockets, and supported CO_2 uptakes equal to or in excess of the loadings reached in each structure to induce the next open phase as reported in Fig. 5a. The loaded optimized structures are shown in Fig. 42.

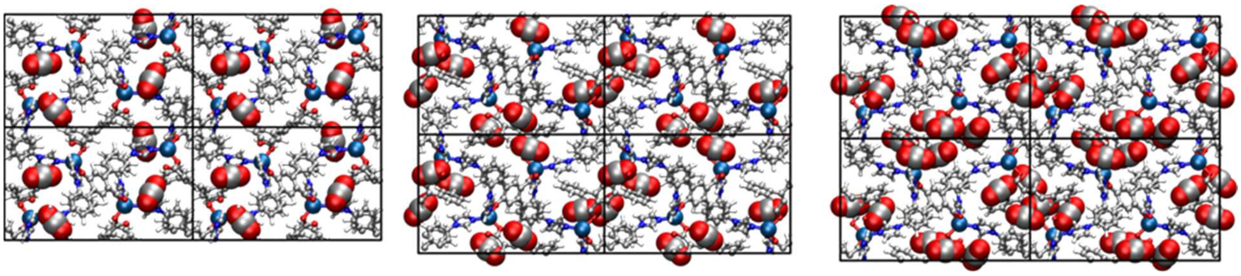

Fig. 42. Classical CO_2 sorption simulation snapshots of three crystal phases of **1** prior to the maximum loading reached before undergoing further conformational shifting, black lines delineate the boundaries of individual unit cells. $\mathbf{1_{Apohost}}$ (left) loaded with 4 CO_2 per unit cell and 1 CO_2 per pore. $\mathbf{1'_{CO2}}$, (center) loaded with 7 CO_2 per unit cell and 3 and 4 in each closed pocket, and $\mathbf{1''_{CO2}}$ (right) at 11 per unit cell loading with 5 and 6 in each cavity. Atom colors: Co = cobalt, C = gray, O = red, N = blue, H = white

Potential for transient transport between neighbouring isolated cavities was examined along two potential pathways in the $\mathbf{1'_{CO2}}$ structure. An initial position was obtained from canonical Monte Carlo runs of CO_2 sorption. A single sorbate position settled near the posited transport window was selected from each of the trajectories and settled into the local minima *via* simulated annealing. A single unit cell was then taken to probe transport between diagonally adjacent voids as each $\mathbf{1'_{CO2}}$ unit cell contains two cavities which have that relative orientation. For the stacked cavities a unit cell was replicated along the *c*-axis to create a two unit cell system with a single sorbate molecule (two cells were used as the stacked relative orientation is not observed within a single unit cell). These systems were then fully relaxed using CP2K. Using these relaxed initial positions, a series of sequential relaxations were performed wherein the optimized structure was modified, shifting the sorbate in the direction of the postulated transport channel and re-relaxed subject to the constraint that a single sorbate oxygen atom was constrained in one direction (the *b*-axis for the diagonally adjacent and the *a*-axis for the stacked). This forced proximity to the channel while allowing the atom to shift in the remaining two dimensions in order to find the optimal route through. No further constraints were put on the other sorbate atoms or the crystal, permitting sorbate orientation and geometry to relax along with the structure. The constrained optimized structures were then taken, the CO_2 shifted further along the channel and re-relaxed. These iterations were repeated until a trajectory traversing each channel was obtained. The resultant trajectories are shown in animations 1-4 below. Having determined the pathways, the energies of each sequential configuration were then

compared with the initial position energy to determine the energy barrier as reported in the "Computational insight into the mechanism of guest transport" section of the main text.

Dynamic molecular motion between cavities in the **1'$_{CO2}$** structure was modelled using a single unit cell with one cavity loaded with 12 CO_2 molecules and the second cavity left empty. Initial positions of sorbate occupants was taken from grand canonical Monte Carlo. The single cavity loaded structure was subjected to DFTMD simulations allowing all atomic positions to fluctuate at 473 K and 623 K. Sorbate loading differential between cavities was found to provide sufficient motive impetus for sorbate molecules to diffuse through the barrier between diagonally adjacent cavities. Simulation across both temperatures reproduced the more energetically favorable mechanism of diffusion via rotation of the dpt linker.

Animation 1. CO_2 trajectory moving between diagonally adjacent cavities in a supercell. Atom colours: Co = cobalt, C = grey, O = red, N = blue, H = white

Animation 2. CO_2 trajectory moving between diagonally adjacent cavities showing only proximal functionality. Atom colours: Co = cobalt, C = grey, O = red, N = blue, H = white

Animation 3. CO_2 trajectory moving between stacked cavities in a supercell. Atom colours: Co = cobalt, C = grey, O = red, N = blue, H = white

Animation 4. CO_2 trajectory moving between stacked cavities showing only proximal functionality. Atom colours: Co = cobalt, C = grey, O = red, N = blue, H = white

Animation 5. Molecular dynamics trajectory of a CO_2 in the **1'$_{CO2}$** structure with a single cavity loaded with 12 sorbate molecules. Dynamic motions include migration from the loaded cavity to unoccupied diagonally adjacent cavity at 473 K. Atom colors: Co = ice blue, C = gray, O = red, N = blue, H = white

Animation 6. Molecular Dynamics trajectory of a CO_2 in the **1'$_{CO2}$** structure with a single pore loaded with 12 sorbate molecules. Dynamic motions include migration from the loaded cavity to unoccupied diagonally adjacent cavity at 623 K. Atom colors: Co = ice blue, C = gray, O = red, N = blue, H = white

11. References

1. Collado, A.; Bohnenberger, J.; Olivia-Madrid, M-J.; Nun, P.; Cordes, D. B.; Slawin, A. M. Z.; Nolan, S. P.; *Eur. J. Inorg. Chem.*, **2016**, 25, 4111-4122.
2. Merlet, S.; Birau, M.; Wang, Z.Y. *Org. Lett.* **2002**, 4, 2157-2159.
3. SMART Data Collection Software, Version 5.629, Bruker AXS Inc., Madison, WI, 2003.
4. SAINT Data Reduction Software, Version 6.45, Bruker AXS Inc., Madison, WI, 2003.
5. SADABS, Bruker AXS Inc., Madison, WI, 2014.
6. Sheldrick, G. M. *Acta Crystallogr., Sect. C*, **2015**, 71, 3-8.
7. Barbour, L. J. *J. Appl. Cryst*., **2020**, *53,* 1141-1146.
8. a) Connolly, M. L. *Science*, **1983**, 221, 709-713; b) Connolly, M. L. *J. Mol. Graphics*, **1993**, 11, 139-141.
9. Degen, T.; Sadki, M.; Bron, E.; König, U.; Nénert, G. *Powder Diffr*. **2014**, 29, S13-S18.
10. Macrae, C. F.; Bruno, I. J.; Chisholm, J. A.; Edgington, P. R.; McCabe, P.; Pidcock, E.; Rodriguez-Monge, L.; Taylor, R.; van de Streek, J.; Wood, P. A. J. Appl. Crystallogr. **2008**, 41, 466-470.
11. Thommes, M. Kaneko, K. Neimark, A. V. Olivier, J. P. Rodriguez-Reinoso, F. Rouquerol, J. Sing, K. S. W. *Pure Appl. Chem*., **2015**; 87, 1051-1069.
12. Gurvich, L., *J. Phys. Chem. Soc. Russ*. **1915**, 47, 49–56.
13. Islamoglu, T.; Idrees, k. B.; Son, F. A. ; Chen, Z.; Lee, S.-J.; Li, P.; and Farha, O.K.; *J. Mater. Chem. A*, **2022**, 10, 157–173.
14. Li, L.; Krishna, R.; Wang, Y.; Yang J.; Wanga, X.; Li, J. *J. Mater. Chem. A*, **2016**, 4, 751-755.
15. a) Altomare, A.; Cuocci, C.; Giacovazzo, C.; Moliterni, A.; Rizzi, R.; Corriero N. and Falcicchio, A. **2013**. *J. Appl. Cryst*. 46, 1231-1235; b) Altomare, A.; Camalli, M.; Cuocci, C.; Giacovazzo, C.; Moliterni, A.; Rizzi, R. **2009**. *J. Appl. Cryst*. 42, 1197-1202.
16. Klein, N.; Hoffmann, H. C.; Cadiau, A.; Getzschmann, J.; Lohe, M. R.; Paasch, S.; Heydenreich, T.; Adil, K.; Senkovska, I.; Brunner E. and Kaskel, S., *J. Mater. Chem*., **2012**, 22, 10303–10312.
17. Mason, J. A.; Oktawiec, J.; Taylor, M. K.; Hudson, M. R.; Rodriguez, J.; Bachman, J. E.; Gonzalez, M. I.; Cervellino, A.; Guagliardi, A.; Brown, C. M.; Llewellyn, P. L.; Masciocchi N.; and Long, J. R., *Nature*, **2015**, 527, 357–361.

18. Taylor, M. K.; Runčevski, T.; Oktawiec, J.; Gonzalez, M. I.; Siegelman, R. L.; Mason, J. A.; Ye, J.; Brown C. M.; and Long, J. R., *J. Am. Chem. Soc.*, **2016**, 138, 15019–15026.

19. Millan, S.; Gil-Hernandez, B.; Milles, E.; Gokpinar, S.; Makhloufi, G.; Schmitz, A.; Schlusener C. and Janiak, C., *Dalton Trans.*, **2019**, 48, 8057–8067.

20. Wang, J.; Luo, J.; Zhao, J.; Li, D.-S.; Li, G.; Huo Q. and Liu, Y., *Cryst. Growth Des.*, **2014**, 14, 2375–2380.

21. Yang, Q. Y.; Lama, P.; Sen, S.; Lusi, M.; Chen, K. J.; Gao, W. Y.; Shivanna, M.; Pham, T.; Hosono, N.; Kusaka, S.; Perry IV, J. J.; Ma, S.; Space, B.; Barbour, L. J.; Kitagawa S. and Zaworotko, M. J., *Angew. Chem.*, Int. Ed., **2018**, 57, 5684–5689.

22. Zhu, A.-X.; Yang, Q.-Y.; Kumar, A.; Crowley, C.; Mukherjee, S.; Chen, K.-J.; Wang, S.-Q.; O'Nolan, D.; Shivanna M., and Zaworotko, M. J., *J. Am. Chem. Soc.*, **2018**, 140, 15572–15576.

23. Zhu, A.-X.; Yang, Q.-Y.; Mukherjee, S.; Kumar, A.; Deng, C.-H.; Bezrukov, A. A.; Shivanna M., and Zaworotko, M. J., *Angew. Chem.*, Int. Ed., **2019**, 58, 18212–18217.

24. Kanoh, H.; Kondo, A.; Noguchi, H.; Kajiro, H.; Tohdoh, A.; Hattori, Y.; Xu, W.-C.; Inoue, M.; Sugiura, T.; Morita, K.; Tanaka, H.; Ohba T.; and Kaneko, K.; *J. Colloid Interface Sci.*, **2009**, 334, 1–7.

25. Ichikawa, M.; Kondo, A.; Noguchi, H.; Kojima, N.; Ohba, T.; Kajiro, H.; Hattori Y.; and Kanoh, H., *Langmuir*, **2016**, 32, 9722–9726.

26. Kondo, A.; Kajiro, H.; Nakagawa, T.; Tanaka H.; and Kanoh, H.; *Dalton Trans.*, **2020**, 49, 3692–3699.

27. Jin, J.; Zhao, X; Feng P., and Bu, X.; *Angew. Chem.*, Int. Ed., **2018**, 57, 3737–3741.

28. Seki, K.; *Phys. Chem.*, **2002**, 4, 1968–1971.

29. Sakata, Y.; Furukawa, S.; Kondo, M.; Hirai, K.; Horike, N.; Takashima, Y.; Uehara, H.; Louvain, N.; Meilikhov, M.; Tsuruoka, T.; Isoda, S.; Kosaka, W.; Sakata O.; and Kitagawa, S.; *Science*, **2013**, 339, 193–196.

30. Song, B.-Q.; Yang, Q.-Y.; Wang, S.-Q.; Vandichel, M.; Kumar, A.; Crowley, C.; Kumar, N.; Deng, C.-H.; Gascon Perez, V.; Lusi, M.; Wu, H.; Zhou W.; and Zaworotko, M. J.; *J. Am. Chem. Soc.*, **2020**, 142, 6896–6901.

31. Tanaka, D.; Nakagawa, K.; Higuchi, M.; Horike, S.; Kubota, Y.; Kobayashi, T. C.; Takata M.; and Kitagawa, S., *Angew. Chem.*, Int. Ed., **2008**, 47, 3914–3918.

32. van Heerden, D. P.; Smith, V. J.; Aggarwal H.; and Barbour L. J., *Angew. Chem.* Int. Ed. **2021**, 60, 13430–13435.

33. Shi, Y.-X.; Li, W.-X.; Zhang W.-H.; and Lang, J.-P.; *Inorg. Chem*., **2018**, 57, 8627–8633.

34. Chen, L.; Mowat, J. P. S.; Fairen-Jimenez, D.; Morrison, C. A.; Thompson, S. P.; Wright P. A.; and Duren, T., *J. Am. Chem. Soc*., **2013**, 135, 15763–15773.

35. Seo, J.; Bonneau, C.; Matsuda, R.; Takata M.; and Kitagawa, S., *J. Am. Chem. Soc*., **2011**, 133, 9005–9013.

36. Fukuhara, K.; Noro, S.-i.; Sugimoto, K.; Akutagawa, T.; Kubo K.; and Nakamura, T., *Inorg. Chem*., **2013**, 52, 4229–4237.

37. Wang, S.-Q.; Yang, Q.-Y.; Mukherjee, S.; O'Nolan, D.; Patyk-Kazmierczak, E.; Chen, K.-J.; Shivanna, M.; Murray, C.; Tang C. C.; and Zaworotko, M. J., *Chem. Commun*., **2018**, 54, 7042–7045.

38. Rabone, J.; Yue, Y. F.; Chong, S. Y.; Stylianou, K. C.; Bacsa, J.; Bradshaw, D.; Darling, G. R.; Berry, N. G.; Khimyak, Y. Z.; Ganin, A. Y.; Wiper, P.; Claridge J. B.; and Rosseinsky, M. J., *Science*, **2010**, 329, 1053–1057.

39. Yang, H.; Guo, F.; Lama, P.; Gao, W.-Y.; Wu, H.; Barbour, L. J.; Zhou, W.; Zhang, J.; Aguila, B.; and Ma, S., *ACS Cent. Sci*., **2018**, 4, 1194–1200.

40. Choi H. S., and Suh, M. P., *Angew. Chem*., Int. Ed., **2009**, 48, 6865–6869.

41. Kanoo, P.; Haldar, R.; Reddy, S. K.; Hazra, A.; Bonakala, S.; Matsuda, R.; Kitagawa, S.; Balasubramanian S.; and Maji, T. K., *Chem.–Eur. J*., **2016**, 22,15864–15873.

42. Maji, T. K.; Mostafa, G.; Matsuda R.; and Kitagawa, S., *J. Am. Chem. Soc*., **2005**, 127, 17152–17153.

43. Llewellyn, P. L.; Horcajada, P.; Maurin, G.; Devic, T.; Rosenbach, N.; Bourrelly, S.; Serre, C.; Vincent, D.; Loera-Serna, S.; Filinchuk Y.; and Ferey, G., *J. Am. Chem. Soc*., **2009**, 131, 13002–13008.

44. Guillou, N.; Bourrelly, S.; Llewellyn, P. L.; Walton R. I.; and Millange, F., *CrystEngComm*, **2015**, 17, 422–429.

45. Kuehne, T.D.; Iannuzzi, M.; Del Ben, M.; Rybkin, V.V.; Seewald, P.; Stein, F.; Laino, T.; Khaliullin, R.Z.; Schuett, O.; Schiffmann, F.; Golze, D.; Wilhelm, J.; Chulkov, S.; Bani-Hashemian, M.H.; Weber, V.; Borstnik, U.; Taillefumier, M.; Jakobovits, A.S.; Lazzaro, A.; Pabst, H.; Mueller, T.; Schade, R.; Guidon, M.; Andermatt, S.; Holmberg, N.; Schenter, G.K.; Hehn, A.; Bussy, A.; Belleflamme, F.; Tabacchi, G.; Gloess, A.; Lass, M.;

Bethune, I.; Mundy, C. J.; Plessl, C.; Watkins, M.; Vande Vondele, J.; Krack, M.; Hutter, J.; *Chem. Phys.*, **2002**, 152, 19.

46. Goerigk, L.; Hansen, A.; Bauer, C.; Ehrlich, S.; Najibi, A.; Grimme, S.; *Chem. Phys*., **2017**, 19, 48.

47. Schuett, O.; Messmer, P.; Hutter, J.; VandeVondele, J.; J*ohn Wiley & Sons*, *Ltd*, **2016**, 173-190.

48. Borstnik, U.; VandeVondele, J.; Weber, V.; Hutter, J.; *Parallel Compute*., **2014**, (5-6), 47-58 (2014).

49. Hutter, J.; Iannuzzi, M.; Schiffmann, F.; VandeVondele, J.; *WIREs Comput Mol Sci.*, **2014**, 4 (1), 15-25.

50. Marek, A.; Blum, V.; Johanni, R.; Havu, V.; Lang, B.; Auckenthaler, T.; Heinecke, A.; Bungartz, H.; Lederer, H.; *J. Phys. Condens. Matter*., **2014**, 26 (21).

51. Grimme, S.; Ehrlich, S.; Goerigk, L. J.; *Comput. Chem*., **2011**, 32, 1456.

52. Grimme, S.; Antony, J.; Ehrlich, S.; Krieg, H.; *Chem. Phys.*, **2010** 132 (15), 154104.

53. VandeVondele, J.; Hutter, J.; *Chem. Phys*., **2007**, 127 (11), 114105.

54. Kuhne, TD.; Krack, M.; Mohamed, F.R.; Parrinello, M.; *Phys. Rev. Lett.*, **2007**, 98 (6), 066401.

55. Krack, M.; *Theor. Chem. Acc*., **2005**, 114 (1-3), 145-152.

56. VandeVondele, J.; Krack, M.; Mohamed, F.; Parrinello, M.; Chassaing, T.; Hutter, J.; *Comput. Phys. Commun.*, **2005**, 167 (2), 103-128.

57. Nose, S; A Unified Formulation of the Constant Temperature Molecular-Dynamics Methods*. J. Chem. Phys.* **1984**, 81 (1), 511-519

58. Nose, S; Molecular-Dynamics Method for Simulation in the Canonical Ensemble. *Mol. Phys.* **1984**, 52, (2), 255-268.

59. Belof, Jon L., Brian Space, "Massively Parallel Monte Carlo(MPMC)", Available on GitHub.

60. Rappé, Anthony K., Carla J. Casewit, K. S. Colwell, William A. Goddard III, and W. Mason Skiff. *J. Am. Chem. Soc.,* **1992**, 114, 25, 10024-10035.

61. Wilmer, Christopher E., Ki Chul Kim, and Randall Q. Snurr. *J. Phys. Chem*., **2012**, 17 2506-2511.

62. Thole, B. Th., *Chem. Phys*. **1981,** 59, 3, 341-350.

63. Applequist, Jon, James R. Carl, and Kwok-Kueng Fung. *J. Am. Chem. Soc.* **1972,** 94, 9, 2952-2960.

64. Van Duijnen, Piet Th, and Marcel Swart. Am. *J. Phys. Chem.* **1998**, 102, 14, 2399-2407.

65. Mullen, Ashley L., Tony Pham, Katherine A. Forrest, Christian R. Cioce, Keith McLaughlin, and Brian Space., *J. Chem. Theory Comput.* **2013**, 9, 12, 5421-5429.
